# Force‐Induced Control of Circularly Polarized Luminescence With Rotaxane Architecture

**DOI:** 10.1002/anie.9590374

**Published:** 2026-07-24

**Authors:** Keigo Nonaka, Takumi Kuroda, Kota Masuda, Toshiki Nishitani, Masaki Enokido, Makoto Tsurui, Yuichi Kitagawa, Yasuchika Hasegawa, Keiichi Noguchi, Koji Nakano, Yoshimitsu Sagara

**Affiliations:** ^1^ Department of Materials Science and Engineering Institute of Science Tokyo, Meguro‐ku Tokyo Japan; ^2^ Graduate School of Chemical Sciences and Engineering Hokkaido University Sapporo Hokkaido Japan; ^3^ Faculty of Engineering Hokkaido University Sapporo Hokkaido Japan; ^4^ Institute for Chemical Reaction Design and Discovery (WPI‐ICReDD) Hokkaido University Kita 21, Nishi 10, Kita‐ku Sapporo Hokkaido Japan; ^5^ Instrumentation Analysis Center Tokyo University of Agriculture and Technology Koganei Tokyo Japan; ^6^ Department of Applied Chemistry Tokyo University of Agriculture and Technology 2‐24‐16 Naka‐cho Koganei Tokyo Japan; ^7^ Research Center for Autonomous Systems Materialogy (ASMat) Institute of Integrated Research, Institute of Science Tokyo Yokohama Kanagawa Japan

**Keywords:** circularly polarized luminescence, gels, mechanoresponsive luminescence, rotaxanes, supramolecular mechanophores

## Abstract

The reversible molecular motions of mechanically interlocked molecules (MIMs) have played a crucial role in realizing stimuli‐responsive functions. While the reversible switching of circularly polarized luminescence (CPL) properties has been achieved using MIMs through pH change and/or addition of chemicals, the mechanically controlled CPL utilizing the interlocked architecture has not yet been demonstrated. Here, we introduce a rotaxane‐based mechanophore designed for force‐induced CPL on/off switching. The mechanophore consists of a ring featuring a CPL‐active helicene luminophore and an axle with a matching quencher. The rotaxane mechanophores covalently introduced at the cross‐linking points of a double‐network (DN) gel are reversibly activated and deactivated in response to changes in applied force transduced through the polymer chains during the macroscopic swelling and shrinking of the DN gel. The isotropic swelling of the DN gel effectively suppresses artifacts arising from macroscopic sample orientation, enabling accurate detection of reciprocal CPL derived from a single chiral emitter. This work establishes the first example of mechanical control of CPL at the single‐molecule level and provides a robust measurement methodology for force‐induced CPL switching in soft materials.

## Graphical abstract

Force‐induced reversible on/off switching of circularly polarized luminescence is achieved with a rotaxane‐based supramolecular mechanophore. The mechanophore, featuring a chiral helicene luminophore, is incorporated as a first network cross‐linker into a double‐network organogel. Isotropic swelling applies force to activate the mechanophore via polymer chains, whereas shrinking induces relaxation to deactivate the mechanophore.

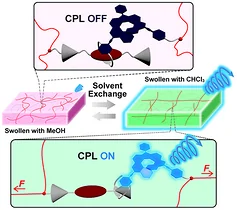

## 1 Introduction

Molecular materials that alter their properties and functions in response to external stimuli have been intensively investigated in recent years. Mechanically interlocked molecules (MIMs), including rotaxanes, catenanes, and molecular knots, have been developed over several decades and enable reversible motions such as molecular shuttling and rotation without cleavage of covalent bonds [1, 2, 3]. Owing to their exceptional design flexibility as well as predictable and reversible motion, MIMs have provided a versatile platform for the development of stimuli‐responsive molecular materials that perform programmed functions, including molecular switches [4, 5, 6, 7, 8, 9, 10, 11], pumps [12, 13], logic gates [14], information ratchets [15], and controlled drug delivery [16, 17]. Among the functions emerging from the unique motions of MIMs, the switching of chiroptical properties, and in particular circularly polarized luminescence (CPL), is especially attractive. CPL is a photophysical phenomenon with potential applications in advanced optical technologies [18], including 3D displays [19], security inks [20], and chirality sensing of biomolecules [21, 22]. The reversible switching of CPL properties by external stimuli is essential to the development of next‐generation smart materials that exploit this chiroptical functionality across a broad range of applications, and such switching could be realized by harnessing the characteristic motions of MIMs.

Indeed, several MIMs achieve CPL switching through external stimuli‐induced changes in molecular arrangement [23]. For example, Blanco and coworkers demonstrated that a [2]rotaxane bearing a chiral secondary amine on the axle shows CPL on/off switching through acid/base cycles retaining the fluorescence emission [24]. Yang, Wang, and coworkers have also described rotaxanes [25, 26], catenanes [27, 28], and [*c*2]daisy chains [29] that reversibly switch their CPL properties in response to pH changes and/or addition of chemicals. These studies demonstrate that MIMs are promising architectures for precise control of CPL properties at the single‐molecule level. However, interlocked molecules capable of modulating CPL properties in response to mechanical force at the single‐molecule level have not yet been reported.

Molecular structures that change their optical properties in response to mechanical force are referred to as mechanochromic mechanophores, and have been extensively investigated because such mechanophores can function as molecular probes to visualize stress distribution within polymer materials [30, 31, 32, 33, 34]. Most conventional mechanophores require covalent bond cleavage for activation and thus exhibit irreversible changes, making them suitable for recording force‐application histories [35, 36, 37, 38, 39, 40, 41, 42, 43, 44, 45, 46, 47]. By contrast, mechanophores that do not rely on covalent bond scission typically undergo instantaneous and reversible changes in photophysical properties, which enable real‐time visualization of forces applied to polymer chains [48, 49, 50, 51, 52, 53, 54, 55, 56, 57]. As one class of the latter, we have developed supramolecular mechanophores that operate via force‐induced changes in the relative arrangement of luminophores and/or quenchers. In particular, rotaxane mechanophores are notable for their design flexibility [58, 59, 60, 61, 62, 63]. Our prototype rotaxane mechanophore comprises a ring bearing a luminophore and an axle containing an electronically matching quencher at the center and two stoppers at both ends [58]. Hydroxy groups are introduced into the ring and one of the stoppers to covalently incorporate the mechanophore into segmented polyurethane. In the absence of an external force, the ring resides over the quencher, leading to fluorescence quenching through photoinduced electron transfer (PET). When a sufficient force is applied through the polymers attached to the ring and the axle, the ring starts to slide along the axle, leading to force‐induced spatial separation of the luminophore and quencher. This suppresses PET, resulting in intense fluorescence.

On the basis of this operating principle, the rotaxane mechanophore can control other photofunctions beyond simple fluorescence intensity. We recently demonstrated modulation of Förster resonance energy transfer (FRET) efficiency by force [59]. A green‐emissive donor is incorporated into the cyclic structure and a red‐emissive acceptor is attached to the green emitter via a short aliphatic linker. When the green emitter is exclusively excited in the force‐free state, PET from the green emitter to the quencher effectively quenches the green fluorescence and suppresses FRET from the green to the red emitter. Once mechanical force is transduced to the mechanophore through the attached polymer chains, the shuttling motion is initiated, PET is suppressed, and efficient FRET to the red emitter is activated, producing red emission. This success demonstrated that other photophysical phenomena, such as phosphorescence, singlet fission, and CPL, can also be controlled by force with an interlocked architecture.

**FIGURE 1:**
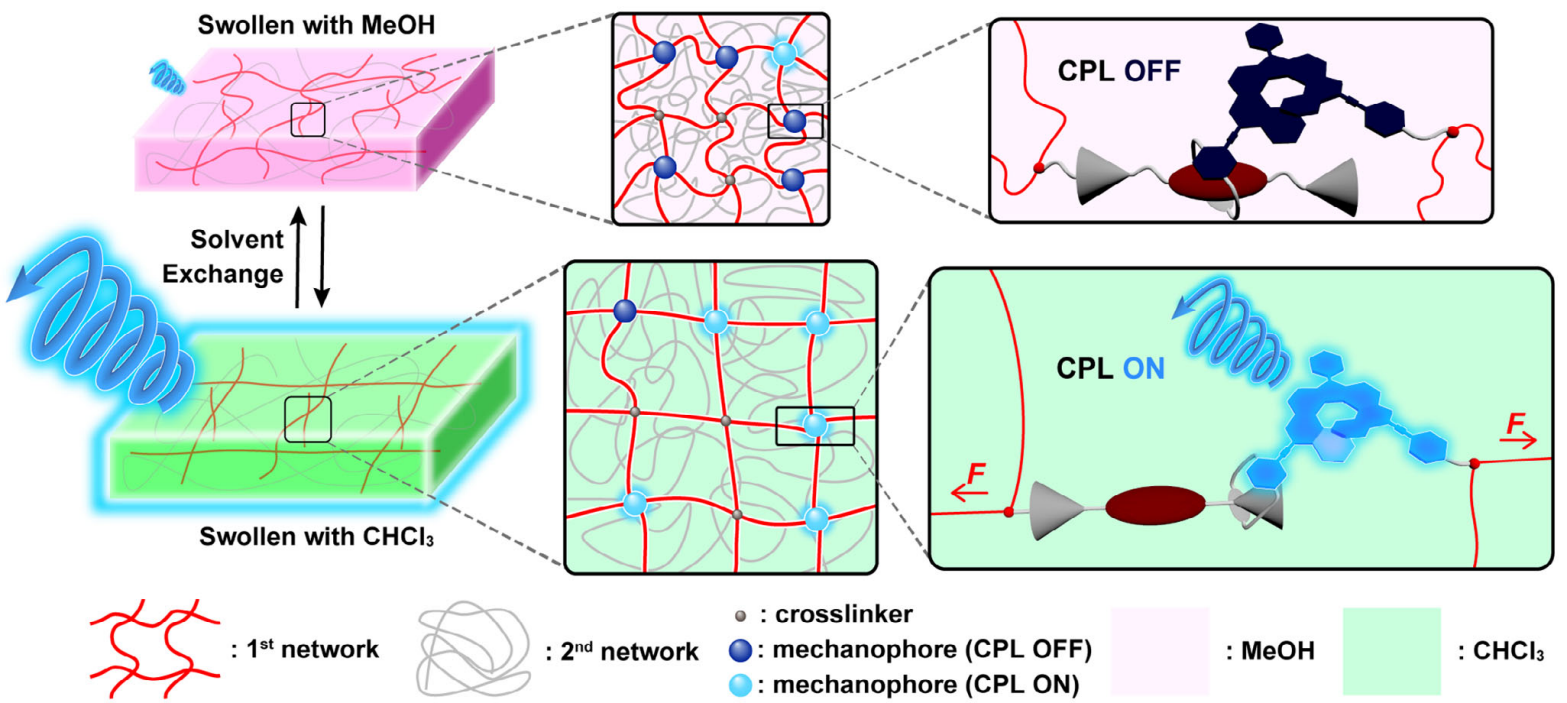
Schematic illustration of the reversible CPL on/off switching of mechanophores covalently embedded in DN gels through solvent exchange. The interlocked architecture consists of a ring containing CPL‐active helicene (blue) and an axle with a matching quencher (brown) and two stoppers (gray). The mechanophore is integrated as a cross‐linker in the first network (red) of the DN gel. In the methanol‐swollen state (top), the CPL‐active ring resides over the quencher due to the low swelling ratio, resulting in a CPL‐off state. In the chloroform‐swollen state (bottom), the isotropic and significant swelling of the gel stretches the first network, inducing a force that separates the ring from the quencher and thus activates CPL.

Herein, we demonstrate force‐induced on/off switching of CPL emission arising from molecular chirality (reciprocal CPL) using a rotaxane‐based mechanophore that incorporates a CPL‐active helicene fluorophore (Figure 1). The reversible switch is realized by embedding the mechanophore in double‐network (DN) gels and modulating the swelling ratio through solvent exchange. Although numerous mechanophores have been reported, those capable of switching CPL properties have not been developed, presumably because their synthesis is challenging and accurate detection of weak CPL signals is difficult. Uniaxial stretching of mechanophore‐embedded polymer films, which is one of the conventional activation methods, often generates strong CPL artifacts originating from linear dichroism and linear birefringence induced by macroscopic molecular orientation (non‐reciprocal CPL). These artifacts prevent accurate evaluation of the weak reciprocal CPL signals derived from luminophores. Therefore, we utilized the isotropic swelling of DN gels to minimize artifacts. DN gels are a class of tough gels renowned for their excellent mechanical properties, which stem from an energy dissipation mechanism involving the sacrificial rupture of the brittle first network while the ductile second network stops crack propagation [64, 65, 66, 67]. During swelling, force is preferentially applied to the first network, which can activate incorporated mechanophores. Indeed, Watabe and Otsuka have reported swelling‐induced mechanophore activation in multinetwork gels [68], where swelling triggers scission of mechanophores integrated as first network cross‐linkers. In the present study, incorporating our mechanophores as cross‐linkers for the first network enables reversible on/off switching of CPL emission during macroscopic swelling and shrinking of the gels by sequential solvent exchanges. The CPL response of bulk DN gels originates from a single chiral luminescent molecule, yielding a reciprocal CPL signal without undesired chiroptical effects induced by anisotropic sample orientation or aggregation of emitters.

Several crystalline materials [69, 70, 71, 72, 73], chiral polymer matrices [74], supramolecular polymers [75], and liquid crystals [76, 77] have been reported to exhibit mechano‐responsive CPL. However, the mechanism is governed by changes in bulk molecular assembly. Very recently, Chen and coworkers reported mechanically induced CPL from poly(*L*(‐)‐bornyl acrylate)s covalently incorporating Diels–Alder adduct mechanophores [78]. Nevertheless, the observed CPL signals were non‐reciprocal in character, arising from anisotropic orientation of the polymer upon compression [79]. Precise and reversible control of reciprocal CPL therefore remains unexplored.

## 2 Results and Discussion

**FIGURE 2:**
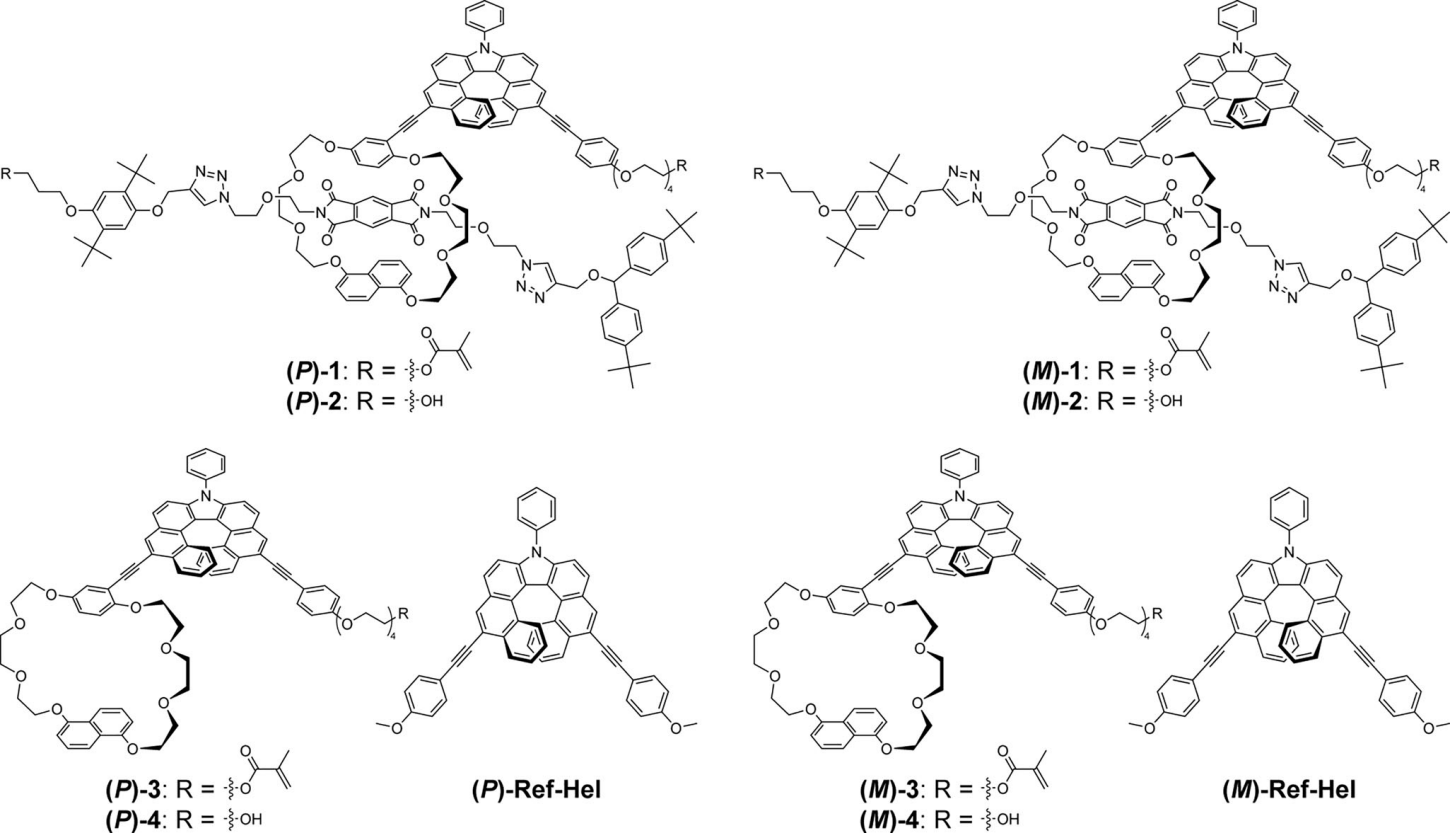
Molecular structures of rotaxane mechanophores ***(P)/(M)*‐1**, hydroxy terminated rotaxanes **(*P*)/(*M*)‐2**, corresponding rings, and reference compounds **(*P*)/(*M*)‐Ref‐Hel**.

The new mechanophore **1** designed to achieve on/off switching of CPL emission in response to mechanical force is a [2]rotaxane composed of a ring molecule bearing a chiral luminophore and an axle molecule containing a quencher and bulky stoppers (Figure 2). 9‐Phenyldinaphtho[2,1‐*c*,1′,2′‐*g*]carbazole (**A7Hel**), which is categorized into aza[7]helicenes [80], was chosen as the chiral luminophore. Helical emitter **A7Hel** derivatives are known to exhibit relatively high luminescence dissymmetry factors (*g*_lum_) and moderate photoluminescence quantum yields among organic CPL emitters [81]. In addition, its high enantiomerization barrier suppresses thermal racemization, ensuring configurational stability during the synthesis and measurements. Moreover, because **A7Hel** possesses locally planar aromatic surfaces within its helical framework, **A7Hel** is suitable for the emitter of rotaxane mechanophores. The low steric hindrance between the emitter and quencher allows them to be in close proximity, leading to efficient fluorescence quenching. Two phenylethynyl groups were attached to **A7Hel** to introduce a ring structure and one flexible tetraethyleneglycol chain. A pyromellitic diimide (PMDI) was used as the matching quencher because of its lower LUMO energy level compared to that of the *π*‐extended **A7Hel** emitter (Figure S1), which enables efficient PET [82]. The HOMO energy levels of the 1,5‐dialkoxynaphthalene and *π*‐extended **A7Hel** are significantly higher than that of PMDI. Charge‐transfer (CT) interactions between the two electron‐rich aromatic groups and electron‐deficient PMDI are suitable for rotaxane formation. 1,4‐Di‐*tert*‐butylbenzene and bis(4‐*tert*‐butylphenyl)methane groups serve as the bulky stoppers at both ends of the axle. Polymerizable methacrylate groups were introduced into both the ring and one terminus of the axle to incorporate the rotaxane mechanophore into the first network of the DN gels as the crosslinker.

To obtain rotaxane mechanophores containing enantiopure helicene groups, the racemic ring ***rac*‐4** featuring a tetraethyleneglycol terminated with a hydroxy group was first synthesized and subjected to optical resolution via preparative chiral HPLC to afford the enantiomers **(*P*)‐4** and **(*M*)‐4** (see Supporting Information). X‐ray single‐crystal analysis was performed for reference compound **(*P*)‐Ref‐Hel** to determine the absolute configurations of the enantiomers (Figures S2 and S3). The absolute configurations of the helicene derivatives were determined by comparison of the circular dichroism (CD) spectra of **(*P*)‐Ref‐Hel** with those of enantiomers **(*P*)‐3** and **(*M*)‐3** bearing a polymerizable group (Figures S4 and S5). Subsequently, using the enantiopure **(*P*)/(*M*)‐4**, the corresponding rotaxanes **(*P*)/(*M*)‐2** with two hydroxy groups were synthesized via the Cu(I)‐catalyzed azide‐alkyne cycloaddition under high‐concentration conditions. Finally, **(*P*)‐2** and **(*M*)‐2** were reacted with methacryloyl chloride to afford polymerizable rotaxane mechanophores **(*P*)‐1** and **(*M*)‐1**, respectively. The racemic rotaxane mechanophore ***rac*‐1** was synthesized from ***rac*‐4** without optical resolution. Almost all aromatic protons on the electron‐rich macrocycle of ***rac*‐2** were assigned based on ^1^H, ^1^H‐^1^H COSY, and ^1^H‐^1^H ROESY NMR spectra, and they clearly exhibited significant upfield shifts compared to those of ring ***rac*‐4** (Figures S6 and S7). This shielding effect is consistent with previously reported rotaxane mechanophores featuring a PMDI quencher [62, 63], confirming the formation of the mechanically interlocked architecture. The pronounced quenching of CPL emission in **(*P*)/(*M*)‐1** also supports the rotaxane formation (see below). It should be noted that rotaxanes ***rac*/(*P*)/(*M*)‐1** and ***rac*/(*P*)/(*M*)‐2** were obtained as a mixture of co‐conformational regioisomers due to the unsymmetrical structures of both the axle and macrocycle, although their analytical reversed‐phase HPLC chromatograms showed a single sharp peak (Figures S8–S10). Crucially, the ratios of these regioisomers are almost the same among ***rac*‐1**, **(*P*)‐1**, and **(*M*)‐1**. This constant composition ensures that the presence of multiple regioisomers does not perturb the bulk chiroptical properties, allowing **(*P*)‐1** and **(*M*)‐1** to exhibit mirror‐image signals. Compounds **3** and **4** were also fully characterized (see Supporting Information).

**FIGURE 3:**
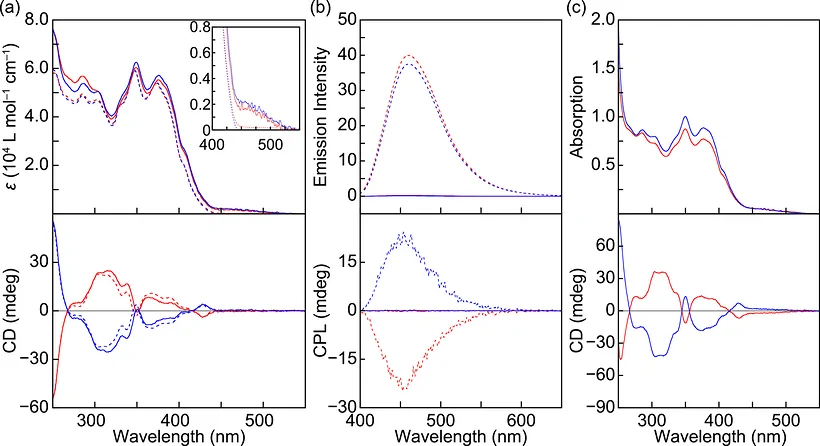
(a) Absorption (top) and CD (bottom) spectra and (b) photoluminescence (top) and CPL (bottom) spectra of **(*P*)‐1** (red solid line), **(*M*)‐1** (blue solid line), **(*P*)‐3** (red dotted line), and **(*M*)‐3** (blue dotted line) in chloroform (*c* = 1.0 × 10^−5^ M). *λ*_ex_ = 330 nm. (c) Absorption (top) and CD (bottom) spectra of **(*P*)‐1‐DN‐DF** (red) and **(*M*)‐1‐DN‐DF** (blue). Inset in panel (a): expansion of the 400–450 nm region.

Chiroptical properties of mechanophores **(*P*)/(*M*)‐1** and reference ring molecules **(*P*)/(*M*)‐3** were investigated in dilute chloroform solutions (*c* = 1.0 × 10^−5^ M) (Figure 3). The absorption spectra of the reference rings **(*P*)/(*M*)‐3** showed a broad band with peaks at 347 nm (*ε* = 5.9 × 10^4^ L mol^−1^ cm^−1^) and 373 nm (*ε* = 5.4 × 10^4^ L mol^−1^ cm^−1^) between 320 and 440 nm (Figure 3a, top). In contrast, the absorption spectra of rotaxanes **(*P*)/(*M*)‐1** showed new tails at a lower energy region between 440 and 500 nm, which are ascribed to the formation of CT complexes between the helicene donor and PMDI acceptor within the interlocked structure. Broad absorption bands originating from CT complexes between PMDI and electron‐rich aromatic rings were previously reported by Sanders and co‐workers [83, 84]. Similar absorption tails were also observed for absorption spectra of our previous rotaxane mechanophores [62, 63]. The enantiomers **(*P*)/(*M*)‐3** showed mirror‐image CD spectra, with **(*M*)‐3** exhibiting a small positive Cotton effect between 440 and 420 nm, and large negative Cotton effects between 410 and 360 nm and between 340 and 270 nm (Figure 3a, bottom). The CD spectral features and signal intensities were well preserved for rotaxanes **(*P*)/(*M*)‐1**, indicating that rotaxane formation has a negligible effect on the chiroptical properties of the ground‐state helicene luminophore. The CD spectroscopy suggested that there was almost no racemization during the rotaxane formation and subsequent introduction of polymerizable groups. The photoluminescence and CPL spectra of rings **(*P*)‐3** and **(*M*)‐3** were recorded using two different emission slit widths to evaluate the chiroptical properties of the excited states. When recorded with a widened emission slit width of 4.0 mm to ensure a sufficient signal‐to‐noise ratio, rings **(*P*)‐3** and **(*M*)‐3** exhibited a broad band with a peak at 460 nm in the fluorescence spectra (Figure 3b, top) (*Φ* = 0.21). The corresponding CPL spectra of **(*P*)‐3** and **(*M*)‐3** displayed intense negative and positive signals with *g*_lum_ values of −1.8 × 10^−3^ and +1.7 × 10^−3^ at 460 nm, respectively, in the same wavelength region (Figures 3b and S11). Notably, when recorded with a narrower slit width of 0.5 mm, both the fluorescence and CPL spectra showed vibronic structures owing to improved spectral resolution (Figure S12). The absorption spectrum and photoluminescence intensity of the ring ***rac*‐4** in a methanol/chloroform mixture (199:1 v/v) showed no significant changes from those of ***rac*‐4** in pure chloroform (Figure S13). This fact indicates that the optical properties of the *π*‐extended **A7Hel** are maintained even in a highly polar, methanol‐rich environment. After the rotaxane formation, significant changes in the fluorescence and CPL properties occurred (Figure 3b). The fluorescence of *π*‐extended **A7Hel** within rotaxanes **(*P*)‐1** and **(*M*)‐1** was almost completely quenched (*Φ* < 0.01) and thus the CPL signals completely disappeared. This pronounced quenching indicates efficient PET from the *π*‐extended **A7Hel** to PMDI. These results confirm that mechanophores **(*P*)/(*M*)‐1** are in a stable state in which both fluorescence and CPL signals are well quenched under force‐free conditions in solution.

To evaluate mechanoresponsive CPL properties, DN gels ***rac*‐1‐DN‐G**, **(*P*)‐1‐DN‐G**, and **(*M*)‐1‐DN‐G**, which contain mechanophore ***rac*‐1**, **(*P*)‐1**, and **(*M*)‐1** as the crosslinker, respectively, were prepared through a two‐step sequential polymerization (Figure S14). First, a cross‐linked copolymer network was synthesized in an *N*‐methylformamide (NMF) solution containing monomers methoxyethyl methacrylate (MeOEtMA) and potassium salt of 3‐sulfopropyl methacrylate (K‐SPMA), crosslinkers rotaxane mechanophores ***rac*/(*P*)/(*M*)‐1** and poly(ethylene glycol) dimethacrylate (PEGdMA), and a thermal initiator 2,2′‐azobis(4‐methoxy‐2,4‐dimethylvaleronitrile) (V‐70) at room temperature (r.t.). After removal of unreacted species by washing and drying, the first network dried films ***rac*/(*P*)/(*M*)‐1‐SN‐DF** were immersed in the second network precursor solution composed of MeOEtMA, PEGdMA, and a thermal initiator 2,2′‐azobis(isobutyronitrile) (AIBN) in NMF. The presence of the ionic structure derived from K‐SPMA monomer in the first network induced substantial swelling of the ***rac*/(*P*)/(*M*)‐1‐SN‐DF** in the second precursor solution, which effectively pre‐stretched the first network strands. After the second polymerization at 50°C, the second network interpenetrated the first network. After washing and drying the resultant DN gels, the DN dried films ***rac*/(*P*)/(*M*)‐1‐DN‐DF** were obtained. Swelling ***rac*/(*P*)/(*M*)‐1‐DN‐DF** with chloroform or methanol afforded DN gels ***rac*/(*P*)/(*M*)‐1‐DN‐G**.

Chiroptical properties of **(*P*)/(*M*)‐1‐DN‐DF** were investigated to confirm the incorporation of **(*P*)/(*M*)‐1** in the polymer networks (Figure 3c). Both elastomer films **(*P*)/(*M*)‐1‐DN‐DF** showed the CT absorption band analogous to those observed for rotaxanes **(*P*)/(*M*)‐1** in chloroform (Figure 3a, top). Furthermore, mirror‐image CD spectra of rotaxanes **(*P*)/(*M*)‐1** were retained after incorporation into DN elastomers (Figure 3c, bottom). The absorption dissymmetry factors (*g*_abs_) derived from CD spectra of **(*P*)/(*M*)‐1‐DN‐DF** were nearly identical to those of **(*P*)/(*M*)‐1** in chloroform (Figure S15), confirming that both the interlocked architecture of **(*P*)/(*M*)‐1** and the chiroptical properties of *π*‐extended **A7Hel** were preserved throughout the DN gel preparation procedures.

Reference dried films **(*P*)/(*M*)‐3‐DN‐DF** incorporating the polymerizable ring **3** with the same monomer feed ratios were also prepared to evaluate the quenching efficiency within the polymer matrices. While **(*P*)/(*M*)‐3‐DN‐DF** exhibited strong blue fluorescence originating from **A7Hel**, almost no fluorescence of **(*P*)/(*M*)‐1‐DN‐DF** was observed (Figure S16a), which confirms that highly efficient PET from the *π*‐extended **A7Hel** to PMDI is successfully maintained even within the bulk elastomer network. However, the inset in Figure S16a reveals that both dried elastomer films **(*P*)/(*M*)‐1‐DN‐DF** displayed weak blue fluorescence from the *π*‐extended **A7Hel** between 400 and 500 nm. The residual helicene‐derived fluorescence is attributed to a small amount of mechanophores that were activated by the pre‐stretching of the first network induced by swelling in the second monomer solution, and subsequently kinetically trapped in their emissive state. This is supported by the fact that non‐polymerizable rotaxane ***rac*‐2** showed no detectable increase in fluorescence intensity upon the radical polymerization employed for both the first and second network formation (Figure S18b), suggesting that neither chemical degradation of the quencher nor dissociation of the interlocked structure occurred. As the monomer emission intensity is very low, CPL signals from **(*P*)/(*M*)‐1‐DN‐DF** were not clearly detected between 400 and 500 nm (Figure S16b).

**FIGURE 4:**
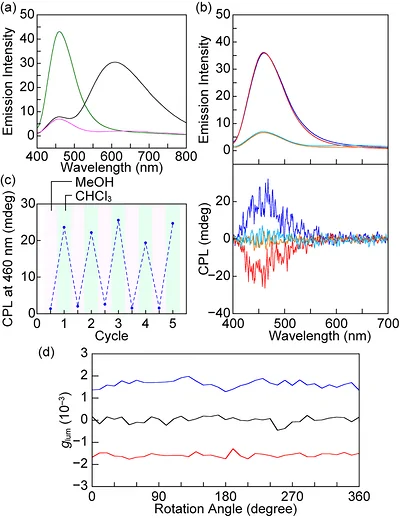
(a) Photoluminescence spectra of ***rac*‐1‐DN‐DF** (black), chloroform‐swollen ***rac*‐1‐DN‐G** (green), and methanol‐swollen ***rac*‐1‐DN‐G** (pink). (b) Photoluminescence (top) and CPL (bottom) spectra of chloroform‐swollen **(*P*)‐1‐DN‐G** (red), chloroform‐swollen **(*M*)‐1‐DN‐G** (blue), methanol‐swollen **(*P*)‐1‐DN‐G** (orange), and methanol‐swollen **(*M*)‐1‐DN‐G** (sky blue). (c) Plots of the CPL intensity at 460 nm of **(*M*)‐1‐DN‐G** upon five‐cycle solvent exchanges between methanol and chloroform. (d) Angular dependence of *g*_lum_ at 460 nm for chloroform‐swollen **(*P*)‐1‐DN‐G** (red), **(*M*)‐1‐DN‐G** (blue), and ***rac*‐1‐DN‐G** (black). *λ*_ex_ = 330 nm.

New broad photoluminescence bands between 500 and 800 nm were also observed in the photoluminescence spectra of **(*P*)/(*M*)‐1‐DN‐DF** (Figures 4a and S16a). The new broad emission bands, which were not detected for **(*P*)/(*M*)‐1** in chloroform, are ascribed to the CT complex formation between **A7Hel** and PMDI. Because the elastomer is less polar than chloroform, non‐radiative decay pathways of the CT excited state were suppressed, thereby promoting radiative decay. Similar behavior has been observed for previously reported cyclophane‐based mechanophores that form the CT complex between emitter and quencher in the force‐free state [85, 86]. When the emission decay profile was recorded for the new broad emission band, no longer emission lifetimes were observed (Figure S17), indicating that the emission is not ascribed to phosphorescence. Notably, almost no CPL signal was detected between 500 and 800 nm (Figure S16b).

The activation of mechanophores was achieved by swelling ***rac*/(*P*)/(*M*)‐1‐DN‐DF** with organic solvents. When immersed in chloroform, which is a good solvent for the polymer network, the volume swelling ratio *Q* of ***rac*/(*P*)/(*M*)‐1‐DN‐G** became 4.5. The chloroform‐swollen ***rac*‐1‐DN‐G** showed an approximately 5.5‐fold increase in blue fluorescence intensity compared to that of ***rac*‐1‐DN‐DF** (Figures 4a, S19b, and S20a). The photoluminescence spectrum of ***rac*‐1‐DN‐G** recorded with narrower slit showed a vibronic structure consistent with that observed for **(*P*)/(*M*)**‐**3** in chloroform (Figures S12a and S20). Moreover, the fluorescence lifetime of the chloroform‐swollen gel was 5.5 ns. Although the fluorescence lifetime of the swollen gel may not be directly comparable to that of the dilute solution due to differences in their local environments, this value is similar to the 5.4 ns lifetime of the reference ***rac*‐3** in chloroform (Figure S22). These results clarified that the blue fluorescence is attributed to the *π*‐extended **A7Hel**. The significant swelling effectively stretched the first network, transmitting sufficient force to the rotaxane mechanophores to separate the ring from the quencher. The pre‐stretching of the first network during the second network formation lowers the strain required to reach the activation threshold, thereby facilitating efficient force transmission. Indeed, a single‐network (SN) dried film ***rac*‐1‐SN‐DF** composed of the first network of the DN gel showed no obvious increase in monomer fluorescence intensity when swollen with chloroform (Figure S20b). The mechanophores that require covalent bond scission for activation have been reported to be activated by swelling the multinetwork gels in which the mechanophore was introduced at the crosslinking point of the first network [68]. Because the activation force of the supramolecular mechanophores is much lower than that of covalent mechanophores, the present supramolecular mechanophores with the helicene emitter are easily activated.

In contrast, when ***rac*‐1‐DN‐DF** was swollen with methanol, almost no increase in monomer fluorescence intensity was observed (Figure 4a), although the *Q* value was 1.9. The retained low fluorescence intensity results not from solvent polarity or aggregation of the helical emitter, but rather from the insufficient deformation of the network for mechanophore activation. As noted above, solvent polarity has little effect on the photophysical properties of the helical emitter (Figure S13). Additionally, a DN gel incorporating the reference ring ***rac*‐3** also maintained strong emission upon swelling with methanol (Figure S21). The CT fluorescence, which was observed for ***rac*‐1‐DN‐DF** film, disappeared after swelling with both chloroform and methanol (Figures 4a and S19c). The quenching of the CT emission is attributed to the solvation of the rotaxanes by polar solvents, which stabilizes the CT excited state and promotes non‐radiative decay pathways. Notably, the solvent‐dependent activation behavior was similarly observed for **(*P*)/(*M*)‐1‐DN‐G** (Figure 4b, top), demonstrating that the force‐induced on/off fluorescence switching property is retained even when the enantiopure helicenes are employed.

Significant enhancements of CPL signals were also achieved by swelling with chloroform (Figure 4b, bottom). The chloroform‐swollen **(*P*)‐1‐DN‐G** and **(*M*)‐1‐DN‐G** exhibited mirror‐image negative and positive CPL signals, respectively, whereas ***rac***‐**1‐DN‐G** was CPL‐inactive (Figure S23b). The CPL signs of **(*P*)/(*M*)‐1‐DN‐G** corresponded to those of the reference rings **(*P*)/(*M*)‐3** in chloroform, confirming that the observed CPL originates from the helicene luminophore and that the mechanically interlocked structure does not significantly affect the excited‐state chiroptical properties of *π*‐extended **A7Hel**. Conversely, consistent with the lack of fluorescence activation for the methanol‐swollen **(*P*)/(*M*)‐1‐DN‐G**, the CPL signals showed no detectable changes between 400 and 500 nm upon swelling with methanol (Figure 4b, bottom). Because the activation of rotaxane mechanophores is inherently reversible, cyclic solvent exchanges reversibly modulate the volume swelling ratio, achieving reversible changes in both monomer fluorescence and CPL signal intensity (Figures 4c and S24). When the methanol‐swollen **(*M*)‐1‐DN‐G** was immersed in chloroform for at least 3 h, the volume of the gel significantly increased, and both fluorescence and CPL signal intensity increased. This indicates that even exchanging the organic solvents in the DN gels transmits sufficient force to activate the rotaxane mechanophores. Conversely, immersing the chloroform‐swollen **(*M*)‐1‐DN‐G** in methanol recovered the initial methanol‐swollen **(*M*)‐1‐DN‐G** and simultaneously both fluorescence and CPL signal intensity decreased. Repeating this sequential solvent exchange five times confirmed the good reversibility of the supramolecular mechanophores **(*P*)/(*M*)‐1**.

Investigation on the angular dependency of the CPL signals of the DN gels revealed that isotropic swelling of gels is one of the most reliable ways to precisely evaluate force‐modulated reciprocal CPL properties. The *g*_lum_ values at 460 nm were plotted against the rotation angle *θ* for the chloroform‐swollen ***rac*/(*P*)/(*M*)‐1‐DN‐G** in an optical cell filled with chloroform (Figure 4d). The *g*_lum_ values of both **(*P*)‐1‐DN‐G** and **(*M*)‐1‐DN‐G** did not show any angular dependence, indicating that the transition dipole moments of *π*‐extended **A7Hel** are randomly oriented in the chloroform‐swollen DN gels. The average *g*_lum_ values were −1.6 × 10^−3^ for **(*P*)‐1‐DN‐G** and +1.7 × 10^−3^ for **(*M*)‐1‐DN‐G**, which are consistent with those recorded for **(*P*)/(*M*)‐3** in chloroform (Figure S11). The *g*_lum_ values of chloroform‐swollen ***rac*‐1‐DN‐G** were nearly zero regardless of *θ*. These results confirmed that the obtained CPL signals from chloroform‐swollen gels are reciprocal and the isotropic swelling of the DN gels properly evaluated reciprocal CPL signals from the emitter, excluding artifacts from linear dichroism and linear birefringence.

**FIGURE 5:**
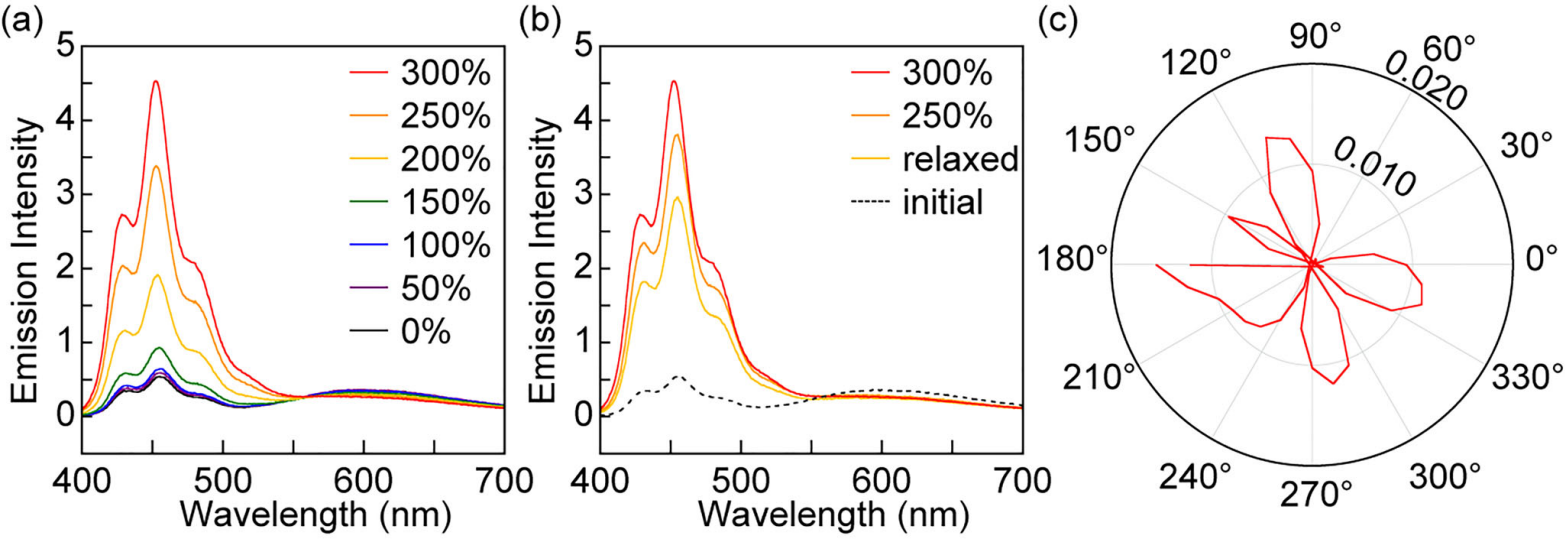
Photoluminescence spectra of a pristine **(*P*)‐1‐DN‐DF** film upon uniaxial (a) deformation and (b) relaxation to the indicated strains. *λ*_ex_ = 365 nm. (c) Polar plots of *g*_lum_ at 460 nm for a **(*P*)‐1‐DN‐DF** film stretched to 260%. *λ*_ex_ = 330 nm.

Uniaxial deformation of mechanophore‐embedded polymer films is not suitable for accurate evaluation of force‐modulated reciprocal CPL. To further support the advantage of the isotropic activation method using DN gels, in situ photoluminescence spectroscopy and CPL measurements were conducted for **(*P*)‐1‐DN‐DF** upon simple uniaxial stretching (Figure 5). The methanol‐swollen DN gels were too brittle to be used for this experiment (Figure S25, Table S3). Uniaxial deformation for the **(*P*)‐1‐DN‐DF** elastomer film gradually activated the mechanophores inside, leading to a significant increase in fluorescence intensity (Figure 5a). Upon relaxation, the fluorescence intensity of the **(*P*)‐1‐DN‐DF** film decreased (Figure 5b). Furthermore, a reference DN dried film in which ***rac*‐1** is physically doped does not show any clear activation upon uniaxial stretching (Figure S26). These results indicate that the uniaxial deformation method is reliable for evaluating changes in photoluminescence intensity as previously demonstrated [87, 88, 89, 90, 91], and force transmission via covalent bonds is vital for the activation of the mechanophore. However, the uniaxial deformation led to strong non‐reciprocal CPL arising from linear dichroism and linear birefringence that prevent accurate measurement of the inherent reciprocal CPL signals. Indeed, the uniaxially deformed **(*P*)‐1‐DN‐DF** film at a strain of 260% showed strong angle‐dependent CPL signals in contrast to the **(*P*)‐1‐DN‐G** isotropically swollen with chloroform (Figures 4d, 5c, and S27). The magnitude of these artifact signals was on the order of 10^−2^, which is approximately 10 times larger than the *g*_lum_ values observed for **(*P*)/(*M*)‐3** in chloroform (Figure S11). Although the *g*_lum_ values can be averaged to eliminate the anisotropy effects, the noise from CPL artifacts still prevents accurate evaluation of the reciprocal CPL from the *π*‐extended **A7Hel**.

## 3 Conclusion

In summary, we demonstrated reciprocal CPL on/off switching by force at the single‐molecule level with a rotaxane architecture. A *π*‐extended aza[7]helicene was chosen as the CPL emitter of the rotaxane mechanophore, and the CPL emission is quenched by PMDI in the force‐free state. Application of force induces spatial separation between the emitter and quencher, activating strong CPL emission. Isotropic swelling of DN gels containing the mechanophores as crosslinkers of the first network was utilized for the mechanophore activation to accurately evaluate reciprocal CPL signals otherwise obscured by artifacts from macroscopic emitter orientation. The degree of mechanophore activation was found to be highly dependent on the swelling ratio, with chloroform inducing sufficient force transmission for CPL activation while methanol did not. Cyclic solvent exchange resulted in reversible changes in both fluorescence and CPL signal intensities because the rotaxane mechanophore does not require covalent bond scission for activation.

Compared to conventional mechanophores that require covalent bond breakage for activation, supramolecular mechanophores offer several advantages, including lower activation energy, good reversibility, force‐specific response, and high design flexibility. In particular, the high design flexibility of rotaxane mechanophores enables precise control of CPL emission, as exemplified by our previous demonstration of force‐controlled FRET efficiency. We are currently exploring supramolecular mechanophores capable of modulating other photofunctions, such as singlet fission and phosphorescence. Furthermore, the isotropic swelling method established here represents a powerful and reliable strategy for the accurate evaluation of single‐molecule‐derived mechanoresponsive CPL. This approach is expected to find broad application in evaluating reciprocal chiroptical properties of diverse mechanoresponsive CPL materials, while avoiding artifacts caused by macroscopic sample orientation.

## Author Contributions

**Keigo Nonaka**: conceptualization, methodology, writing – original draft, writing – review and editing, formal analysis, investigation, software, data curation, validation, funding acquisition, visualization. **Takumi Kuroda**: conceptualization, methodology, data curation, investigation, resources. **Kota Masuda**: methodology, resources. **Toshiki Nishitani**: resources, methodology. **Masaki Enokido**: investigation, methodology, resources, software, data curation, validation. **Makoto Tsurui**: methodology, investigation, resources, data curation, validation. **Yuichi Kitagawa**: methodology, investigation, writing – review and editing, resources, data curation, validation. **Yasuchika Hasegawa**: methodology, investigation, writing – review and editing, resources, data curation, validation. **Keiichi Noguchi**: methodology, data curation, resources, formal analysis. **Koji Nakano**: methodology, data curation, writing – review and editing, project administration, supervision, conceptualization, validation, resources. **Yoshimitsu Sagara**: funding acquisition, writing – original draft, writing – review and editing, project administration, supervision, validation, conceptualization, methodology, resources, visualization.

## Conflicts of Interest

The authors declare no conflicts of interest.

## Supporting information



The authors have cited additional references within the Supporting Information [92, 93, 94, 95]. **Supporting File**: anie73788‐sup‐0001‐SuppMat.pdf.

## Data Availability

The data that support the findings of this study are available in the Supporting Information of this article. Deposition number 2543889 (for **(*P*)‐Ref‐Hel** contains the supplementary crystallographic data for this paper. These data are provided free of charge by the joint Cambridge Crystallographic Data Centre and Fachinformationszentrum Karlsruhe Access Structures service.
