# Supplementary material for: Force‐Induced Control of Circularly Polarized Luminescence With Rotaxane Architecture
Source: Angew Chem Int Ed Engl. 2026 Jul 24;65(39):e9590374. doi: 10.1002/anie.9590374 (PMC13592300; doi:10.1002/anie.9590374)
Supplement: Supplementary file 1 [file ANIE-65-e9590374-s001.pdf]

Supporting Information

Force-induced Control of Circularly Polarized Luminescence with Rotaxane Architecture

Keigo Nonaka, Takumi Kuroda, Kota Masuda, Toshiki Nishitani, Masaki Enokido, Makoto Tsurui, Yuichi Kitagawa, Yasuchika Hasegawa, Keiichi Noguchi, Koji Nakano,* and Yoshimitsu Sagara*

E-mail: k_nakano@cc.tuat.ac.jp; sagara@mct.isct.ac.jp

Table of Contents

Experimental Section ········ **S2**
Synthesis of Rotaxanes and Reference Compounds ········ **S3**
Theoretical Calculations ········ **S13**
Determination of the Absolute Configuration of Helicenes ········ **S14**
NMR Analyses ········ **S17**
Isomeric Analyses of Co-conformational Regioisomers ········ **S19**
Optical Properties of Rings and Rotaxanes in Solutions ········ **S22**
Preparation of DN Dried Films and Gels ········ **S23**
Composition of DN Gels ········ **S25**
Optical Properties of DN Dried Films ········ **S26**
Stability Test of Rotaxane Under Radical Polymerization Conditions ········ **S27**
Optical Properties of DN Gels ········ **S28**
Mechanical Properties of DN Dried Elastomer Films and Gels ········ **S30**
Changes in Photoluminescence Spectra of a DN Dried Film Physically Doped with *rac*-1 upon Stretching ········ **S31**
CPL Measurements of Stretched (*P*)-1-DN-DF ········ **S31**
NMR Spectra of Organic Compounds ········ **S32**
Mass Spectra of Rotaxanes ········ **S52**
DFT-optimized Coordinates ········ **S55**
References ········ **S59**

Experimental Section

General and materials

All reagents and solvents were purchased from FUJIFILM Wako Pure Chemical Corporation, Tokyo Kasei, Kanto Chemical, Merck, or Fluorochem. All reactions were carried out under a nitrogen atmosphere unless otherwise noted.

Flash silica gel column chromatography was conducted with a Biotage Isolera or Select Flash system using SHOKO-scientific Purif-Pack-EX cartridges. Recycling preparative gel permeation chromatography (GPC) was performed with a Japan Analytical Industry LaboACE equipped with a JAIGEL-2HR and 2.5HR column (Japan Analytical Industry, ID = 20 mm, L = 600 mm). Recycling preparative chiral high-performance liquid chromatography (HPLC) was performed with the same Japan Analytical Industry LaboACE instrument equipped with a CHIRALPAK IG column (DAICEL Corporation, ID = 20 mm, L = 250 mm). Analytical chiral HPLC experiments were performed on a SHIMADZU HPLC system equipped with a CHIRALPAK IG column (DAICEL Corporation, ID = 4.6 mm, L = 250 mm). Reversed-phase HPLC was carried out using the same SHIMADZU HPLC system equipped with InertSustain AQ-C18 columns (GL Sciences Inc.). Two types of columns were used: an analytical column (ID = 4.6 mm, L = 250 mm) and a preparative column (ID = 20 mm, L = 250 mm).

^{1}H NMR spectra were measured with a JEOL JNM-ECZ400S spectrometer and all chemical shifts are reported on the *δ*-scale in ppm relative to the signal of tetramethylsilane (TMS at 0.00) or residual solvent protons ($CHCl_3$ at 7.26) as the internal standard. Proton-decoupled ^{13}C NMR spectra were acquired on the same JEOL JNM ECZ400S spectrometer and all chemical shifts were expressed in ppm using solvent as the internal standard ($CDCl_3$ at 77.16). Coupling constants (*J*) were quoted in Hz and relative intensities were reported. Matrix Assisted Laser Desorption Ionization Time-of-Flight (MALDI-TOF) mass spectrometry was performed with a SHIMADZU MALDI-8030. High-resolution electrospray ionization (ESI) mass spectra were measured with a Bruker Daltonics micrOTOF II.

The following reagents were used for the preparation of DN gels. Methoxyethyl methacrylate (MeOEtMA), 3-sulfopropyl methacrylate potassium salt (K-SPMA), and poly(ethylene glycol) dimethacrylate (PEGdMA, n ≈ 14) were purified by passing through activated basic alumina to remove inhibitors before use. 2,2'-Azobis(4-methoxy-2,4-dimethylvaleronitrile) (V-70), 2,2'-azobis(isobutyronitrile) (AIBN), and *N*-methylformamide (NMF) were used as received. Gel preparation was conducted in a UNICO UN-800L-TKS glovebox. A digital hot plate NINOS ND-2A was used as a heat source of second network polymerization.

UV-vis absorption spectra were recorded on a JASCO V-750 or JASCO J-1100. Molar extinction coefficients (*ε*) were calculated according to the Beer–Lambert law using absorbance values measured at specific concentrations in a quartz cuvette with a 1 cm path length. CD spectra were recorded on a JASCO J-1100. Time-resolved PL measurements were carried out with a Hamamatsu Photonics Quantaurus-Tau. Absolute photoluminescence quantum yields were measured with a Hamamatsu Photonics Quantaurus-QY. CPL spectra were recorded on a JASCO CPL-300. Steady-state PL spectra of single-network (SN) dried films, SN gels, double-network (DN) dried films, and DN gels were monitored with a JASCO FP-8550, a JASCO CPL-300, or an Ocean Optics QEPro-FL equipped with a Reflection/Backscattering Probe R400-7-UV-VIS. The excitation light at 365 nm was obtained using an Ocean Insight LLS-365 LED light source. Stress-strain measurements were conducted under ambient conditions with a SHIMADZU AGS-100NX equipped with a 100 N load cell at a strain rate of 120 mm min^{-1}. Photographs were taken with a Canon EOS 9000D stabilized with a tripod.

All quantum chemical calculations were performed using the GAUSSIAN 16 revision C01 program.[S1] The initial structures were prepared using MoleditPy program (version 1.18.1).[S2] Geometry optimizations and HOMO–LUMO energy level calculations were carried out using density functional theory (DFT) at M06-2X/def2-TZVP level of theory.

Synthesis of Rotaxanes and Reference Compounds

The synthetic route used to prepare ***rac*-4** is shown in Scheme S1. [4,4′-Biphenanthrene]-3,3′-diol, compound **9**, and compound **10** were prepared according to reported procedures.[S3–S5] The synthesis of the reference ring **3**, rotaxane **2**, and rotaxane **1**, following the enantioseparation of ***rac*-4**, is shown in Scheme S2. Compounds **11** and **12** were prepared according to reported procedures.[S6, S7] The syntheses of ***rac*-Ref-Hel** and compound **13** are shown in Schemes S3 and S4, respectively.

Scheme S1. Synthesis of ***rac*-4**.

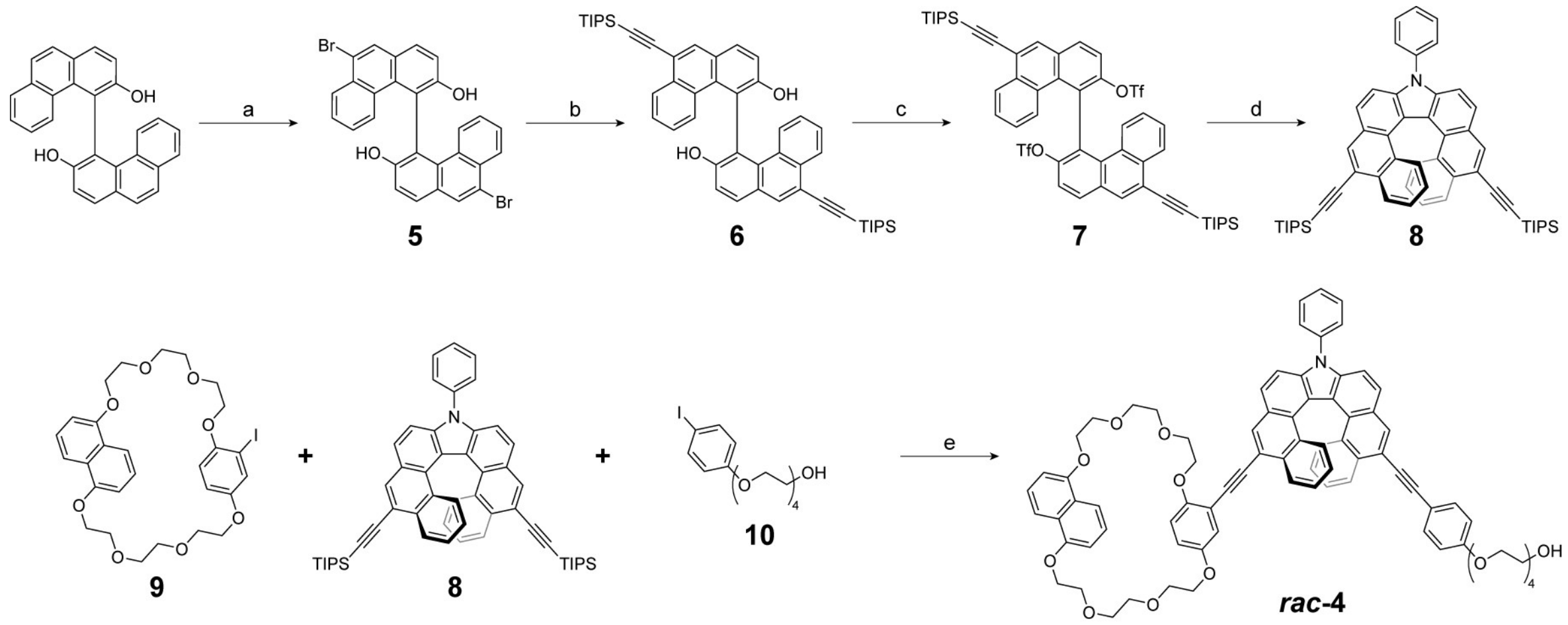

Conditions: (a) Br_2, CH_2Cl_2, r.t., 2 h; (b) triisopropylsilylacetylene, $Pd(PPh_3)_4$, CuI, THF, Et_3N, reflux, 120 h; (c) trifluoromethanesulfonic anhydride, pyridine, 0 °C → r.t., 2 h; (d) aniline, tris(dibenzylideneacetone)dipalladium(0), 4,5-bis(diphenylphosphino)-9,9-dimethylxanthene, K_3PO_4, toluene, 100 °C, 137 h; (e) i) tetrabutylammonium fluoride, Et_3N, THF, r.t., 15 min, ii) $Pd(PPh_3)_4$, CuI, Et_3N, THF, under N_2/H_2 atmosphere, 60 °C, 14 h.

Compound 5. To a mixture of [4,4′-biphenanthrene]-3,3′-diol (827 mg, 2.14 mmol), and dichloromethane (20 mL), a dichloromethane solution of Br_2 (ca. 1.56 mol/L, 3.29 mL, 5.14 mmol) was added and stirred at r.t. for 2 h. The reaction was quenched by adding saturated aq. $Na_2S_2O_3$ (20 mL), and the reaction mixture was poured into the mixture of ethyl acetate (150 mL) and acetone (10 mL) and washed with saturated aq. $Na_2S_2O_3$ (3 × 150 mL). The organic layer was separated, dried over $MgSO_4$, filtered, and the solvent was evaporated under reduced pressure. The crude product was purified by flash column chromatography on silica gel (eluent: gradient from hexane/ethyl acetate = 19:1 v/v to hexane/ethyl acetate = 4:1 v/v) to afford compound **5** (970 mg, 1.78 mmol, 84%) as a white solid.
^{1}H NMR (400 MHz, $CDCl_3$): δ = 8.37–8.35 (m, 2H), 8.22 (s, 2H), 8.10 (d, J = 8.4 Hz, 2H), 8.00 (d, J = 8.8 Hz, 2H), 7.52 (ddd, J = 8.4, 7.2, 1.2 Hz, 2H), 7.44 (d, J = 8.8 Hz, 2H), 7.02 (ddd, J = 8.4, 7.2, 1.2 Hz, 2H), 5.00 (s, 2H).
^{13}C NMR (100 MHz, $CDCl_3$): δ = 153.25, 131.94, 131.81, 131.32, 131.10, 129.31, 129.20, 128.37, 128.15, 127.37, 125.02, 120.69, 118.55, 116.39.
MS (MALDI-TOF): m/z: 542.79 (calcd. $[M+H]^+$ = 542.96).

Compound 6. To a mixture of compound **5** (970 mg, 1.78 mmol), triisopropylsilylacetylene (1.95 g, 10.7 mmol), Et_3N (20 mL), and THF (20 mL), $Pd(PPh_3)_4$ (16 mg, 1.5×10^{-2} mmol) and CuI (5.0 mg, 3.0×10^{-2} mmol) were added and the reaction mixture was stirred under reflux for 120 h. After cooling to r.t., the reaction mixture was poured into ethyl acetate (200 mL) and washed with 5% aq. HCl (150 mL), saturated aq. $NaHCO_3$ (150 mL), and saturated aq. NaCl (150 mL). The organic layer was separated, dried over $MgSO_4$, filtered, and the solvent was evaporated under reduced pressure. The crude product was purified by flash column chromatography on silica gel (eluent: gradient from hexane/ethyl acetate = 9:1 v/v to hexane/ethyl acetate = 7:3 v/v) to afford compound **6** (732 mg, 0.979 mmol,

55%) as a yellow solid.
^{1}H NMR (400 MHz, $CDCl_3$): δ = 8.51–8.49 (m, 2H), 8.15 (s, 2H), 8.09–8.04 (m, 4H), 7.49 (ddd, J = 8.0, 7.2, 0.8 Hz, 2H), 7.43 (d, J = 8.8 Hz, 2H), 6.98 (ddd, J = 8.4, 7.2, 1.2 Hz, 2H), 5.01 (s, 2H), 1.27–1.21 (m, 42H).
^{13}C NMR (100 MHz, $CDCl_3$): δ = 153.68, 133.25, 132.93, 132.52, 129.92, 129.84, 128.18, 127.64, 127.25, 126.89, 125.02, 119.04, 118.21, 116.53, 104.99, 95.98, 18.95, 11.57.
MS (MALDI-TOF): m/z: 747.73 (calcd. $[M+H]^+$ = 747.40).

Compound 7. To a mixture of compound **6** (1.85 g, 2.48 mmol), pyridine (600 µL, 7.43 mmol) in dichloromethane (40 mL), trifluoromethanesulfonic anhydride (977 µL, 5.95 mmol) was added at 0 °C, and stirred at r.t. for 2 h. The reaction mixture was poured into dichloromethane (200 mL) and washed with 5% aq. HCl (150 mL), saturated aq. $NaHCO_3$ (150 mL), and saturated aq. NaCl (150 mL). The organic layer was separated, dried over $MgSO_4$, filtered, and the solvent was evaporated under reduced pressure. The crude product was purified by flash column chromatography on silica gel (eluent: gradient from hexane/ethyl acetate = 4:1 v/v to hexane/ethyl acetate = 7:3 v/v) to afford compound **7** (1.89 g, 1.87 mmol, 75%) as a yellow solid.
^{1}H NMR (400 MHz, $CDCl_3$): δ = 8.61–8.58 (m, 2H), 8.23 (s, 2H), 8.19 (d, J = 9.2 Hz, 2H), 7.94 (d, J = 8.4 Hz, 2H), 7.70 (d, J = 8.8 Hz, 2H), 7.58 (ddd, J = 7.2, 6.4, 0.8 Hz, 2H), 7.05 (ddd, J = 8.8, 7.2, 1.6 Hz, 2H), 1.31–1.23 (m, 42H).
^{13}C NMR (100 MHz, $CDCl_3$): δ = 146.20, 132.89, 132.22, 132.18, 132.03, 130.83, 130.11, 128.15, 127.66, 127.32, 127.07, 125.56, 122.70, 120.24, 117.99 (q, J = 319 Hz, CF_3), 104.26, 97.92, 18.94, 11.55.
MS (MALDI-TOF): m/z: 1033.16 (calcd. $[M+Na]^+$ = 1033.28).

Compound 8. K_3PO_4 (495 mg, 2.33 mmol) was dried at 203 °C for 12 h. After cooling to r.t., compound **7** (786 mg, 0.777 mmol), aniline (85.0 µL, 0.930 mmol), 4,5-bis(diphenylphosphino)-9,9-dimethylxanthene (45.0 mg, 7.80×10^{-2} mmol), tris(dibenzylideneacetone)dipalladium(0) (35 mg, 3.9×10^{-2} mmol), and toluene (10 mL) were added and stirred at 100 °C for 137 h. The reaction mixture was poured into chloroform (200 mL) and washed with 5% aq. HCl (150 mL), saturated aq. $NaHCO_3$ (150 mL), and saturated aq. NaCl (150 mL). The organic layer was separated, dried over $MgSO_4$, filtered, and the solvent was evaporated under reduced pressure. The crude product was purified by flash column chromatography on silica gel (eluent: hexane/dichloromethane = 17:3 v/v) to afford compound **8** (447 mg, 0.556 mmol, 72%) as a yellow solid.
^{1}H NMR (400 MHz, $CDCl_3$): δ = 8.44–8.42 (m, 2H), 8.31 (s, 2H), 8.00 (d, J = 8.8 Hz, 2H), 7.76 (d, J = 8.8 Hz, 2H), 7.74–7.72 (m, 4H), 7.68–7.62 (m, 1H), 7.44 (d, J = 8.0 Hz, 2H), 7.31 (ddd, J = 8.0, 7.2, 0.8 Hz, 2H), 6.33 (ddd, J = 8.0, 6.8, 1.2 Hz, 2H), 1.31–1.24 (m, 42H).
^{13}C NMR (100 MHz, $CDCl_3$): δ = 140.91, 136.88, 132.78, 130.69, 130.32, 130.12, 128.95, 128.72, 128.26, 127.29, 127.17, 126.83, 126.82, 124.91, 123.43, 117.37, 117.12, 111.33, 105.83, 94.79, 19.04, 11.67.
MS (MALDI-TOF): m/z: 804.86 (calcd. $[M+H]^+$ = 804.44).

***rac*-4.** To a mixture of compound **9** (179 mg, 0.286 mmol), compound **8** (230 mg, 0.286 mmol), compound **10** (113 mg, 0.286 mmol), and triethylamine (20 mL) in THF (20 mL), a THF solution of tetrabutylammonium fluoride (ca. 1 mol/L, 0.72 mL, 0.72 mmol) was added and stirred at r.t. for 15 min. Then, $Pd(PPh_3)_4$ (48 mg, 4.2×10^{-2} mmol) and CuI (8.0 mg, 4.2×10^{-2} mmol) were added and the reaction mixture was stirred under N_2/H_2 atmosphere at 60 °C for 14 h. After cooling to r.t., the reaction mixture was poured into chloroform (150 mL) and washed with 5% aq. HCl (150 mL), saturated aq. $NaHCO_3$ (150 mL), and saturated aq. NaCl (150 mL). The organic layer was separated, dried over $MgSO_4$, filtered, and the solvent was evaporated under reduced pressure. The crude product was purified by flash column chromatography on silica gel (eluent: gradient from dichloromethane to dichloromethane/acetone = 3:1 v/v), and recycling GPC (eluent: chloroform) to afford ***rac*-4** (125 mg, 9.94×10^{-2} mmol, 35%) as a yellow solid.
^{1}H NMR (400 MHz, $CDCl_3$): δ = 8.57 (d, J = 8.0 Hz, 1H), 8.47 (d, J = 8.0 Hz, 1H), 8.32 (s, 1H), 8.29 (s, 1H), 8.00

(d, J = 8.4 Hz, 2H), 7.91 (t, J = 8.8 Hz, 2H), 7.77 (d, J = 8.8 Hz, 2H), 7.73–7.71 (m, 4H), 7.68–7.62 (m, 3H), 7.48 (d, J = 8.4 Hz, 2H), 7.32–7.28 (m, 4H), 6.99–6.96 (m, 3H), 6.83 (t, J = 8.8 Hz, 2H), 6.37–6.30 (m, 3H), 6.24 (dd, J = 8.8, 3.2 Hz, 1H), 4.33–4.31 (m, 2H), 4.30–4.28 (m, 2H), 4.20 (t, J = 4.4 Hz, 2H), 4.03–3.97 (m, 8H), 3.90 (t, J = 4.4 Hz, 2H), 3.86–3.68 (m, 22H), 3.64–3.62 (m, 2H), 2.79 (br, 1H).
^{13}C NMR (100 MHz, $CDCl_3$): δ = 158.88, 154.49, 154.42, 154.01, 152.67, 140.81, 136.90, 133.23, 131.50, 131.33, 130.67, 130.53, 130.31, 130.19, 130.13, 128.91, 128.64, 128.29, 128.16, 127.16, 127.13, 127.10, 126.93, 126.89, 126.73, 125.33, 125.24, 124.80, 123.53, 123.46, 119.32, 117.53, 117.28, 117.17, 116.17, 116.12, 114.87, 114.79, 114.61, 114.30, 113.87, 111.32, 105.82, 105.74, 93.65, 92.21, 90.52, 87.16, 72.70, 71.47, 71.44, 71.26, 71.00, 70.95, 70.78, 70.69, 70.40, 70.05, 69.91, 69.79, 69.27, 68.46, 67.88, 67.77, 67.57, 61.88.
HRMS (ESI-TOF): m/z: 1278.4960 (calcd. $[M+Na]^+$ = 1278.4974).

Enantioseparation of *rac*-4

Compound ***rac*-4** (110 mg) was subjected to recycle chiral HPLC (eluent: hexane/dichloromethane/ethanol = 47:47:6 v/v/v). The separated fractions were collected and concentrated to yield **(*P*)-4** (54 mg, first fraction) and **(*M*)-4** (52 mg, second fraction).

(*P*)-4.

^{1}H NMR (400 MHz, $CDCl_3$): δ = 8.57 (d, J = 8.4 Hz, 1H), 8.47 (d, J = 8.0 Hz, 1H), 8.32 (s, 1H), 8.29 (s, 1H), 8.00 (d, J = 8.8 Hz, 2H), 7.91 (t, J = 8.8 Hz, 2H), 7.77 (d, J = 8.4 Hz, 2H), 7.73–7.71 (m, 4H), 7.67–7.62 (m, 3H), 7.48 (d, J = 8.4 Hz, 2H), 7.32–7.28 (m, 4H), 7.00–6.96 (m, 3H), 6.83 (t, J = 8.8 Hz, 2H), 6.37–6.31 (m, 3H), 6.24 (dd, J = 8.8, 2.8 Hz, 1H), 4.34–4.32 (m, 2H), 4.30–4.28 (m, 2H), 4.21 (t, J = 4.4 Hz, 2H), 4.04–3.97 (m, 8H), 3.90 (t, J = 4.4 Hz, 2H), 3.86–3.70 (m, 22H), 3.64–3.62 (m, 2H), 2.72 (br, 1H).
^{13}C NMR (100 MHz, $CDCl_3$): δ = 158.90, 154.50, 154.43, 154.02, 152.68, 140.82, 136.91, 133.24, 131.50, 131.34, 130.68, 130.54, 130.32, 130.20, 130.14, 128.92, 128.65, 128.29, 128.17, 127.17, 127.13, 127.11, 126.94, 126.90, 126.73, 125.33, 125.26, 124.80, 123.53, 123.46, 119.32, 117.55, 117.30, 117.19, 116.18, 116.13, 114.88, 114.80, 114.62, 114.31, 113.88, 111.32, 111.31, 105.83, 105.76, 93.66, 92.21, 90.52, 87.16, 72.68, 71.48, 71.45, 71.27, 71.02, 70.97, 70.79, 70.71, 70.44, 70.06, 69.92, 69.81, 69.29, 68.47, 67.90, 67.79, 67.59, 61.90.
HRMS (ESI-TOF): m/z: 1278.4967 (calcd. $[M+Na]^+$ = 1278.4974).

(*M*)-4.

^{1}H NMR (400 MHz, $CDCl_3$): δ = 8.57 (d, J = 8.0 Hz, 1H), 8.47 (d, J = 8.0 Hz, 1H), 8.33 (s, 1H), 8.30 (s, 1H), 8.01 (d, J = 8.8 Hz, 2H), 7.91 (t, J = 8.8 Hz, 2H), 7.78 (d, J = 8.8 Hz, 2H), 7.75–7.72 (m, 4H), 7.69–7.63 (m, 3H), 7.48 (d, J = 8.4 Hz, 2H), 7.32–7.27 (m, 4H), 7.00–6.96 (m, 3H), 6.86–6.81 (m, 2H), 6.37–6.31 (m, 3H), 6.24 (dd, J = 8.8, 3.2 Hz, 1H), 4.34–4.32 (m, 2H), 4.31–4.29 (m, 2H), 4.22–4.20 (m, 2H), 4.04–3.96 (m, 8H), 3.91 (t, J = 4.8 Hz, 2H), 3.86–3.70 (m, 22H), 3.64–3.62 (m, 2H), 2.88 (br, 1H).
^{13}C NMR (100 MHz, $CDCl_3$): δ = 158.88, 154.49, 154.42, 154.01, 152.67, 140.80, 136.89, 133.23, 131.50, 131.33, 130.67, 130.53, 130.31, 130.19, 130.13, 128.91, 128.63, 128.29, 128.16, 127.16, 127.13, 127.09, 126.93, 126.89, 126.73, 125.33, 125.24, 124.80, 123.52, 123.46, 119.31, 117.53, 117.29, 117.17, 116.17, 116.12, 114.87, 114.79, 114.61, 114.30, 113.87, 111.30, 105.82, 105.75, 93.65, 92.21, 90.52, 87.16, 72.69, 71.46, 71.44, 71.26, 71.00, 70.95, 70.77, 70.69, 70.41, 70.05, 69.91, 69.79, 69.27, 68.46, 67.88, 67.77, 67.57, 61.88.
HRMS (ESI-TOF): m/z: 1278.4962 (calcd. $[M+Na]^+$ = 1278.4974).

Scheme S2. Synthesis of ***rac*/(*P*)/(*M*)-1**.

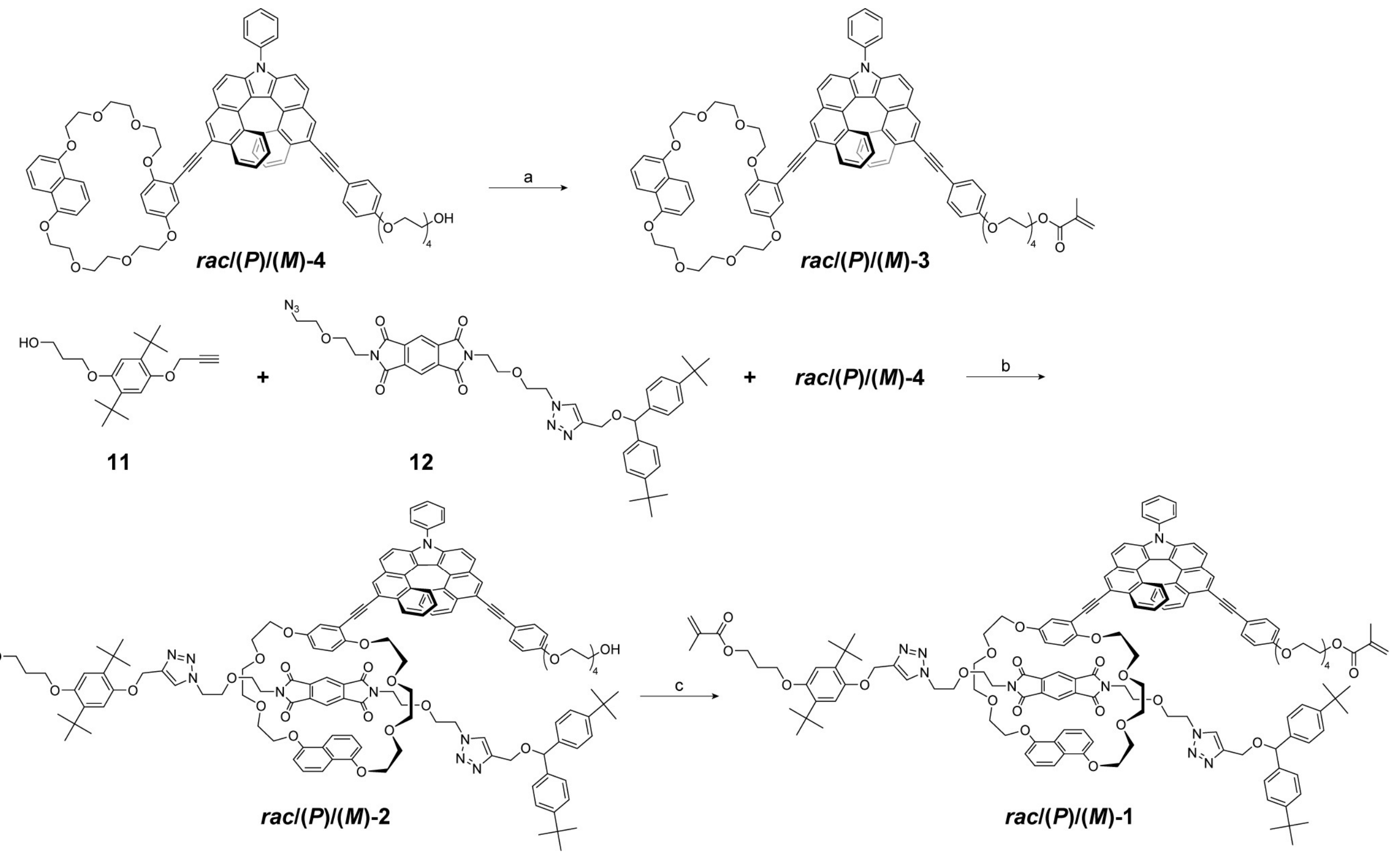

Conditions: (a) methacryloyl chloride, CH_2Cl_2, Et_3N, 0 ºC → r.t., 20 h; (b) $CuSO_4$, sodium ascorbate, $CHCl_3$, H_2O, 5 °C, 14 h; (c) methacryloyl chloride, CH_2Cl_2, Et_3N, 0 ºC → r.t., 20 h.

***rac*-3.** To a mixture of ***rac*-4** (15 mg, 1.2 × 10^{-2} mmol), distillated triethylamine (62 µL, 4.5 × 10^{-1} mmol), and dichloromethane (10 mL), methacryloyl chloride (14 µL, 1.5 × 10^{-1} mmol) were added at 0 °C and the reaction mixture was stirred at r.t. for 20 h. The reaction was quenched by adding methanol (5 mL) at 0 °C, and the reaction mixture was poured into dichloromethane (200 mL) and washed with water (150 mL), and saturated aq. NaCl (150 mL). The organic layer was separated, dried over $MgSO_4$, filtered, and the solvent was evaporated under reduced pressure. The crude product was purified by flash column chromatography on silica gel (eluent: gradient from dichloromethane to dichloromethane/acetone = 17:3 v/v) to afford ***rac*-3** (7.6 mg, 5.7× 10^{-3} mmol, 48%) as a yellow solid.

^{1}H NMR (400 MHz, $CDCl_3$): δ = 8.57 (d, J = 8.0 Hz, 1H), 8.47 (d, J = 7.6 Hz, 1H), 8.33 (s, 1H), 8.30 (s, 1H), 8.03–8.01 (m, 2H), 7.91 (t, J = 8.8 Hz, 2H), 7.79–7.77 (m, 2H), 7.75–7.73 (m, 4H), 7.68–7.63 (m, 3H), 7.48 (d, J = 8.4 Hz, 2H), 7.32–7.27 (m, 4H), 7.00–6.96 (m, 3H), 6.86–6.82 (m, 2H), 6.37–6.31 (m, 3H), 6.25 (dd, J = 8.8, 2.8 Hz, 1H), 6.15–6.14 (m, 1H), 5.59–5.58 (m, 1H), 4.35–4.29 (m, 6H), 4.22–4.19 (m, 2H), 4.05–3.96 (m, 8H), 3.91 (t, J = 4.8 Hz, 2H), 3.87–3.67 (m, 22H), 1.96–1.96 (m, 3H).

^{13}C NMR (100 MHz, $CDCl_3$): δ = 167.55, 158.96, 154.51, 154.44, 154.03, 152.69, 140.84, 136.94, 136.28, 133.24, 131.51, 131.35, 130.68, 130.55, 130.34, 130.21, 130.14, 128.94, 128.68, 128.30, 128.18, 127.19, 127.13, 126.96, 126.91, 126.74, 125.97, 125.35, 125.26, 124.81, 123.55, 123.47, 119.33, 117.55, 117.31, 117.20, 116.15, 114.88, 114.81, 114.63, 114.33, 113.89, 111.34, 111.32, 105.84, 105.76, 93.65, 92.22, 90.52, 87.16, 71.51, 71.47, 71.28, 71.03, 70.85, 70.81, 70.07, 69.94, 69.84, 69.30, 68.48, 67.91, 67.80, 67.63, 64.04, 18.51.

HRMS (ESI-TOF): m/z: 1346.5224 (calcd. $[M+Na]^+$ = 1346.5236).

The enantiomers **(*P*)-3** and **(*M*)-3** were synthesized according to the same procedure used for ***rac*-3**. The enantiomer **(*P*)-3** was obtained as a yellow solid (25 mg, 1.9 × 10^{-2} mmol, 89%) from **(*P*)-4** (27 mg, 2.2 × 10^{-2} mmol). The

enantiomer **(*M*)-3** was obtained as a yellow solid (3 mg, 2 × 10^{-3} mmol, 19%) from **(*M*)-4** (15 mg, 1.2 × 10^{-2} mmol).

(*P*)-3.

^{1}H NMR (400 MHz, $CDCl_3$): *δ* = 8.57 (d, *J* = 8.0 Hz, 1H), 8.47 (d, *J* = 8.0 Hz, 1H), 8.33 (s, 1H), 8.30 (s, 1H), 8.02–8.00 (m, 2H), 7.91 (t, *J* = 8.8 Hz, 2H), 7.78–7.76 (m, 2H), 7.74–7.73 (m, 4H), 7.68–7.65 (m, 3H), 7.48 (d, *J* = 8.4 Hz, 2H), 7.32–7.26 (m, 4H), 6.99–6.96 (m, 3H), 6.85–6.81 (m, 2H), 6.36–6.31 (m, 3H), 6.24 (dd, *J* = 8.8, 2.8 Hz, 1H), 6.15 (s, 1H), 5.59–5.58 (m, 1H), 4.33–4.30 (m, 6H), 4.21–4.19 (m, 2H), 4.04–3.98 (m, 8H), 3.90 (t, *J* = 4.8 Hz, 2H), 3.86–3.62 (m, 22H), 1.96 (s, 3H).

^{13}C NMR (100 MHz, $CDCl_3$): *δ* = 167.53, 158.94, 154.48, 154.42, 154.01, 152.67, 140.81, 136.90, 136.25, 133.22, 131.50, 131.34, 130.67, 130.53, 130.31, 130.19, 130.12, 128.92, 128.64, 128.29, 128.16, 127.16, 127.13, 127.10, 126.94, 126.89, 126.72, 125.96, 125.33, 125.24, 124.80, 123.53, 123.45, 119.32, 117.53, 117.28, 117.17, 116.13, 114.87, 114.79, 114.61, 114.31, 113.87, 111.31, 105.82, 105.75, 93.65, 92.20, 90.51, 87.15, 71.47, 71.44, 71.25, 71.00, 70.78, 70.05, 69.91, 69.81, 69.28, 68.46, 67.88, 67.77, 67.60, 64.02, 18.49.

HRMS (ESI-TOF): m/z: 1346.5218 (calcd. $[M+Na]^+$ = 1346.5236).

(*M*)-3.

^{1}H NMR (400 MHz, $CDCl_3$): *δ* = 8.57 (d, *J* = 8.0 Hz, 1H), 8.47 (d, *J* = 8.0 Hz, 1H), 8.33 (s, 1H), 8.30 (s, 1H), 8.03–8.01 (m, 2H), 7.91 (t, *J* = 8.8 Hz, 2H), 7.80–7.77 (m, 2H), 7.76–7.73 (m, 4H), 7.69–7.64 (m, 3H), 7.48 (d, *J* = 8.4 Hz, 2H), 7.32–7.27 (m, 4H), 7.00–6.96 (m, 3H), 6.86–6.82 (m, 2H), 6.37–6.31 (m, 3H), 6.25 (dd, *J* = 8.8, 2.8 Hz, 1H), 6.15 (s, 1H), 5.59–5.58 (m, 1H), 4.35–4.29 (m, 6H), 4.22–4.20 (m, 2H), 4.05–3.96 (m, 8H), 3.91 (t, *J* = 4.8 Hz, 2H), 3.87–3.65 (m, 22H), 1.96 (s, 3H).

^{13}C NMR (100 MHz, $CDCl_3$): *δ* = 167.53, 158.94, 154.49, 154.43, 154.02, 152.68, 140.81, 136.91, 136.26, 133.23, 131.50, 131.34, 130.67, 130.53, 130.32, 130.20, 130.13, 128.93, 128.65, 128.29, 128.17, 127.17, 127.13, 127.11, 126.94, 126.89, 126.73, 125.97, 125.34, 125.25, 124.80, 123.53, 123.46, 119.32, 117.54, 117.29, 117.18, 116.13, 114.87, 114.79, 114.61, 114.31, 113.87, 111.33, 111.31, 105.82, 105.74, 93.65, 92.21, 90.52, 87.15, 71.48, 71.45, 71.26, 71.01, 70.82, 70.79, 70.06, 69.92, 69.82, 69.29, 68.46, 67.89, 67.77, 67.61, 64.03, 18.50.

HRMS (ESI-TOF): m/z: 1346.5211 (calcd. $[M+Na]^+$ = 1346.5236).

***rac*-2.** A mixture of sodium ascorbate (21 mg, 0.11 mmol) and copper(II) sulfate (5.1 mg, 3.2 × 10^{-2} mmol) in water (1 mL) was added to a solution of compound **11** (38 mg, 0.12 mmol), compound **12** (93 mg, 0.12 mmol), and ring ***rac*-4** (150 mg, 0.119 mmol) in chloroform (1 mL) and the mixture was vigorously stirred at 5 °C for 14 h. The suspension was poured into a mixture of water (100 mL) and chloroform (150 mL) and washed with saturated aq. NaCl (100 mL). The organic layer was separated, dried over $MgSO_4$, filtered, and the solvent was evaporated under reduced pressure. The crude product was purified by flash column chromatography on silica gel (eluent: gradient from dichloromethane/acetone = 17:3 v/v to dichloromethane/acetone = 1:1 v/v), recycling GPC (eluent: chloroform), and reversed-phase HPLC (eluent: gradient from methanol/dichloromethane = 9:1 v/v to methanol/dichloromethane = 4:1 v/v) to afford ***rac*-2** (31 mg, 1.3 × 10^{-2} mmol, 11%) as an orange solid.

^{1}H NMR (400 MHz, $CDCl_3$): *δ* = 8.53–8.50 (m, 1H), 8.43 (d, *J* = 8.0 Hz, 1H), 8.35–8.34 (m, 1H), 8.23–8.21 (m, 1H), 8.07–7.98 (m, 4H), 7.82–7.17 (m, 25H), 7.02–6.99 (m, 2H), 6.97–6.86 (m, 1H), 6.85–6.74 (m, 3H), 6.60–6.31 (m, 5H), 6.19–6.14 (m, 1H), 6.05–6.01 (m, 1H), 5.42–5.29 (m, 1H), 5.19–4.88 (m, 2H), 4.58–3.28 (m, 62H), 2.99–2.96 (m, 1H), 2.12–2.04 (m, 2H), 1.84–1.78 (m, 1H), 1.37–1.29 (m, 18H), 1.26–1.24 (m, 18H).

^{13}C NMR (100 MHz, $CDCl_3$): *δ* = 166.79, 166.75, 158.90, 153.91, 153.79, 153.01, 152.99, 151.81, 151.79, 151.51, 151.45, 150.51, 150.25, 150.20, 145.58, 145.52, 145.48, 145.44, 145.34, 145.05, 145.01, 144.90, 144.79, 140.93, 140.89, 140.81, 138.83, 136.68, 136.46, 136.22, 134.36, 134.30, 134.26, 134.22, 134.15, 133.20, 131.70, 131.58, 130.53, 130.36, 130.07, 130.04, 129.01, 128.62, 128.21, 128.17, 128.09, 127.35, 127.17, 127.04, 127.01, 126.93, 126.82, 126.68, 126.61, 125.78, 125.73, 125.60, 125.57, 125.33, 124.86, 124.25, 124.22, 124.19, 124.11, 123.74, 123.65, 123.60, 123.55, 123.50, 123.34, 118.27, 118.23, 117.36, 117.18, 117.06, 116.93, 116.89, 116.70, 116.08,

115.46, 115.42, 114.85, 114.35, 114.22, 114.05, 112.88, 112.82, 112.76, 112.68, 112.56, 112.00, 111.50, 111.22, 105.29, 93.78, 92.83, 92.75, 89.49, 89.43, 87.07, 82.80, 82.75, 72.63, 71.09, 70.90, 70.73, 70.65, 70.36, 70.00, 69.97, 69.85, 69.75, 69.48, 69.43, 68.68, 68.59, 68.57, 68.53, 68.42, 68.28, 68.15, 68.05, 67.93, 67.80, 67.74, 67.55, 65.74, 63.09, 62.94, 62.81, 62.37, 62.31, 62.26, 62.11, 61.80, 60.43, 50.39, 50.27, 50.21, 50.13, 50.05, 36.88, 36.80, 36.70, 34.71, 34.64, 34.51, 32.65, 31.41, 29.99.

HRMS (ESI-TOF): m/z: 1198.0344 (calcd. $[M+2Na]^{2+}$ = 1198.0354).

The rotaxanes **(*P*)-2** and **(*M*)-2** were synthesized according to the same procedure used for ***rac*-2**. The rotaxane **(*P*)-2** was obtained as an orange solid (24 mg, 1.0 × 10^{-2} mmol, 9%) from **(*P*)-4** (150 mg, 0.119 mmol). The rotaxane **(*M*)-2** was obtained as an orange solid (38 mg, 1.6 × 10^{-2} mmol, 14%) from **(*M*)-4** (143 mg, 0.114 mmol).

(*P*)-2.

^{1}H NMR (400 MHz, $CDCl_3$): δ = 8.54–8.50 (m, 1H), 8.43 (d, J = 8.0 Hz, 1H), 8.35–8.34 (m, 1H), 8.22–8.20 (m, 1H), 8.06–7.97 (m, 4H), 7.81–7.17 (m, 25H), 7.02–6.99 (m, 2H), 6.97–6.86 (m, 1H), 6.85–6.74 (m, 3H), 6.61–6.31 (m, 5H), 6.19–6.14 (m, 1H), 6.05–6.01 (m, 1H), 5.42–5.29 (m, 1H), 5.19–4.88 (m, 2H), 4.58–3.27 (m, 62H), 2.89 (br, 1H), 2.12–2.05 (m, 2H), 1.85 (br, 1H), 1.37–1.29 (m, 18H), 1.25–1.24 (m, 18H).

^{13}C NMR (100 MHz, $CDCl_3$): δ = 166.86, 166.81, 158.91, 153.97, 153.94, 153.82, 153.04, 153.03, 151.86, 151.83, 151.54, 151.50, 151.47, 150.55, 150.50, 150.28, 150.24, 145.63, 145.57, 145.49, 145.39, 145.10, 145.05, 144.95, 144.83, 140.97, 140.93, 140.85, 138.89, 138.85, 136.73, 136.51, 136.43, 136.28, 136.24, 136.22, 134.45, 134.38, 134.30, 134.25, 134.18, 133.25, 131.75, 131.65, 131.60, 131.55, 130.57, 130.40, 130.11, 130.08, 129.05, 128.67, 128.24, 128.20, 128.14, 127.39, 127.21, 127.09, 127.06, 126.98, 126.94, 126.86, 126.71, 126.63, 125.82, 125.77, 125.63, 125.60, 125.37, 125.35, 124.90, 124.30, 124.24, 124.15, 124.09, 123.78, 123.70, 123.64, 123.58, 123.55, 123.52, 123.37, 118.28, 117.40, 117.19, 117.11, 116.97, 116.93, 116.70, 116.14, 115.50, 115.09, 114.88, 114.41, 114.28, 114.09, 112.91, 112.85, 112.80, 112.72, 112.60, 112.06, 111.54, 111.25, 105.36, 105.32, 105.28, 105.23, 93.80, 92.88, 92.80, 89.50, 89.44, 87.10, 82.84, 82.79, 82.78, 72.70, 71.13, 71.10, 70.94, 70.90, 70.77, 70.69, 70.39, 70.04, 70.01, 69.90, 69.79, 69.53, 69.46, 68.88, 68.81, 68.73, 68.62, 68.47, 68.44, 68.32, 68.25, 68.18, 68.08, 67.96, 67.79, 67.69, 67.58, 67.29, 65.80, 63.11, 62.98, 62.85, 62.41, 62.35, 62.30, 62.15, 61.86, 60.54, 50.42, 50.34, 50.31, 50.27, 50.23, 50.16, 50.09, 36.92, 36.82, 36.74, 34.74, 34.68, 34.55, 32.67, 31.44, 30.02.

HRMS (ESI-TOF): m/z: 1198.0335 (calcd. $[M+2Na]^{2+}$ = 1198.0354).

(*M*)-2.

^{1}H NMR (400 MHz, $CDCl_3$): δ = 8.53–8.50 (m, 1H), 8.42 (d, J = 8.0 Hz, 1H), 8.35–8.34 (m, 1H), 8.23–8.21 (m, 1H), 8.07–7.98 (m, 4H), 7.82–7.17 (m, 25H), 7.02–6.99 (m, 2H), 6.97–6.86 (m, 1H), 6.85–6.73 (m, 3H), 6.60–6.31 (m, 5H), 6.19–6.14 (m, 1H), 6.04–6.01 (m, 1H), 5.42–5.29 (m, 1H), 5.19–4.88 (m, 2H), 4.59–3.26 (m, 62H), 3.11–3.07 (m, 1H), 2.10–2.05 (m, 2H), 1.95–1.89 (m, 1H), 1.37–1.29 (m, 18H), 1.26–1.24 (m, 18H).

^{13}C NMR (100 MHz, $CDCl_3$): δ = 166.92, 166.85, 158.87, 153.96, 153.92, 153.81, 153.02, 151.85, 151.82, 151.54, 151.50, 151.47, 150.53, 150.49, 150.28, 150.24, 145.61, 145.54, 145.47, 145.36, 145.09, 145.03, 144.94, 144.81, 140.93, 140.86, 138.88, 138.84, 136.72, 136.50, 136.43, 136.27, 136.24, 136.21, 134.44, 134.35, 134.28, 134.21, 134.14, 133.25, 131.79, 131.68, 131.59, 131.55, 130.57, 130.40, 130.10, 130.08, 129.05, 128.66, 128.23, 128.19, 128.14, 127.37, 127.21, 127.08, 127.05, 127.00, 126.93, 126.85, 126.71, 126.61, 125.81, 125.76, 125.62, 125.59, 125.36, 125.34, 124.90, 124.31, 124.25, 124.17, 124.11, 123.80, 123.71, 123.67, 123.58, 123.55, 123.38, 118.25, 117.40, 117.16, 117.10, 116.96, 116.93, 116.63, 116.17, 115.53, 115.50, 115.10, 114.87, 114.44, 114.30, 114.10, 112.91, 112.85, 112.80, 112.71, 112.62, 112.59, 112.05, 111.54, 111.24, 111.18, 105.36, 105.31, 105.27, 105.22, 93.79, 92.89, 92.81, 89.48, 89.42, 87.10, 82.84, 82.79, 82.77, 72.78, 71.12, 71.08, 70.92, 70.86, 70.75, 70.65, 70.34, 70.04, 70.01, 69.90, 69.76, 69.53, 69.46, 68.84, 68.75, 68.62, 68.48, 68.43, 68.33, 68.28, 68.18, 68.08, 67.94, 67.70, 67.56, 67.20, 65.79, 63.09, 62.96, 62.83, 62.39, 62.32, 62.28, 62.13, 61.84, 60.50, 50.42, 50.32, 50.27, 50.22, 50.16, 50.07, 36.94, 36.92, 36.85, 36.77, 34.74, 34.67, 34.54, 32.67, 31.43, 30.01.

HRMS (ESI-TOF): m/z: 1198.0328 (calcd. $[M+2Na]^{2+}$ = 1198.0354).

***rac*-1.** To a mixture of ***rac*-2** (31 mg, 1.3 × 10^{-2} mmol), distillated triethylamine (367 μL, 2.64 mmol), and dichloromethane (10 mL), methacryloyl chloride (126 μL, 1.32 mmol) were added at 0 °C and the reaction mixture was stirred at r.t. for 20 h. The reaction was quenched by adding methanol (5 mL) at 0 °C, and the reaction mixture was poured into dichloromethane (200 mL) and washed with water (150 mL), and saturated aq. NaCl (150 mL). The organic layer was separated, dried over $MgSO_4$, filtered through celite, and the solvent was evaporated under reduced pressure. The crude product was purified by flash column chromatography on silica gel (eluent: gradient from dichloromethane to dichloromethane/acetone = 3:1 v/v) to afford ***rac*-1** (32 mg, 1.3× 10^{-2} mmol, 97%) as an orange solid.

^{1}H NMR (400 MHz, $CDCl_3$): δ = 8.53–8.49 (m, 1H), 8.43 (d, J = 8.0 Hz, 1H), 8.36–8.35 (m, 1H), 8.22–8.20 (m, 1H), 8.07–7.97 (m, 4H), 7.82–7.17 (m, 25H), 7.01–6.99 (m, 2H), 6.98–6.88 (m, 1H), 6.85–6.74 (m, 3H), 6.61–6.31 (m, 5H), 6.19–6.14 (m, 2H), 6.11–6.11 (m, 1H), 6.05–6.02 (m, 1H), 5.60–5.58 (m, 1H), 5.56–5.55 (m, 1H), 5.42–5.29 (m, 1H), 5.19–4.90 (m, 2H), 4.58–3.30 (m, 62H), 2.23–2.16 (m, 2H), 1.97–1.96 (m, 3H), 1.94–1.94 (m, 3H), 1.36–1.30 (m, 18H), 1.26–1.24 (m, 18H).

^{13}C NMR (100 MHz, $CDCl_3$): δ = 167.53, 167.50, 166.83, 166.80, 159.00, 153.97, 153.85, 153.07, 153.05, 151.88, 151.86, 151.40, 151.34, 150.66, 150.60, 150.32, 150.29, 150.26, 145.65, 145.60, 145.51, 145.42, 145.12, 145.07, 144.96, 144.85, 140.96, 140.87, 138.92, 138.88, 136.76, 136.48, 136.45, 136.41, 136.27, 134.49, 134.43, 134.37, 134.32, 134.22, 133.25, 131.75, 131.61, 130.59, 130.42, 130.13, 130.09, 129.07, 128.70, 128.27, 128.22, 128.15, 127.40, 127.24, 127.10, 127.08, 127.00, 126.97, 126.88, 126.72, 126.65, 125.96, 125.67, 125.38, 125.36, 124.91, 124.30, 124.22, 124.16, 124.11, 123.70, 123.64, 123.60, 123.55, 118.32, 118.29, 117.42, 117.24, 117.20, 117.13, 117.00, 116.96, 116.79, 116.76, 116.11, 115.49, 115.45, 114.89, 114.40, 114.27, 114.11, 112.99, 112.93, 112.89, 112.75, 112.67, 112.64, 111.86, 111.55, 111.29, 105.37, 105.34, 105.29, 105.24, 93.81, 92.89, 92.81, 89.53, 89.46, 87.10, 82.85, 82.81, 82.78, 71.13, 71.03, 70.94, 70.85, 70.81, 70.07, 70.03, 69.91, 69.84, 69.54, 69.47, 69.29, 68.73, 68.64, 68.62, 68.54, 68.47, 68.32, 68.20, 68.08, 67.99, 67.86, 67.80, 67.63, 64.94, 64.03, 63.19, 63.05, 62.91, 62.43, 62.38, 62.32, 62.18, 61.98, 50.43, 50.38, 50.26, 50.18, 50.11, 36.89, 36.82, 36.80, 36.74, 34.79, 34.74, 34.69, 34.56, 31.46, 30.01, 29.20, 18.49.

HRMS (ESI-TOF): m/z: 1266.0640 (calcd. $[M+2Na]^{2+}$ = 1266.0616).

The rotaxanes **(*P*)-1** and **(*M*)-1** were synthesized according to the same procedure used for ***rac*-1**. The rotaxane **(*P*)-1** was obtained as an orange solid (20 mg, 8.0 × 10^{-3} mmol, 80%) from **(*P*)-2** (24 mg, 1.0 × 10^{-2} mmol). The rotaxane **(*M*)-1** was obtained as an orange solid (25 mg, 1.0 × 10^{-2} mmol, 69%) from **(*M*)-2** (34 mg, 1.5 × 10^{-2} mmol).

(*P*)-1.

^{1}H NMR (400 MHz, $CDCl_3$): δ = 8.53–8.49 (m, 1H), 8.43 (d, J = 7.6 Hz, 1H), 8.36–8.35 (m, 1H), 8.22–8.20 (m, 1H), 8.07–7.98 (m, 4H), 7.82–7.17 (m, 25H), 7.01–6.99 (m, 2H), 6.98–6.88 (m, 1H), 6.85–6.74 (m, 3H), 6.60–6.31 (m, 5H), 6.19–6.14 (m, 2H), 6.11–6.11 (m, 1H), 6.05–6.02 (m, 1H), 5.59–5.58 (m, 1H), 5.56–5.55 (m, 1H), 5.42–5.29 (m, 1H), 5.18–4.90 (m, 2H), 4.58–3.33 (m, 62H), 2.23–2.16 (m, 2H), 1.96–1.96 (m, 3H), 1.94 (s, 3H), 1.36–1.30 (m, 18H), 1.26–1.24 (m, 18H).

^{13}C NMR (100 MHz, $CDCl_3$): δ =167.52, 166.87, 166.83, 159.01, 153.97, 153.87, 153.06, 152.97, 151.89, 151.41, 151.34, 150.67, 150.61, 150.31, 150.27, 145.66, 145.60, 145.52, 145.42, 145.12, 145.08, 144.97, 144.86, 140.97, 140.89, 138.88, 136.77, 136.50, 136.42, 136.28, 134.48, 134.43, 134.35, 134.27, 134.22, 133.26, 131.69, 131.64, 130.60, 130.42, 130.14, 130.11, 129.08, 128.72, 128.25, 128.23, 128.17, 127.42, 127.40, 127.24, 127.09, 127.02, 126.98, 126.89, 126.78, 126.74, 126.63, 125.96, 125.68, 125.39, 125.37, 124.92, 124.31, 124.26, 124.17, 124.10, 123.70, 123.65, 123.61, 123.55, 118.32, 118.30, 117.44, 117.22, 117.14, 117.01, 116.97, 116.74, 116.13, 114.90, 114.43, 114.32, 114.12, 113.00, 112.91, 112.76, 112.74, 112.65, 111.88, 111.56, 111.29, 111.27, 105.39, 105.35, 105.25, 105.16, 93.82, 92.90, 92.82, 89.53, 89.46, 87.11, 82.87, 82.80, 71.16, 71.13, 71.04, 70.97, 70.93, 70.85, 70.81, 70.08, 70.03, 69.92, 69.84, 69.55, 69.50, 69.31, 68.75, 68.64, 68.55, 68.50, 68.47, 68.33, 68.22, 68.08, 67.98, 67.82, 67.79, 67.64, 64.96, 64.04, 63.20, 63.08, 62.93, 62.43, 62.39, 62.34, 62.19, 61.99, 50.44, 50.37, 50.26, 50.19, 50.10,

36.98, 36.94, 36.86, 36.82, 36.77, 34.80, 34.75, 34.71, 34.57, 31.46, 30.02, 29.21, 18.50.

HRMS (ESI-TOF): m/z: 1266.0598 (calcd. $[M+2Na]^{2+}$ = 1266.0616).

(*M*)-1.

^{1}H NMR (400 MHz, $CDCl_3$): δ = 8.53–8.49 (m, 1H), 8.43 (d, J = 8.0 Hz, 1H), 8.36–8.35 (m, 1H), 8.22–8.20 (m, 1H), 8.07–7.98 (m, 4H), 7.82–7.17 (m, 25H), 7.01–6.99 (m, 2H), 6.98–6.88 (m, 1H), 6.85–6.74 (m, 3H), 6.60–6.31 (m, 5H), 6.19–6.14 (m, 2H), 6.11 (s, 1H), 6.05–6.02 (m, 1H), 5.59–5.58 (m, 1H), 5.56–5.55 (m, 1H), 5.42–5.29 (m, 1H), 5.18–4.90 (m, 2H), 4.58–3.31 (m, 62H), 2.23–2.16 (m, 2H), 1.97–1.96 (m, 3H), 1.94 (s, 3H), 1.36–1.30 (m, 18H), 1.26–1.24 (m, 18H).

^{13}C NMR (100 MHz, $CDCl_3$): δ = 167.52, 166.88, 166.84, 159.00, 154.00, 153.96, 153.84, 153.07, 153.04, 151.87, 151.85, 151.40, 150.66, 150.60, 150.30, 150.26, 145.64, 145.58, 145.50, 145.40, 145.11, 145.06, 144.95, 140.95, 140.88, 138.91, 138.87, 136.75, 136.48, 136.41, 136.26, 134.47, 134.40, 134.37, 134.33, 134.27, 134.19, 133.25, 131.82, 131.77, 131.69, 131.64, 131.58, 130.59, 130.42, 130.13, 130.10, 129.07, 128.69, 128.27, 128.22, 128.15, 127.39, 127.23, 127.10, 127.07, 127.01, 126.97, 126.87, 126.75, 126.73, 126.63, 125.97, 125.68, 125.38, 124.91, 124.29, 124.25, 124.21, 124.17, 124.13, 123.90, 123.80, 123.67, 123.64, 123.60, 123.55, 123.42, 118.28, 118.27, 117.42, 117.20, 117.13, 117.06, 116.99, 116.96, 116.91, 116.75, 116.72, 116.69, 116.11, 114.89, 114.12, 112.99, 112.94, 112.89, 112.74, 112.72, 112.63, 111.87, 111.55, 111.24, 105.38, 105.33, 105.19, 93.81, 92.89, 92.81, 89.51, 89.48, 89.44, 87.10, 82.85, 82.79, 71.11, 71.02, 70.90, 70.83, 70.80, 70.04, 69.92, 69.83, 69.56, 69.49, 69.29, 68.75, 68.64, 68.48, 68.46, 68.33, 68.19, 67.96, 67.78, 67.63, 64.94, 64.03, 63.18, 62.91, 62.42, 62.38, 62.31, 62.17, 61.99, 50.42, 50.39, 50.37, 50.31, 50.30, 50.25, 50.17, 50.14, 50.09, 36.95, 36.91, 36.85, 36.77, 34.79, 34.74, 34.56, 31.45, 30.01, 29.20, 18.49.

HRMS (ESI-TOF): m/z: 1266.0591 (calcd. $[M+2Na]^{2+}$ = 1266.0616).

Scheme S3. Synthesis of ***rac*-Ref-Hel**.

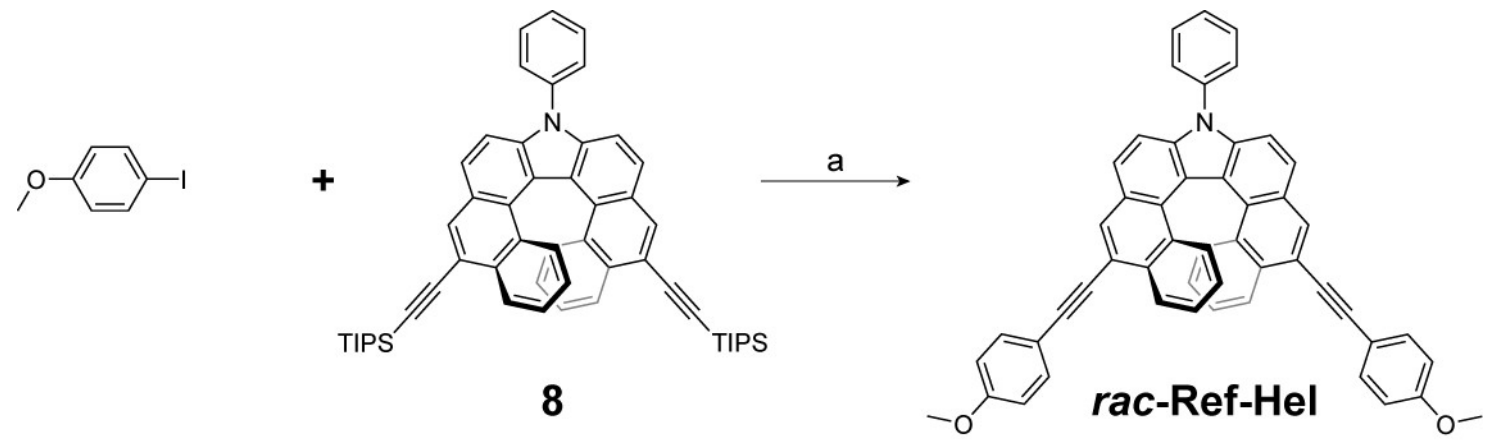

Conditions: (a) i) tetrabutylammonium fluoride, Et_3N, THF, r.t., 15 min, ii) $Pd(PPh_3)_4$, CuI, Et_3N, THF, 60 °C, overnight.

***rac*-Ref-Hel.** To a mixture of 4-iodoanisole (190 mg, 0.814 mmol), compound **8** (327 mg, 0.407 mmol), and triethylamine (10 mL) in THF (10 mL), a THF solution of tetrabutylammonium fluoride (ca. 1 mol/L, 1.02 mL, 1.02 mmol) was added and stirred at r.t. for 15 min. Then, $Pd(PPh_3)_4$ (23 mg, 2.0×10^{-2} mmol) and CuI (3.9 mg, 2.0×10^{-2} mmol) were added and the reaction mixture was stirred at 60 °C overnight. After cooling to r.t., the reaction mixture was poured into chloroform (150 mL) and washed with 5% aq. HCl (150 mL), saturated aq. $NaHCO_3$ (150 mL), and saturated aq. NaCl (150 mL). The organic layer was separated, dried over $MgSO_4$, filtered, and the solvent was evaporated under reduced pressure. The crude product was purified by flash column chromatography on silica gel (eluent: gradient from hexane/dichloromethane = 9:1 v/v to hexane/dichloromethane = 11:9 v/v) to afford ***rac*-Ref-Hel** (230 mg, 3.27×10^{-1} mmol, 80%) as a yellow solid.
^{1}H NMR (400 MHz, $CDCl_3$): δ = 8.50 (d, J = 8.0 Hz, 2H), 8.33 (s, 2H), 8.00 (d, J = 8.4 Hz, 2H), 7.76 (d, J = 8.8 Hz, 2H), 7.73–7.62 (m, 9H), 7.50 (d, J = 8.0 Hz, 2H), 7.35–7.31 (m, 2H), 6.97 (d, J = 8.4 Hz, 4H), 6.37 (t, J = 7.6 Hz, 2H), 3.88 (s, 6H).
^{13}C NMR (100 MHz, $CDCl_3$): δ = 159.75, 140.84, 136.94, 133.28, 131.52, 130.59, 130.34, 130.22, 128.95, 128.68, 128.33, 127.16, 127.12, 126.98, 126.76, 124.86, 123.50, 117.35, 117.19, 115.98, 114.25, 111.35, 93.70, 87.09, 55.52.
HRMS (ESI-TOF): m/z: 726.2386 (calcd. $[M+Na]^+$ = 726.2404).

Enantioseparation of *rac*-Ref-Hel
Compound ***rac*-Ref-Hel** (120 mg) was subjected to recycle chiral HPLC (eluent: hexane/dichloromethane = 1:1 v/v). The separated fractions were collected and concentrated to yield **(*P*)-Ref-Hel** (56 mg, first fraction) and **(*M*)-Ref-Hel** (58 mg, second fraction).
(*P*)-Ref-Hel.
^{1}H NMR (400 MHz, $CDCl_3$): δ = 8.48 (d, J = 8.4 Hz, 2H), 8.34 (s, 2H), 8.02 (d, J = 8.8 Hz, 2H), 7.79 (d, J = 8.4 Hz, 2H), 7.76–7.73 (m, 4H), 7.70–7.65 (m, 5H), 7.50 (d, J = 8.4 Hz, 2H), 7.32 (t, J = 7.2 Hz, 2H), 6.98 (d, J = 8.8 Hz, 4H), 6.38–6.34 (m, 2H), 3.89 (s, 6H).
^{13}C NMR (100 MHz, $CDCl_3$): δ = 159.75, 140.84, 136.95, 133.28, 131.52, 130.58, 130.35, 130.22, 128.95, 128.69, 128.33, 127.16, 127.12, 126.98, 126.76, 124.86, 123.51, 117.35, 117.20, 115.98, 114.25, 111.36, 93.69, 87.09, 55.52.
HRMS (ESI-TOF): m/z: 726.2391 (calcd. $[M+Na]^+$ = 726.2404).
(*M*)-Ref-Hel.
^{1}H NMR (400 MHz, $CDCl_3$): δ = 8.48 (d, J = 8.4 Hz, 2H), 8.34 (s, 2H), 8.02 (d, J = 8.8 Hz, 2H), 7.79 (d, J = 8.4 Hz, 2H), 7.76–7.73 (m, 4H), 7.70–7.65 (m, 5H), 7.49 (d, J = 8.4 Hz, 2H), 7.34–7.30 (m, 2H), 6.98 (d, J = 8.8 Hz, 4H), 6.38–6.34 (m, 2H), 3.89 (s, 6H).
^{13}C NMR (100 MHz, $CDCl_3$): δ = 159.76, 140.85, 136.95, 133.28, 131.52, 130.59, 130.35, 130.22, 128.95, 128.69, 128.33, 127.16, 127.13, 126.99, 126.76, 124.86, 123.51, 117.35, 117.20, 115.98, 114.25, 111.36, 93.70, 87.09, 55.52.
HRMS (ESI-TOF): m/z: 726.2406 (calcd. $[M+Na]^+$ = 726.2404).

Scheme S4. Synthesis of compound **13**

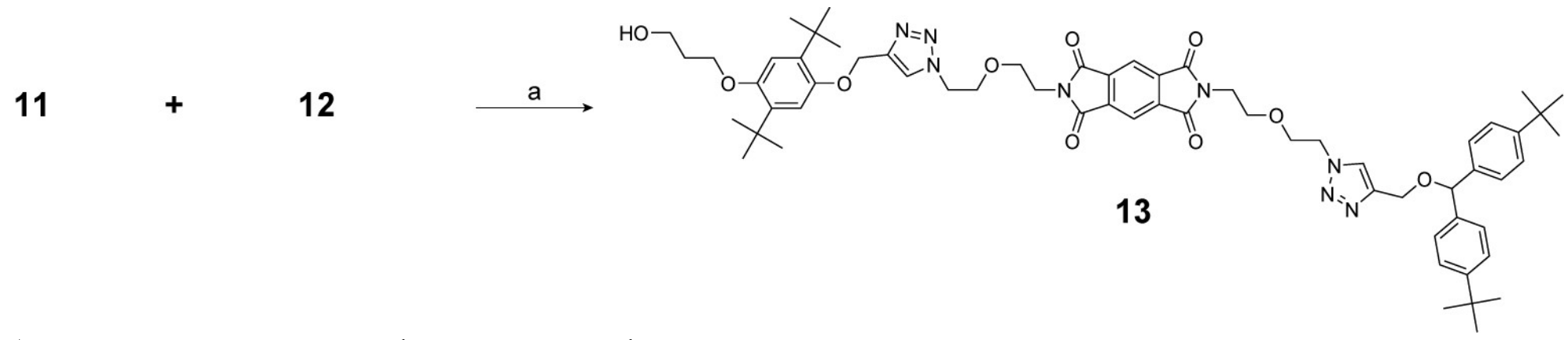

Conditions: (a) CuI, *i*-Pr_2EtN, $CHCl_3$, 40 °C, 15 h

Compound 13. A mixture of compound **11** (16 mg, 5.1×10^{-2} mmol), compound **12** (36 mg, 4.6×10^{-2} mmol), CuI (8.8 mg, 4.6×10^{-2} mmol), and *i*-Pr_2EtN (300 μL) in chloroform (20 mL) was stirred for 15 h at 40 °C. The reaction mixture was poured into chloroform (100 mL), and the solution was washed with 5% aq. HCl (100 mL), saturated aq. $NaHCO_3$ (100 mL), and saturated aq. NaCl (100 mL). The organic layer was dried over $MgSO_4$ and filtered, and the solvent was evaporated under reduced pressure. The crude product thus isolated was purified by flash column chromatography on silica gel (eluent: gradient from dichloromethane/acetone = 9:1 v/v to dichloromethane/acetone = 4:1 v/v) to afford compound **13** (47 mg, 4.3×10^{-2} mmol, 93%) as a pale yellow solid.

^{1}H NMR (400 MHz, $CDCl_3$): δ = 8.24 (s, 2H), 7.74 (s, 1H), 7.69 (s, 1H), 7.34 (d, J = 8.8 Hz, 4H), 7.30 (d, J = 8.4 Hz, 4H), 6.97 (s, 1H), 6.85 (s, 1H), 5.48 (s, 1H), 5.18 (s, 2H), 4.58 (s, 2H), 4.49–4.44 (m, 4H), 4.10 (t, J = 6.0 Hz, 2H), 3.93–3.88 (m, 6H), 3.85–3.81 (m, 4H), 3.70–3.65 (m, 4H), 2.63 (s, 1H), 2.11–2.05 (m, 2H), 1.37 (s, 9H), 1.36 (s, 9H), 1.28 (s, 18H).

^{13}C NMR (100 MHz, $CDCl_3$): δ = 166.04, 166.03, 151.51, 150.42, 150.29, 145.43, 144.91, 138.83, 137.17, 136.46, 136.15, 126.85, 125.35, 123.95, 123.73, 118.44, 112.92, 111.90, 82.59, 69.01, 68.95, 67.96, 65.60, 63.07, 62.24, 60.23, 50.29, 50.15, 37.91, 37.83, 34.69, 34.66, 34.51, 32.65, 31.39, 29.94, 29.30.

MS (MALDI-TOF): m/z: 1118.01 (calcd. $[M+Na]^+$ = 1117.57).

Theoretical Calculations

Theoretical calculations of electron-rich 1,5-dimethoxynaphthalene and π-extended **A7Hel** and electron-deficient PMDI were conducted.

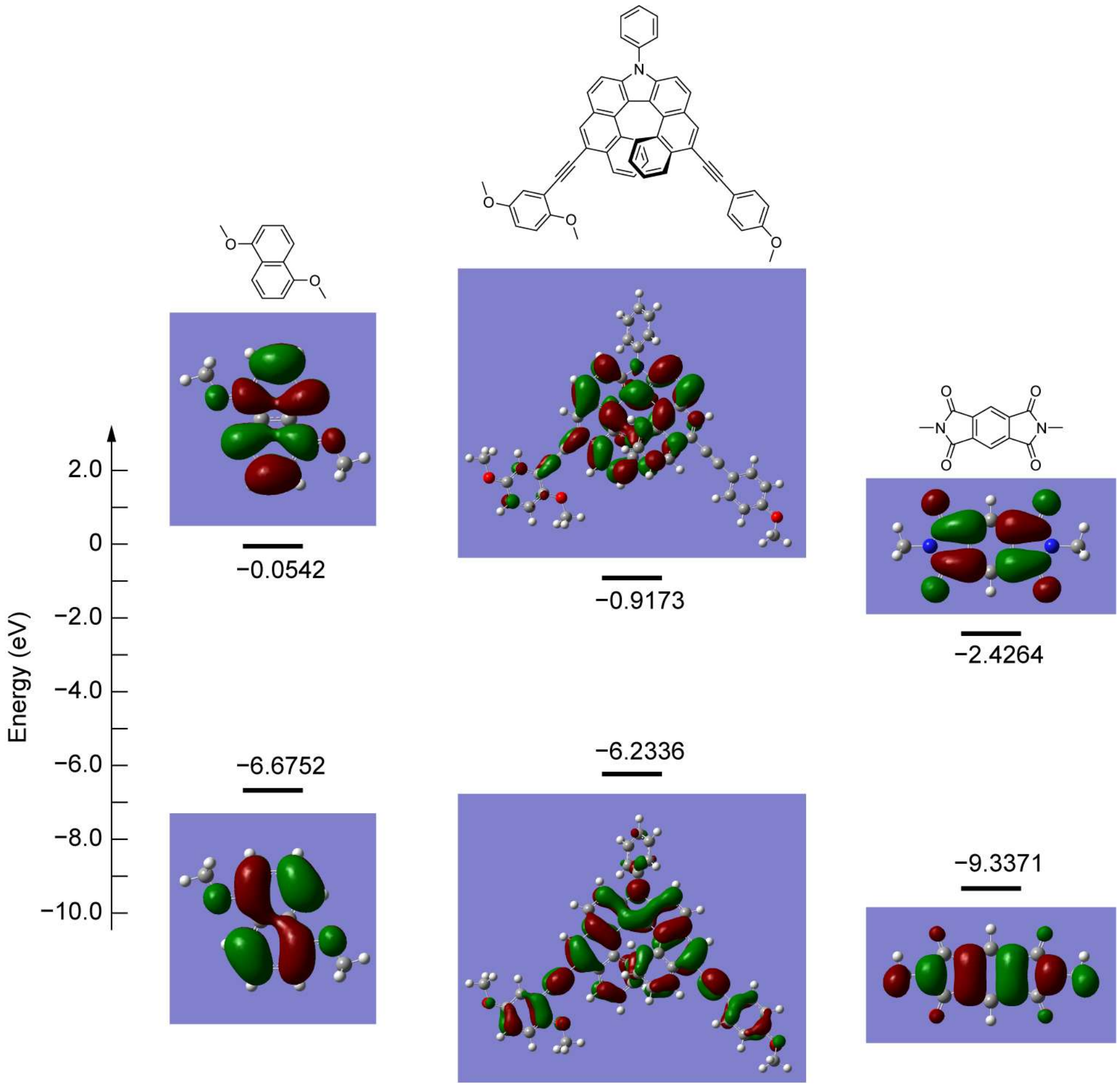

Figure S1. Frontier molecular orbitals (HOMO and LUMO) and their energy levels (in eV) for the electron-rich and electron-deficient moieties of the rotaxane, calculated at the M06-2X/def2-TZVP level.

Determination of the Absolute Configuration of Helicenes

The absolute configuration of the helicene macrocycle **3** was determined by comparing the circular dichroism (CD) signs with those of a reference compound, **Ref-Hel**. Compounds **3** and **4** showed low crystallinity and were therefore not suitable for X-ray single-crystal analysis. Enantioseparation of ***rac*-Ref-Hel** by recycling preparative chiral HPLC was confirmed by analytical chiral HPLC (Figure S2). The first eluted fraction was collected and recrystallized to yield crystals suitable for X-ray single-crystal analysis. The analysis confirmed that the enantiomer in the first fraction possesses (*P*)-helicity (Figure S3, CCDC 2543889). The CD spectra of the enantiomerically pure **Ref-Hel** (obtained from the first and second fractions) and the enantiomerically pure **3** (synthesized from the first and second fractions of ***rac*-4**) were measured in chloroform (Figure S4). The CD spectrum of **(*P*)-Ref-Hel** (first fraction) showed a small negative Cotton effect at 430 nm, followed by large positive Cotton effects at 370 and 355 nm. Compound **3** synthesized from the first fraction of ***rac*-4** exhibited a nearly identical CD spectrum to that of **(*P*)-Ref-Hel**. Based on this spectral comparison, the first fraction of ***rac*-4**, which was separated as shown in Figure S5, was assigned as **(*P*)-4**, and the second fraction as **(*M*)-4**.

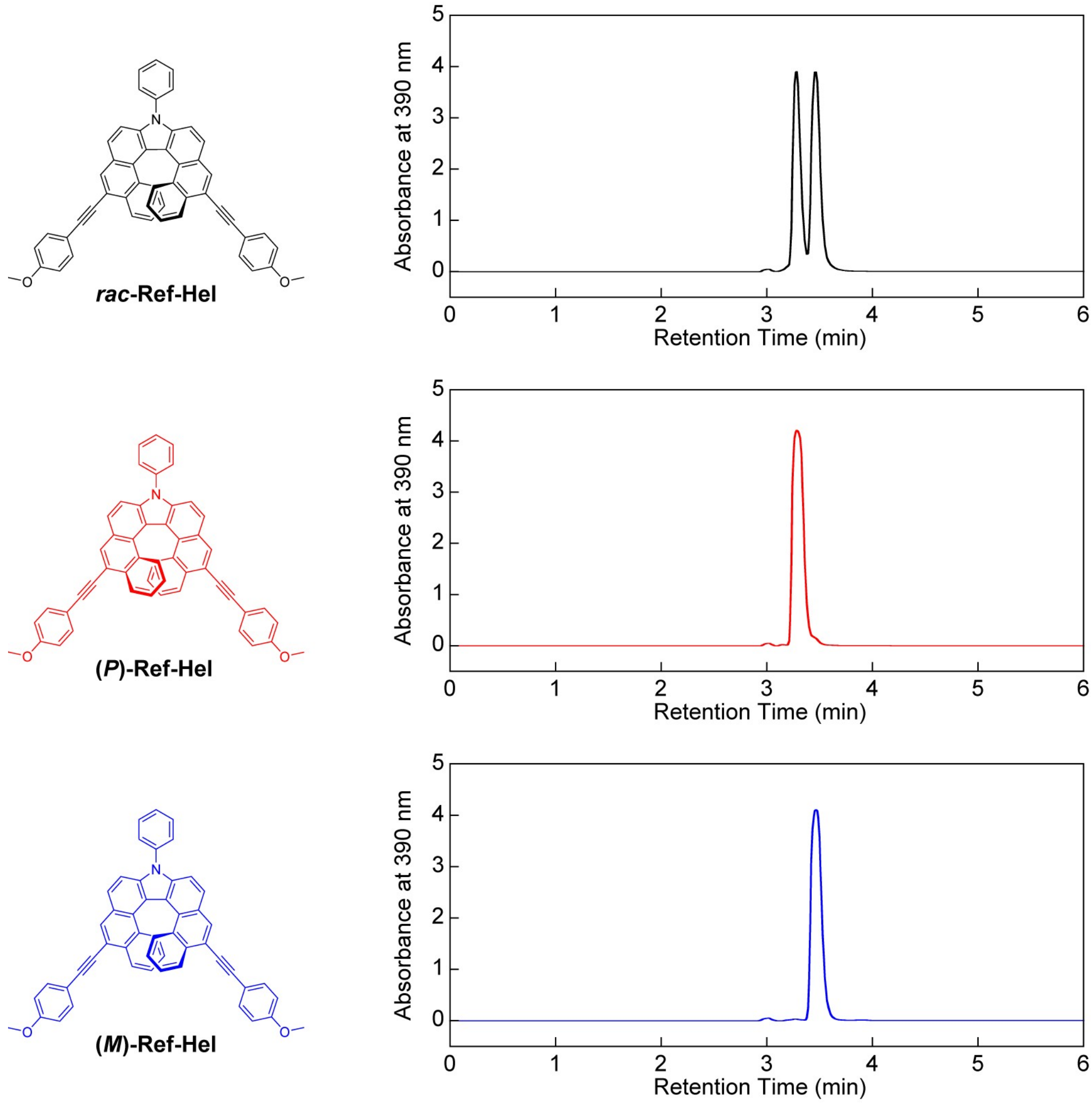

Figure S2. Enantioseparation of **Ref-Hel**. Analytical chiral HPLC chromatogram of ***rac*-Ref-Hel** (top), its isolated first fraction (**(*P*)-Ref-Hel**, middle), and second fraction (**(*M*)-Ref-Hel**, bottom). Column: DAICEL CHIRALPAK IG. Eluent: gradient from hexane/dichloromethane = 5:5 v/v to hexane/dichloromethane = 3:7 v/v. Flow rate: 1.0 mL min^{-1}. Detection wavelength: 390 nm.

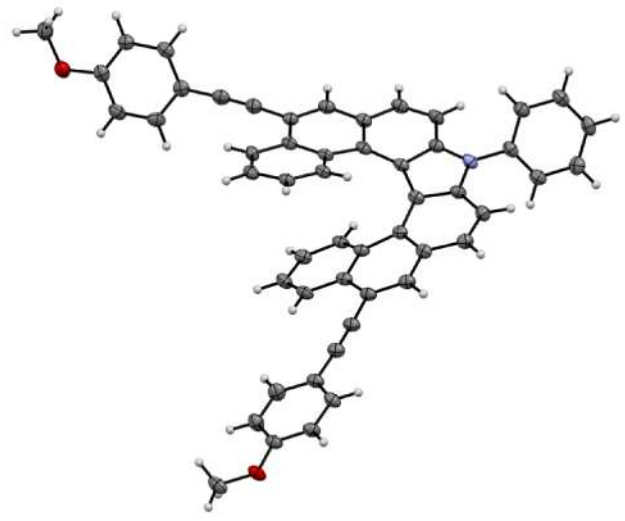

Figure S3. ORTEP drawing of ***(P)*-Ref-Hel**, obtained from the first fraction, determined by X-ray crystallography. Thermal ellipsoids are set at 50% probability.

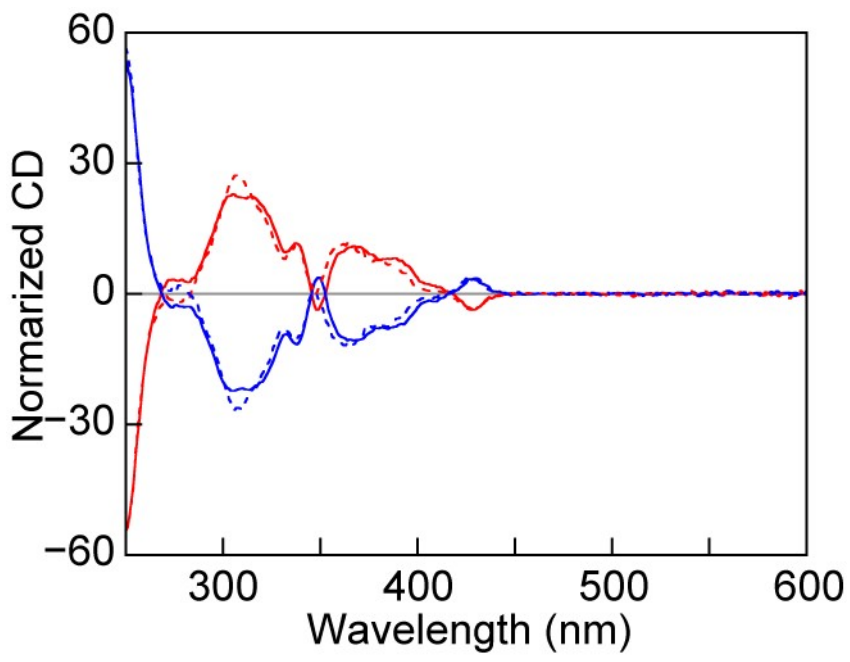

Figure S4. Normalized CD spectra of enantiomerically pure **3** (red solid line: synthesized from the first fraction of ***rac*-4**, blue solid line: synthesized from the second fraction of ***rac*-4**) and **Ref-Hel** (red dotted line: first fraction, blue dotted line: second fraction).

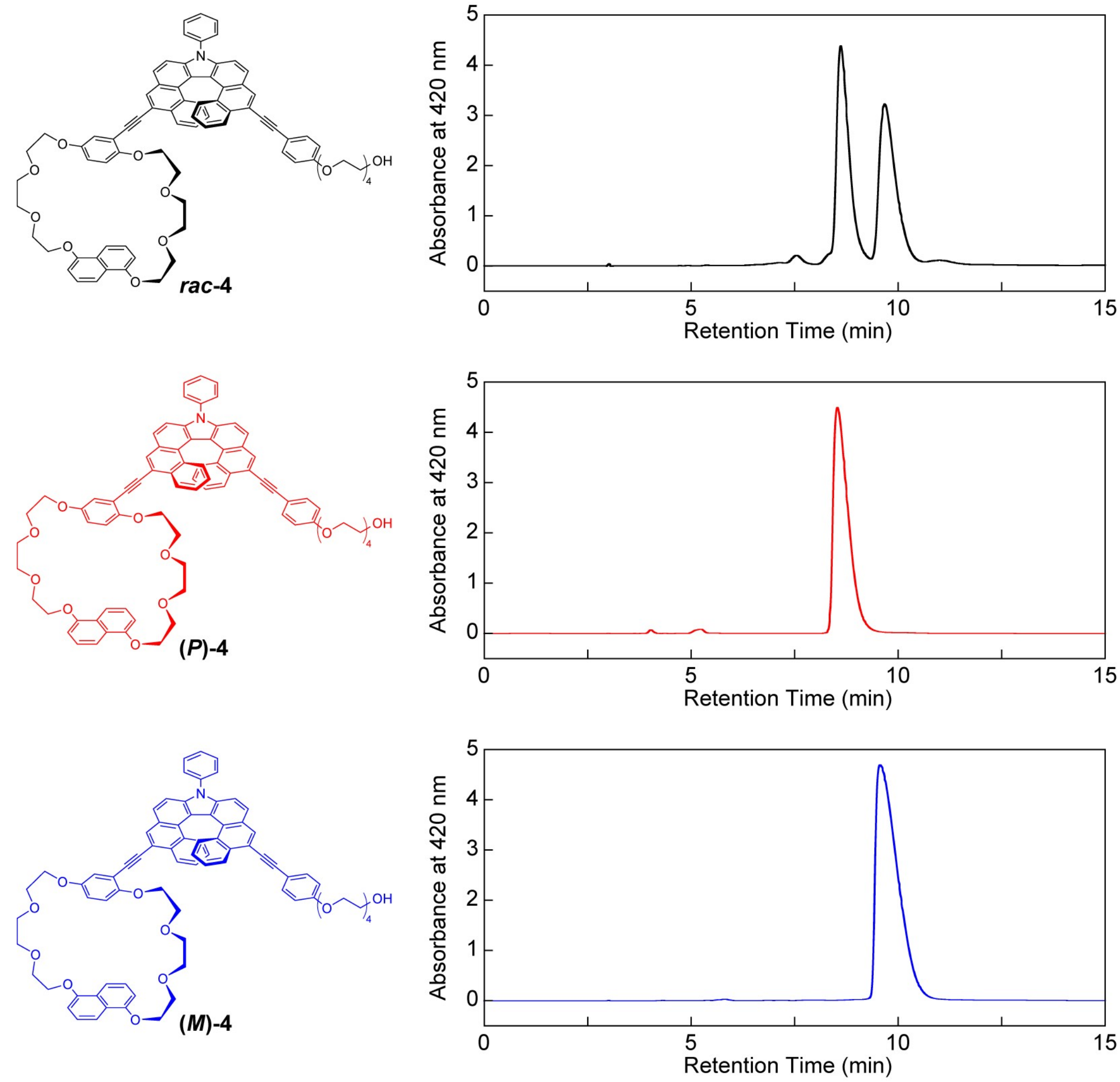

Figure S5. Enantioseparation of helicene macrocycle **4**. Analytical chiral HPLC chromatogram of ***rac*-4** (top), and its isolated first fraction (**(*P*)-4**, middle) and second fraction (**(*M*)-4**, bottom). Column: DAICEL CHIRALPAK IG. Eluent: hexane/dichloromethane/ethanol = 9:9:2 v/v/v. Flow rate: 1.0 mL min^{-1}. Detection wavelength: 420 nm.

NMR Analyses

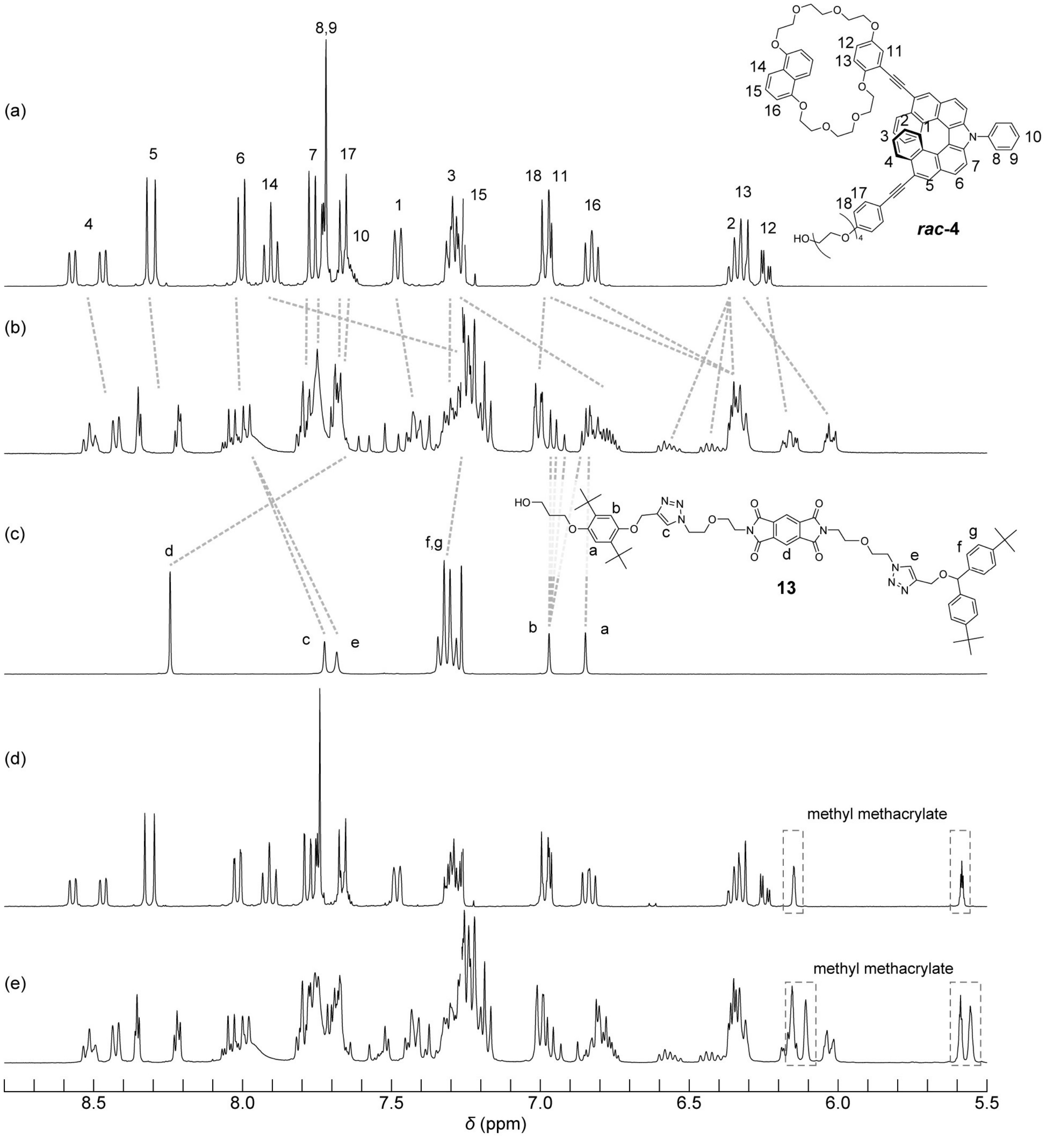

Figure S6. Partial ^{1}H NMR spectra of (a) ring ***rac*-4**, (b) rotaxane ***rac*-2**, (c) axle **13**, (d) ring ***rac*-3**, and (e) rotaxane ***rac*-1**.

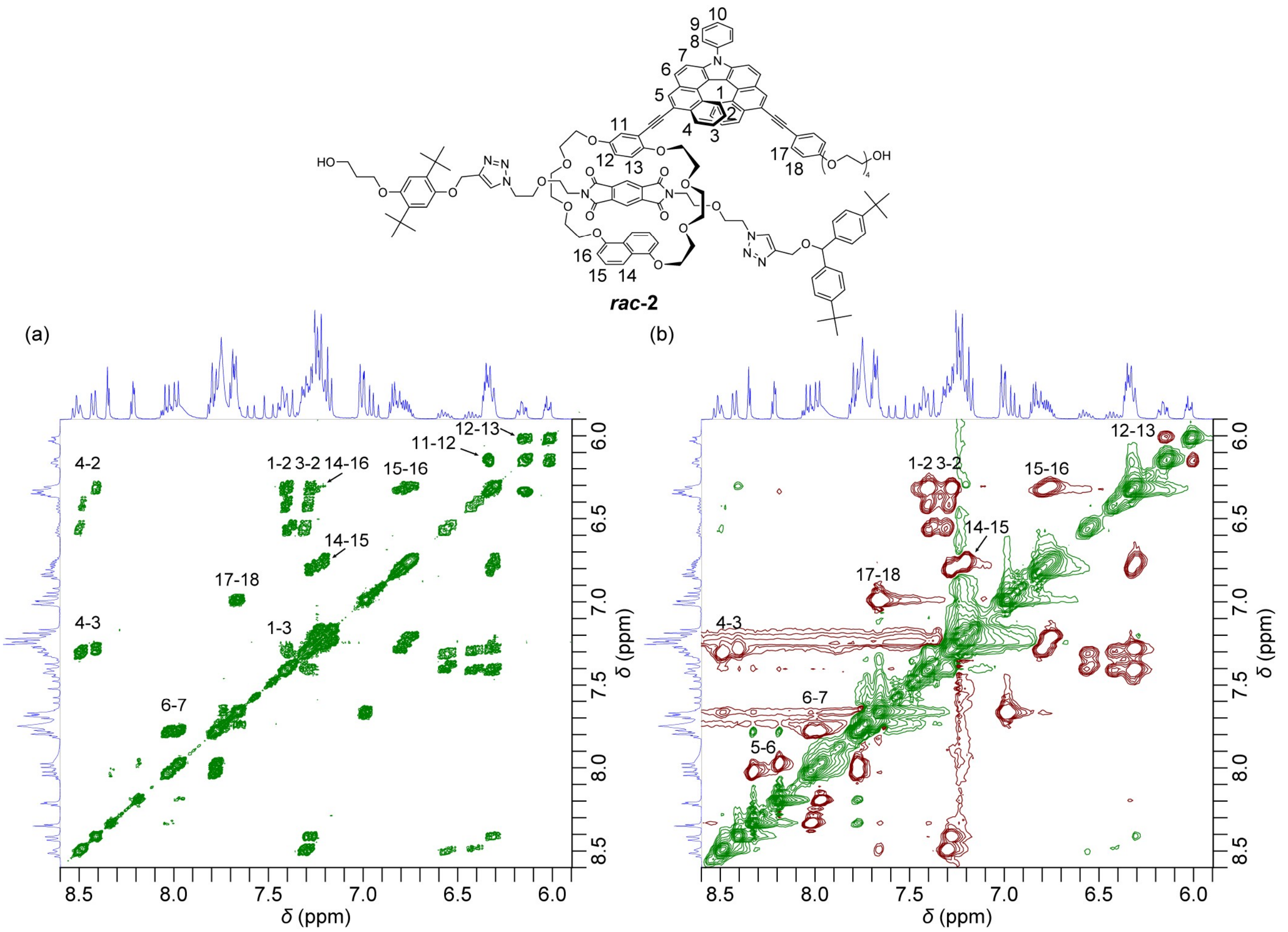

Figure S7. (a) Partial ^{1}H-^{1}H COSY NMR spectrum and (b) partial ^{1}H-^{1}H ROESY NMR spectrum of ***rac*-2**.

Isomeric Analyses of Co-conformational Regioisomers

Analytical reversed-phase HPLC chromatograms of ***rac*-1** and ***rac*-2** exhibited a single sharp peak under two different solvent conditions (Figure S8). Although the rotaxane products contain almost no impurities, four distinct co-conformational regioisomers (isomers **A–D**, Figure S9) are inherently possible for each rotaxane containing a macrocycle with a specific helical chirality of **A7Hel** (e.g., the (*P*)-isomer). The unsymmetrical nature of both the axle and the macrocycle allows different relative orientations of the macrocycle on the axle, giving rise to these isomers. Because there is no enantiomeric relationship among isomers **A–D**, they are diastereomeric to each other.

The existence of these diastereomeric regioisomers was clearly evidenced by the ^{1}H NMR spectra (Figure S10). Specifically, the benzylic methylene protons on the diphenylmethane moiety (H_a) and the triazole-adjacent methylene protons (H_b) on the axle are resolved into four distinct sets of signals (H_{a1}–H_{a4} and H_{b1}–H_{b4}, respectively). The integration ratios of the four H_a signals correspond to those of the four H_b signals, confirming the coexistence of the four regioisomers. Crucially, the relative ratios of these four isomers are almost the same among ***rac*-1**, **(*P*)-1**, and **(*M*)-1**. Thus, the presence of these multiple regioisomers does not perturb the bulk chiroptical properties, ensuring mirror-image signals.

Notably, H_{a3} and H_{a4} show distinctive geminal coupling, indicating a strongly diastereotopic environment. These signals would be ascribed to isomer **B** and/or **D**, where the chiral helicene fluorophore faces the H_a side.

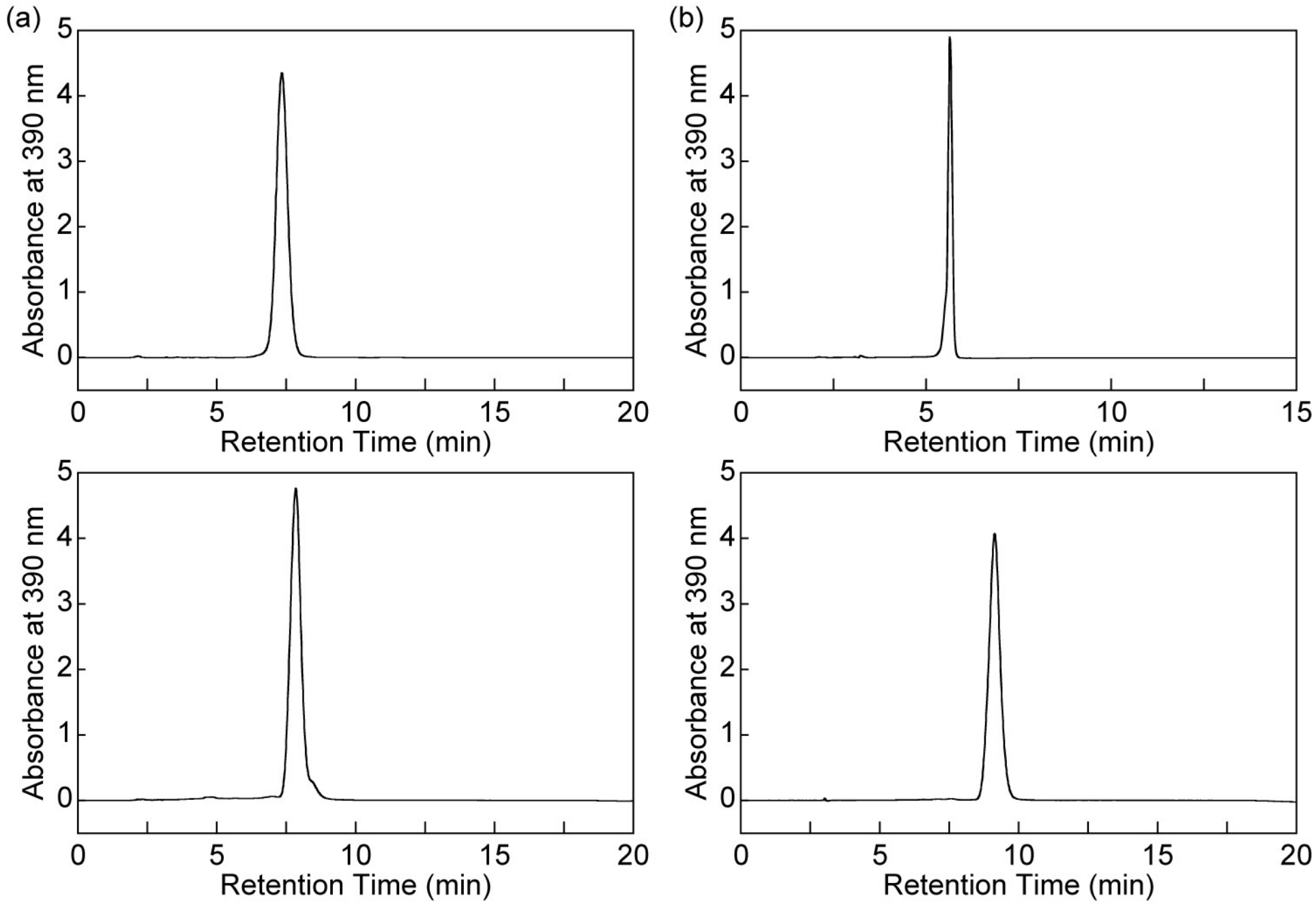

Figure S8. Analytical reversed-phase HPLC chromatograms of (a) ***rac*-2** (eluent (top): gradient from methanol/dichloromethane = 17:3 v/v to methanol/dichloromethane = 4:1 v/v, eluent (bottom): gradient from methanol/acetone = 4:1 v/v to methanol/acetone = 1:1 v/v) and (b) ***rac*-1** (eluent (top): gradient from methanol/dichloromethane = 13:7 v/v to methanol/dichloromethane = 3:2 v/v, eluent (bottom): gradient from methanol/acetone = 7:3 v/v to methanol/acetone = 2:3 v/v) monitored at 390 nm.

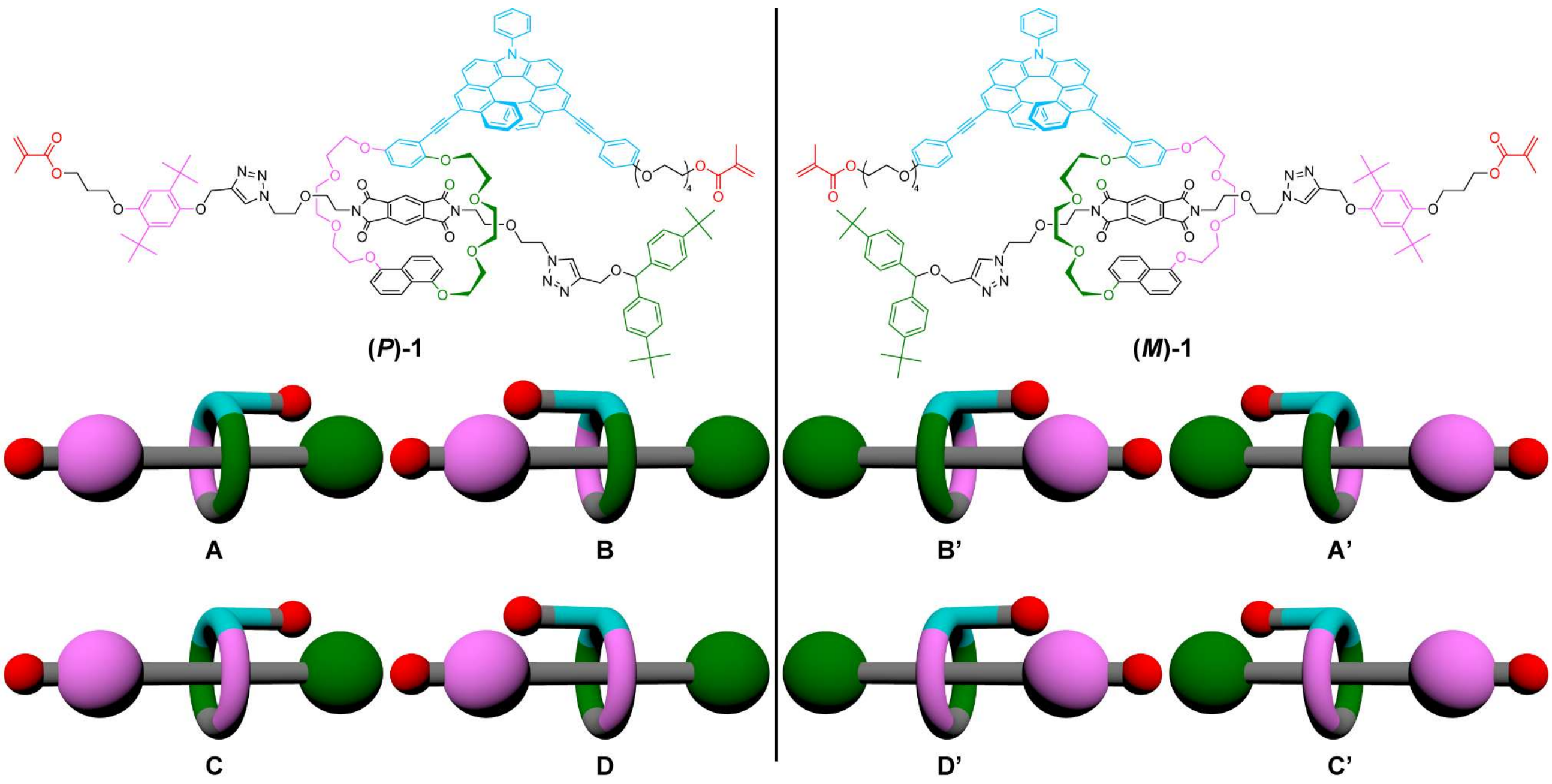

Figure S9. Schematic illustrations of the four co-conformational regioisomers for **(*P*)/(*M*)-1**.

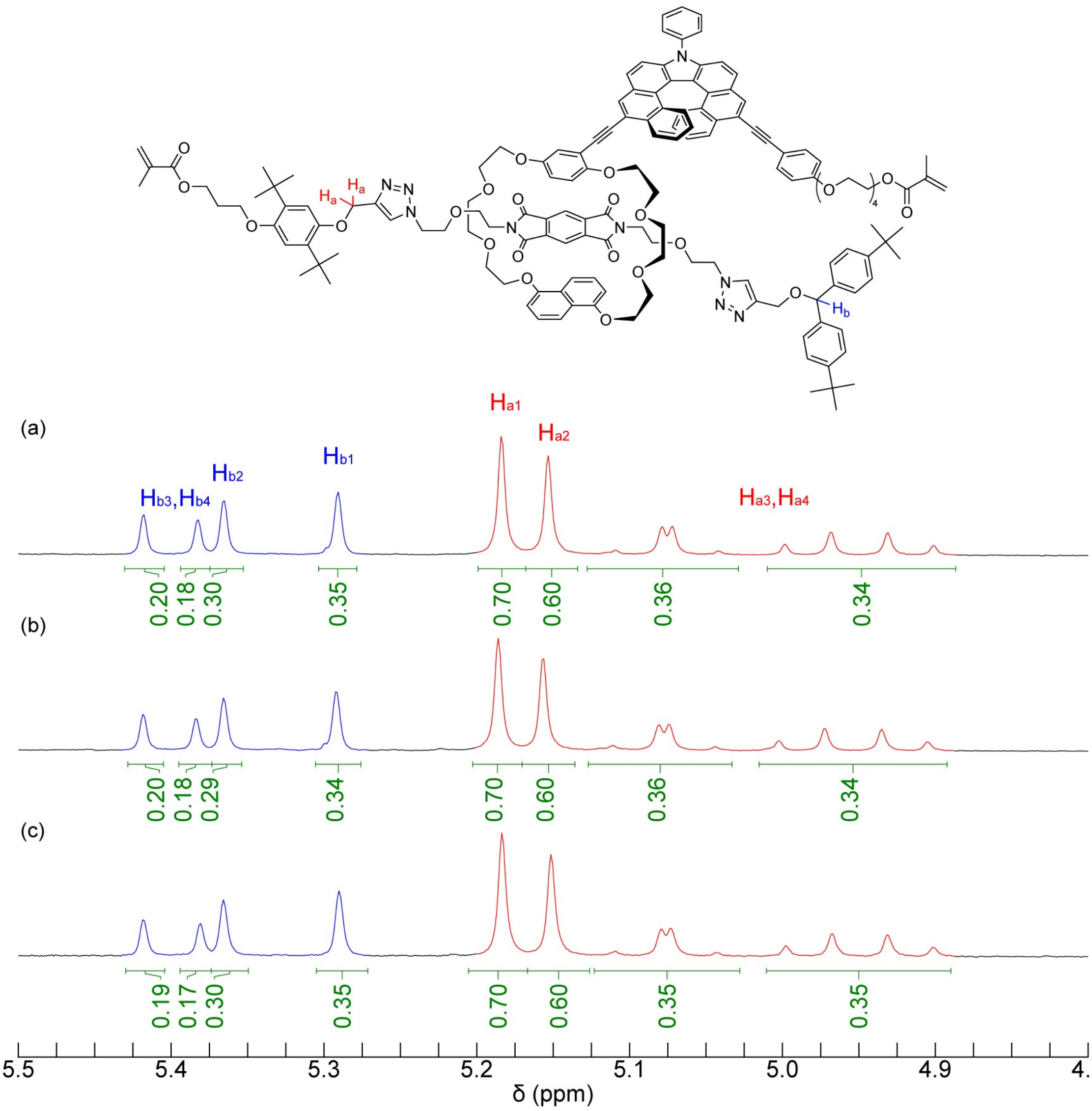

Figure S10. Partial ^{1}H NMR spectra of (a) ***rac*-1**, (b) **(*P*)-1**, and (c) **(*M*)-1**. The green numbers are the relative integration values of the respective split signals.

Optical Properties of Rings and Rotaxanes in Solutions

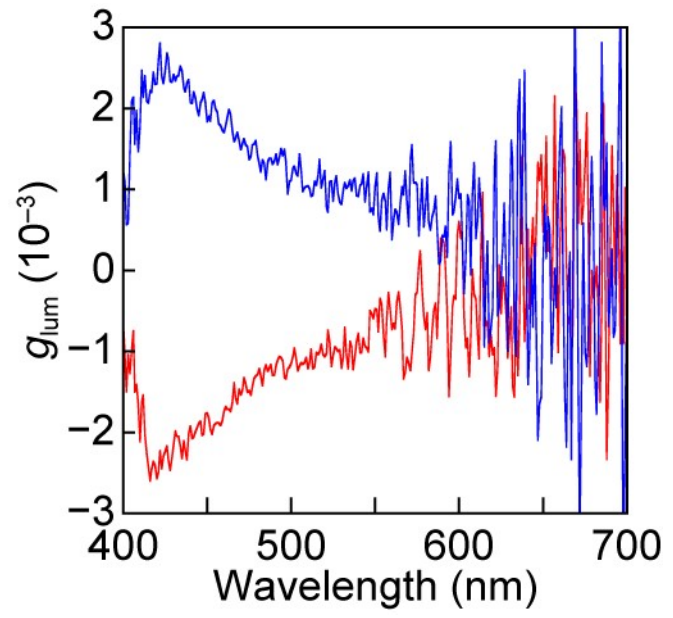

Figure S11. Mirror-image g_{lum} spectra of ***(P)*-3** (red) and ***(M)*-3** (blue) in chloroform (c = 1.0 × 10^{-5} M). λ_{ex} = 330 nm.

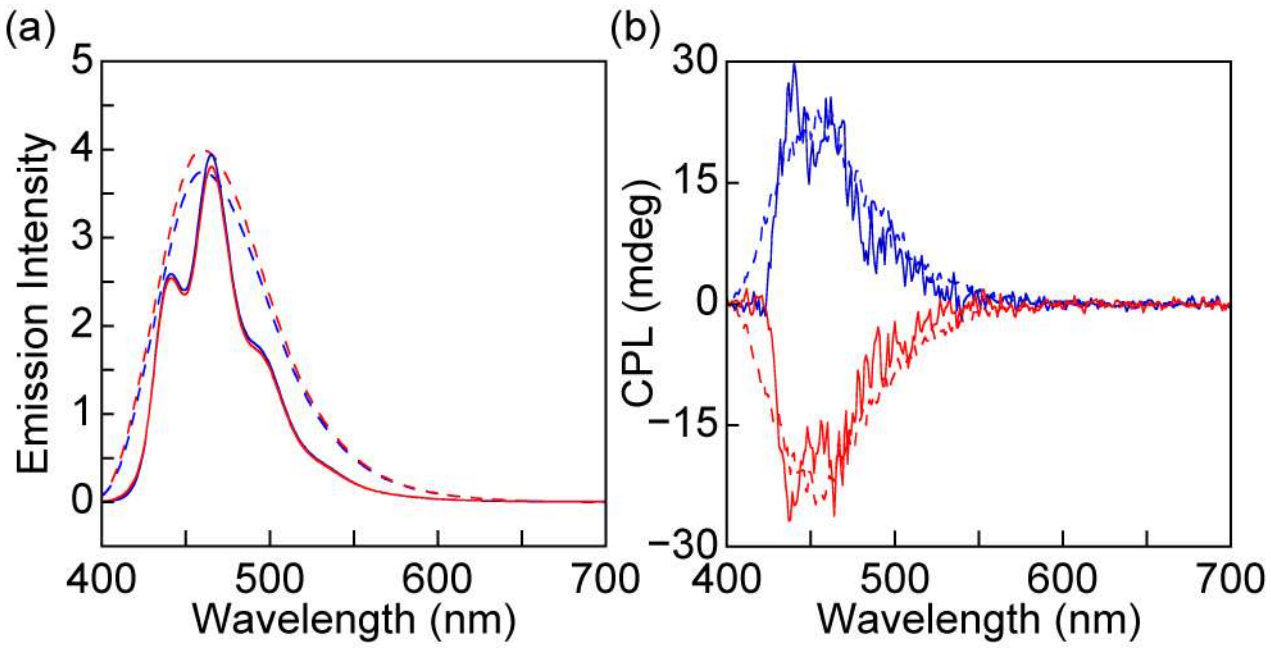

Figure S12. (a) Photoluminescence and (b) CPL spectra of ***(P)*-3** (red solid line: with the emission slit width of 0.5 mm, red dotted line: with the slit width of 4.0 mm) and ***(M)*-3** (blue solid line: with the slit width of 0.5 mm, blue dotted line: with the slit width of 4.0 mm) in chloroform (c = 1.0 × 10^{-5} M). λ_{ex} = 330 nm.

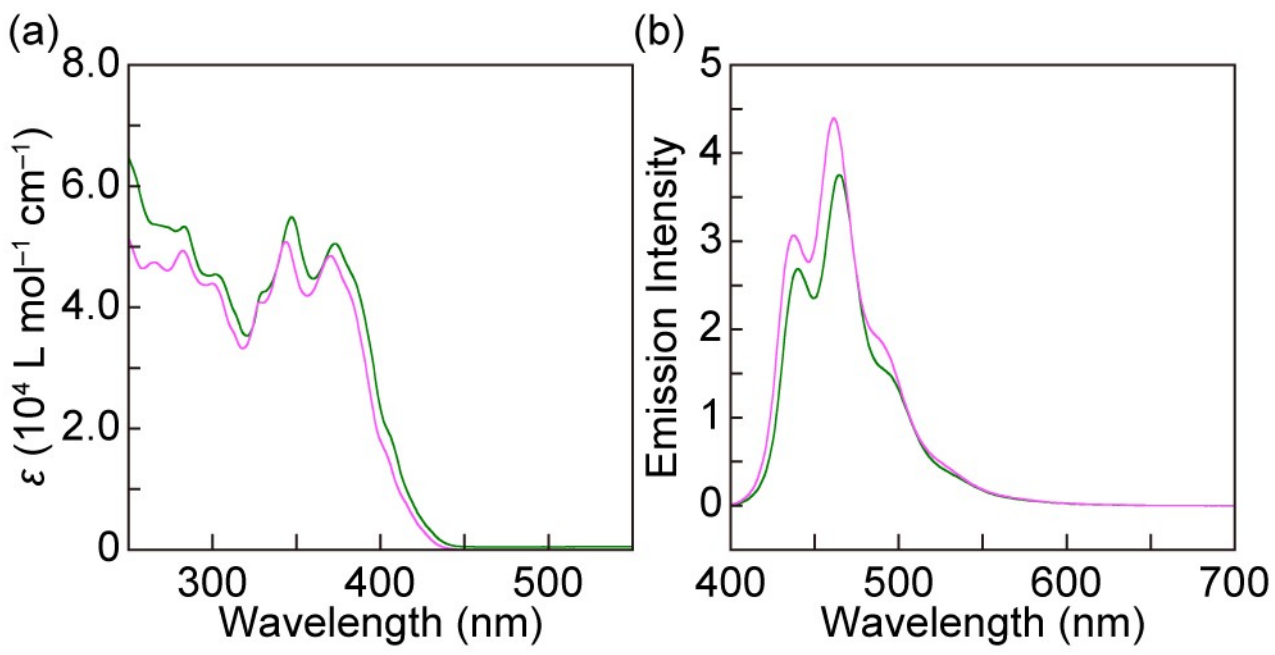

Figure S13. (a) Absorption and (b) photoluminescence spectra of ***rac*-4** in chloroform (green) and in methanol/chloroform = 199:1 v/v (pink) (c = 1.0 × 10^{-5} M). λ_{ex} = 330 nm.

Preparation of DN Dried Films and Gels

Preparation of 1-SN-DF

A solution of MeOEtMA (1.40 g, 9.71 mmol), K-SPMA (266 mg, 1.08 mmol), PEGdMA (300 μL, 0.430 mmol), and ***rac*-1** (10 mg, 4.0 μmol) in NMF (6 mL) was degassed with nitrogen gas bubbling for 15 min and moved into a glovebox. Following the addition of the thermal initiator V-70 (17 mg, 5.4×10^{-2} mmol), the homogeneous solution was poured into a mold (ca. 30.0 mm × ca. 20.0 mm × 0.50 mm) assembled from two glass substrates and a silicone spacer (thickness = 0.50 mm), which was then allowed to stand at r.t. for 14 h. The obtained SN gel ***rac*-1-SN-G** was washed three times by sequential solvent exchange with chloroform and methanol to remove unreacted species. The washed gels were dried under vacuum for over 3 h to afford dried films ***rac*-1-SN-DF**. The dried films **(*P*)-1-SN-DF** and **(*M*)-1-SN-DF** were prepared in the same manner using **(*P*)-1** and **(*M*)-1** as the cross-linker, respectively.

Preparation of 1-DN-G

The ***rac*-1-SN-DF** film was immersed in a solution of MeOEtMA (60.6 g, 0.420 mol), PEGdMA (584 μL, 0.841 mmol), and the thermal initiator AIBN (138 mg, 0.840 mmol) in NMF (40 mL) at r.t. for 14 h in a glovebox. After swelling, the gel was removed from the solution, sandwiched between two glass plates, and placed on a hot plate. The second polymerization was initiated by heating to 50 °C and maintaining that temperature for 20 h. The resultant DN gel ***rac*-1-DN-G** was washed three times by sequential solvent exchange with chloroform and methanol to remove unreacted species. Methanol-swollen ***rac*-1-DN-G** was dried under vacuum overnight (for at least 12 h) to afford the dried elastomer film ***rac*-1-DN-DF**. Dried films **(*P*)-1-DN-DF** and **(*M*)-1-DN-DF** were prepared in the same manner using **(*P*)-1-SN-DF** and **(*M*)-1-SN-DF**, respectively. DN gels ***rac*/(*P*)/(*M*)-1-DN-G** were obtained by swelling the corresponding dried films with chloroform or methanol.

Preparation of 3-SN-DF

A solution of MeOEtMA (0.700 g, 4.86 mmol), K-SPMA (133 mg, 0.540 mmol), PEGdMA (150 μL, 0.215 mmol), and ***rac*-3** (2.7 mg, 2.0 μmol) in NMF (2 mL) and dimethylsulfoxide (1 mL) was degassed with nitrogen gas bubbling for 15 min and moved into a glovebox. Following the addition of the thermal initiator V-70 (8.3 mg, 2.7×10^{-2} mmol), the homogeneous solution was poured into a mold (ca. 30.0 mm × ca. 20.0 mm × 0.50 mm) assembled from two glass substrates and a silicone spacer (thickness = 0.50 mm), which was then allowed to stand at r.t. for 14 h. The obtained SN gel was washed three times by sequential solvent exchange with chloroform and methanol to remove unreacted species. The washed gels were dried under vacuum for over 3 h to afford dried films ***rac*-3-SN-DF**. The dried films **(*P*)-3-SN-DF** and **(*M*)-3-SN-DF** were prepared in the same manner using **(*P*)-3** and **(*M*)-3** as the monomer, respectively.

Preparation of 3-DN-G

The ***rac*-3-SN-DF** film was immersed in a solution of MeOEtMA (60.6 g, 0.420 mol), PEGdMA (584 μL, 0.841 mmol), and the thermal initiator AIBN (138 mg, 0.840 mmol) in NMF (40 mL) at r.t. for 14 h in a glovebox. After swelling, the gel was removed from the solution, sandwiched between two glass plates, and placed on a hot plate. The second polymerization was initiated by heating to 50 °C and maintaining that temperature for 20 h. The resultant DN gel ***rac*-3-DN-G** was washed three times by sequential solvent exchange with chloroform and methanol to remove unreacted species. Methanol-swollen ***rac*-3-DN-G** was dried under vacuum overnight (for at least 12 h) to afford the dried elastomer film ***rac*-3-DN-DF**. Dried films **(*P*)-3-DN-DF** and **(*M*)-3-DN-DF** were prepared in the same manner using **(*P*)-3-SN-DF** and **(*M*)-3-SN-DF**, respectively. DN gels ***rac*/(*P*)/(*M*)-3-DN-G** were obtained by swelling the corresponding dried films with chloroform or methanol.

Preparation of the Physically Doped Control Film

A solution of MeOEtMA (0.700 g, 4.86 mmol), K-SPMA (133 mg, 0.540 mmol), and PEGdMA (150 μL, 0.215 mmol) in NMF (3 mL) was degassed with nitrogen gas bubbling for 15 min and moved into a glovebox. Following the addition of the thermal initiator V-70 (8.3 mg, 2.7×10^{-2} mmol), the homogeneous solution was poured into a mold (ca. 30.0 mm × ca. 20.0 mm × 0.50 mm) assembled from two glass substrates and a silicone spacer (thickness = 0.50 mm), which was then allowed to stand at r.t. for 14 h. The obtained SN gel was washed three times by sequential solvent exchange with chloroform and methanol to remove unreacted species. The washed gels were dried under vacuum for over 3 h to afford SN dried films containing no mechanophores and emitters. Subsequently, the SN dried film was immersed in a solution of MeOEtMA (30.3 g, 0.210 mol), PEGdMA (292 μL, 0.420 mmol), and AIBN (69.0 mg, 0.420 mmol) in NMF (20 mL) at r.t. for 14 h in a glovebox. After swelling, the gel was removed from the solution, sandwiched between two glass plates, and placed on a hot plate. The second polymerization was initiated by heating to 50 °C and maintaining that temperature for 22 h. The resultant DN gel was washed three times by sequential solvent exchange with chloroform and methanol to remove unreacted species. The methanol-swollen DN gel was dried under vacuum overnight (for at least 12 h) to afford the DN dried elastomer film containing no mechanophores and emitters. Finally, the blank DN dried film was immersed in a chloroform solution of the rotaxane ***rac*-1** ($c = 1.0 \times 10^{-4}$ M) for 3 h. The physically doped gel was then dried under vacuum overnight (for at least 12 h) to afford the physically doped control film.

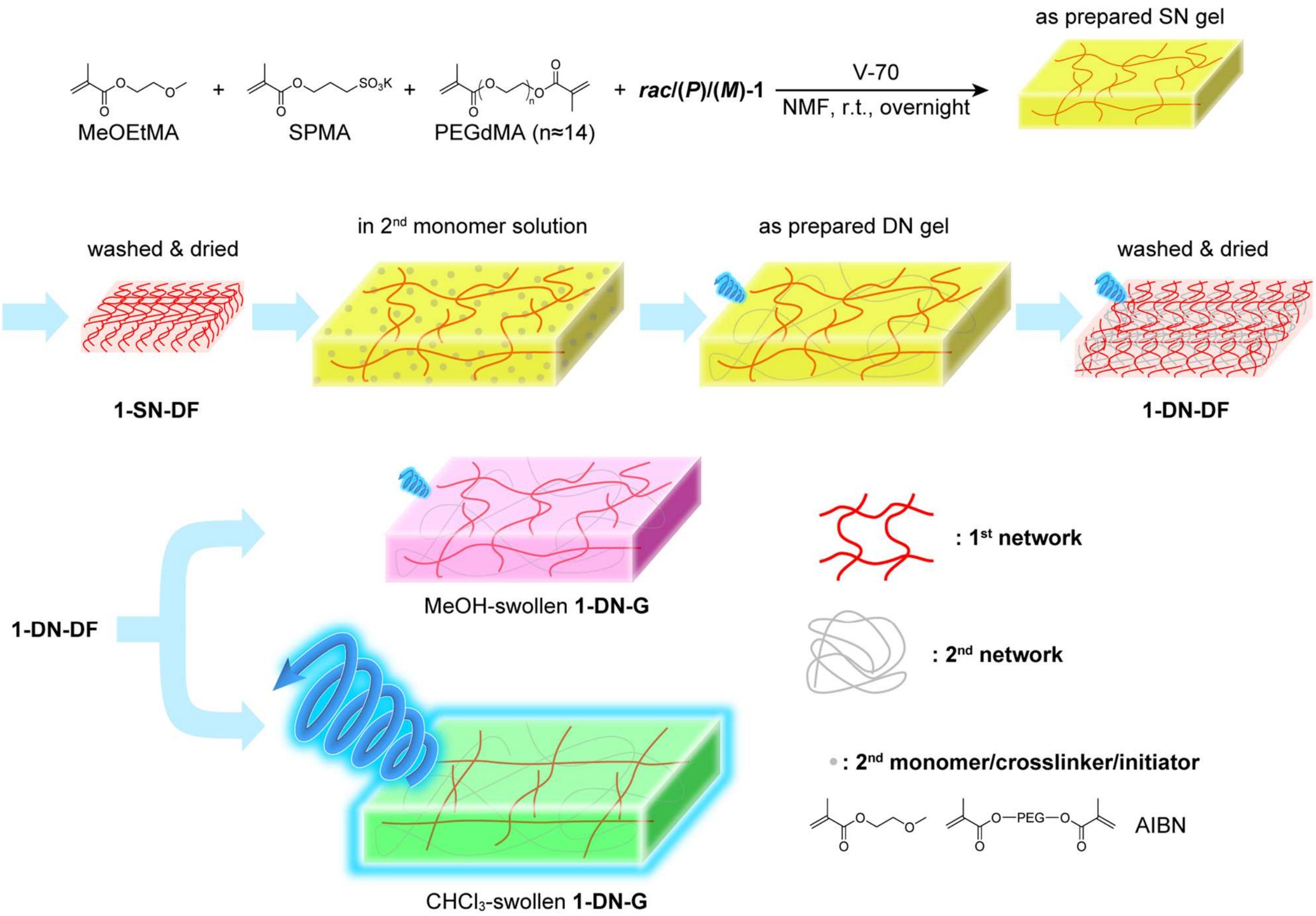

Figure S14. Preparation of rotaxane mechanophore-embedded dried films and gels.

Composition of DN Gels

Weight fraction of each component in gels

The weight fractions of the polymer and chloroform in each gel, ω_p and ω_{CHCl_3}, respectively, were calculated from the weights of the gel before and after swelling, W_{dry} and W_{wet}, respectively, using the following equations:

$$\omega_p = \frac{W_{dry}}{W_{wet}}$$

$$\omega_{CHCl_3} = 1 - \omega_p$$

The polymer weight fraction of DN gel, $\omega_{p,DN}$, is the sum of the weight fractions of the first and second network. The weight fractions of the first network and second network of the DN gel, $\omega_{1,DN}$ and $\omega_{2,DN}$, respectively, were calculated approximately as follows:

$$\omega_{1,DN} = \omega_{p,SN}\left(\frac{t_{SN}}{t_{DN}}\right)^3$$

$$\omega_{2,DN} = \omega_{p,DN} - \omega_{1,DN}$$

, where $\omega_{p,SN}$ is the polymer weight fraction of **1-SN-G** and t_{SN} and t_{DN} are the thicknesses of the swollen SN gel and the DN gel, respectively. Table S1 summarizes the weight fraction of each component of the SN and DN gels.

Table S1. Composition of chloroform-swollen gels.

Sample code	First network	Second network	Solvent
	Weight fraction of first-network ω_1 (wt%)	Weight fraction of second-network ω_2 (wt%)	Weight fraction of chloroform ω_{CHCl_3} (wt%)
***rac*-1-SN-G**	12.0	-	88.1
**(*P*)-1-SN-G**	12.4	-	87.6
**(*M*)-1-SN-G**	13.0	-	87.0
***rac*-1-DN-G**	2.30	11.1	86.6
**(*P*)-1-DN-G**	2.23	11.7	86.0
**(*M*)-1-DN-G**	1.63	11.7	86.7

Table S2. Composition of monomer feeds for DN gels.

First network			Second network	
Monomer concentration C_1 (M)	Molar percentage of cross-linker x_1 (mol%) (PEGdMA)	Molar percentage of rotaxane x_{Rot} (mol%)	Monomer concentration C_2 (M)	Molar percentage of cross-linker x_2 (mol%) (PEGdMA)
0.54	4.0	3.7×10^{-4}	10.5	0.200

Optical Properties of DN Dried Films

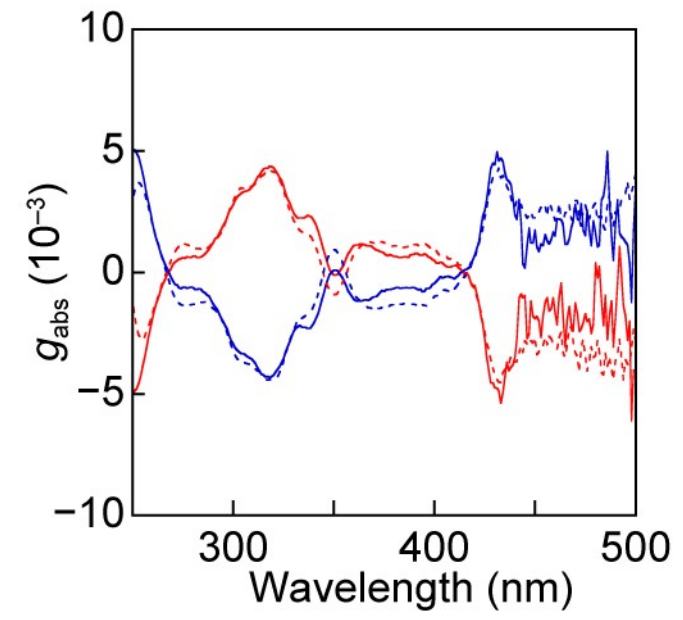

Figure S15. Mirror-image g_{abs} spectra of **(*P*)-1** (red solid line) in chloroform ($c = 1.0 \times 10^{-5}$ M), **(*M*)-1** (blue solid line) in chloroform ($c = 1.0 \times 10^{-5}$ M), **(*P*)-1-DN-DF** (red dotted line), and **(*M*)-1-DN-DF** (blue dotted line).

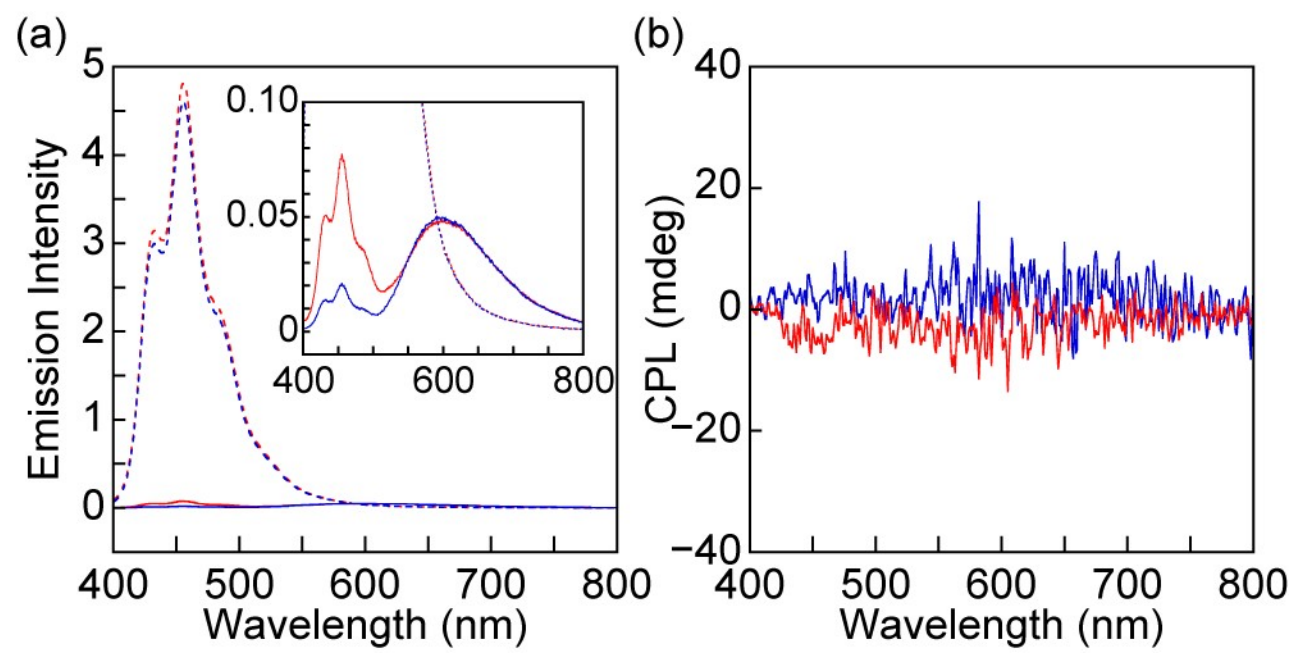

Figure S16. (a) Photoluminescence spectra of **(*P*)-1-DN-DF** (red solid line), **(*M*)-1-DN-DF** (blue solid line), **(*P*)-3-DN-DF** (red dotted line), and **(*M*)-3-DN-DF** (blue dotted line). λ_{ex} = 365 nm. (b) CPL spectra of **(*P*)-1-DN-DF** (red) and **(*M*)-1-DN-DF** (blue). λ_{ex} = 330 nm.

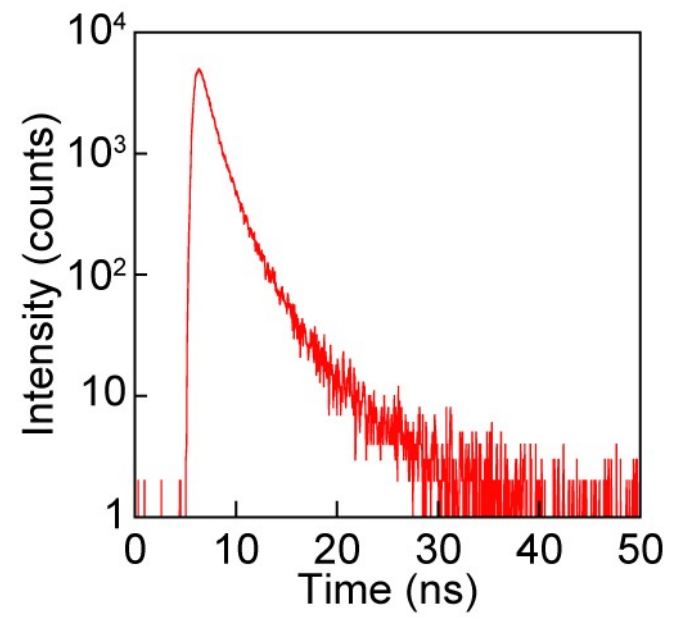

Figure S17. Emission decay profile of ***rac*-1-DN-DF**. The emission decay was recorded with excitation light of 330 nm and monitored at 600 nm.

Stability Test of Rotaxane Under Radical Polymerization Conditions

Treatment of *rac*-2 under First Network Formation Conditions

A solution of MeOEtMA (0.28 g, 1.9 mmol), K-SPMA (53 mg, 0.22 mmol), PEGdMA (60 µL, 8.6×10^{-2} mmol), and ***rac*-2** (2.0 mg, 0.85 µmol) in NMF (1.2 mL) was degassed with nitrogen gas bubbling for 15 min and moved into a glovebox. Following the addition of the thermal initiator V-70 (3.3 mg, 1.1×10^{-2} mmol), the homogeneous solution was poured into a mold (ca. 30.0 mm × ca. 20.0 mm × 0.50 mm) assembled from two glass substrates and a silicone spacer (thickness = 0.50 mm), which was left at r.t. for 14 h. The obtained polymer network was washed three times by sequential solvent exchange with chloroform and methanol to extract the embedded ***rac*-2**. The combined washing solutions were collected and evaporated under reduced pressure at r.t. The residue was dissolved in chloroform.

Treatment of *rac*-2 under Second Network Formation Conditions

A solution of MeOEtMA (6.1 g, 4.2×10^{-2} mol), PEGdMA (58 µL, 8.4×10^{-2} mmol), ***rac*-2** (2.0 mg, 0.85 µmol), and the thermal initiator AIBN (13.8 mg, 8.4×10^{-2} mmol) in NMF (4 mL) was degassed with nitrogen gas bubbling for 15 min and moved into a glovebox. The homogeneous solution was poured into a stainless steel mold and heated to 50 °C and maintained at that temperature for 20 h. The obtained polymer network was washed three times by sequential solvent exchange with chloroform and methanol to extract the embedded ***rac*-2**. The combined washing solutions were collected and evaporated under reduced pressure at r.t. The residue was dissolved in chloroform.

Spectroscopic Analysis

The absorbance of the collected ***rac*-2** solutions in chloroform was adjusted to match that of a pristine ***rac*-2** solution ($c = 1.0 \times 10^{-5}$ M) (Figure S18a). The fluorescence spectra of the absorbance-matched samples were then recorded (Figure S18b). In both cases, the fluorescence intensity remained negligible, confirming that the rotaxane moiety is chemically stable under the radical polymerization conditions employed for both network formations.

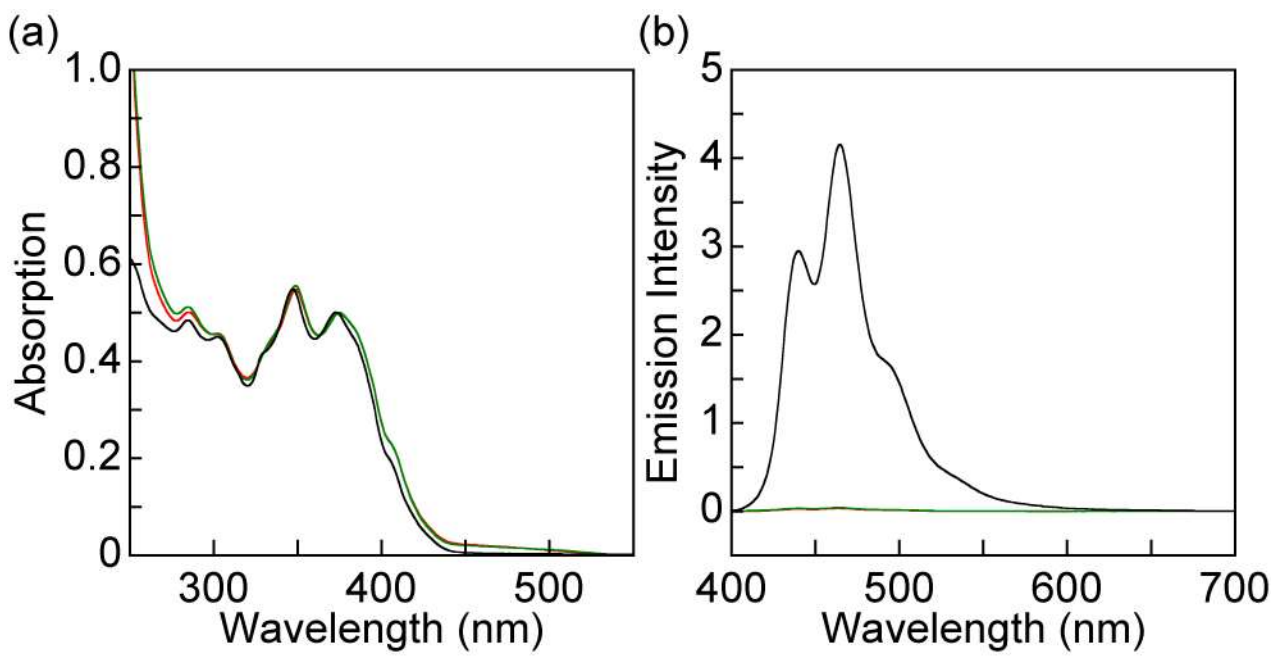

Figure S18. (a) Absorption and (b) photoluminescence spectra of ***rac*-3** in chloroform (black, $c = 1.0 \times 10^{-5}$ M), ***rac*-2** with unreacted species after first network polymerization (red), and ***rac*-2** with unreacted species after second network polymerization (green). $\lambda_{ex} = 330$ nm.

Optical Properties of DN Gels

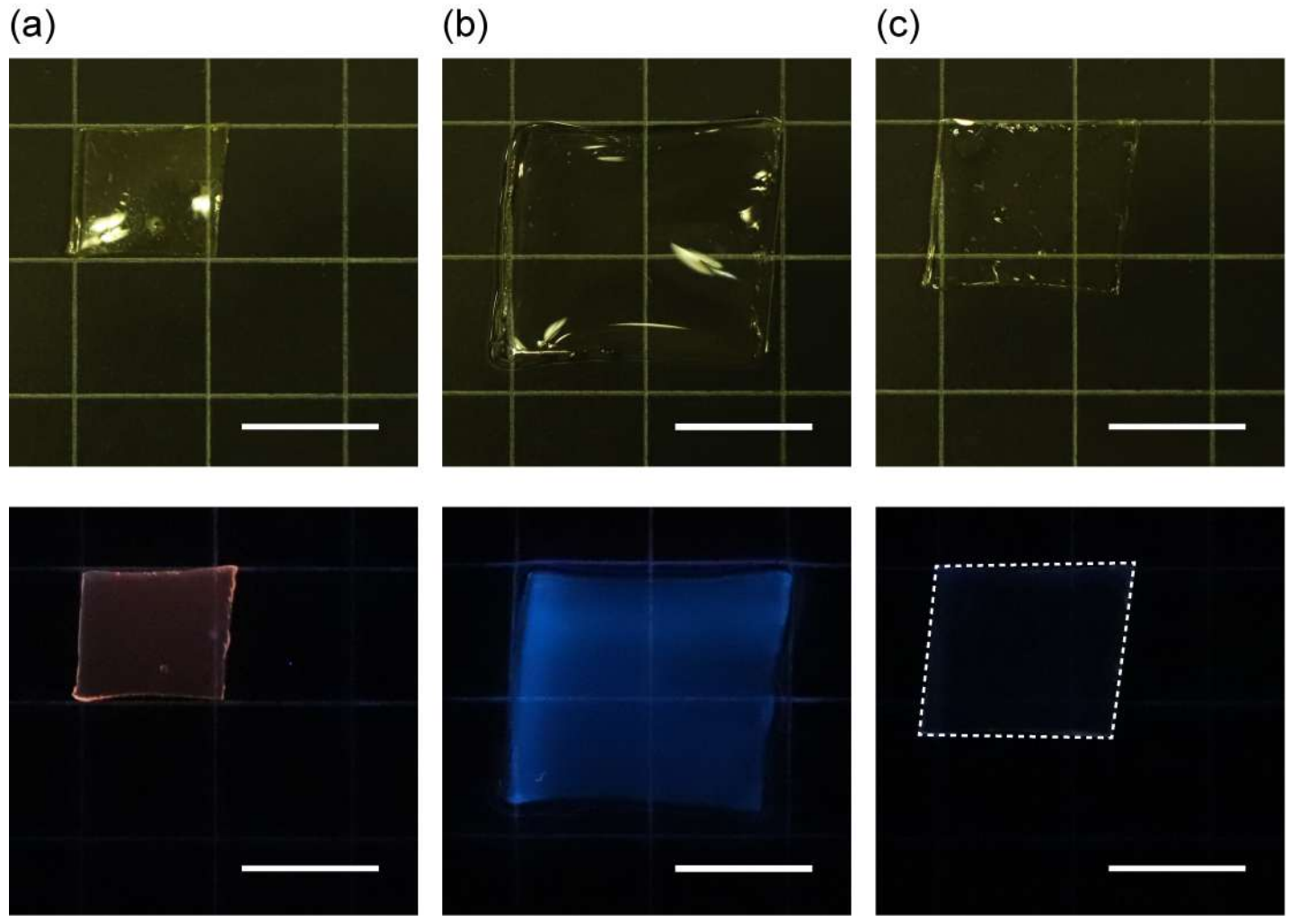

Figure S19. Photographs of (a) ***rac*-1-DN-DF**, (b) chloroform-swollen ***rac*-1-DN-G**, and (c) methanol-swollen ***rac*-1-DN-G** films. The white dotted line represents the position of the methanol-swollen ***rac*-1-DN-G** film. Top and bottom images were taken under ambient condition and irradiation with 365 nm light, respectively. Scale bar: 1 cm.

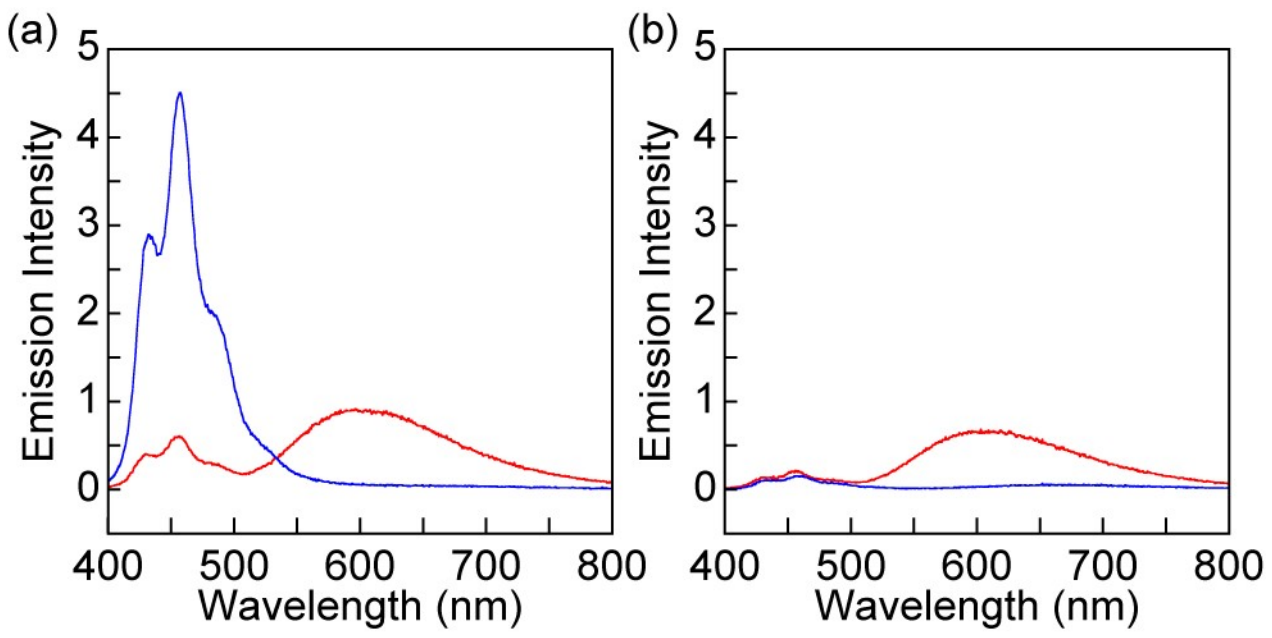

Figure S20. (a) Photoluminescence spectra of ***rac*-1-DN-DF** (red) and chloroform-swollen ***rac*-1-DN-G** (blue). (b) Photoluminescence spectra of ***rac*-1-SN-DF** (red) and chloroform-swollen ***rac*-1-SN-G** (blue). λ_{ex} = 365 nm. These spectra were recorded with narrower emission slit width.

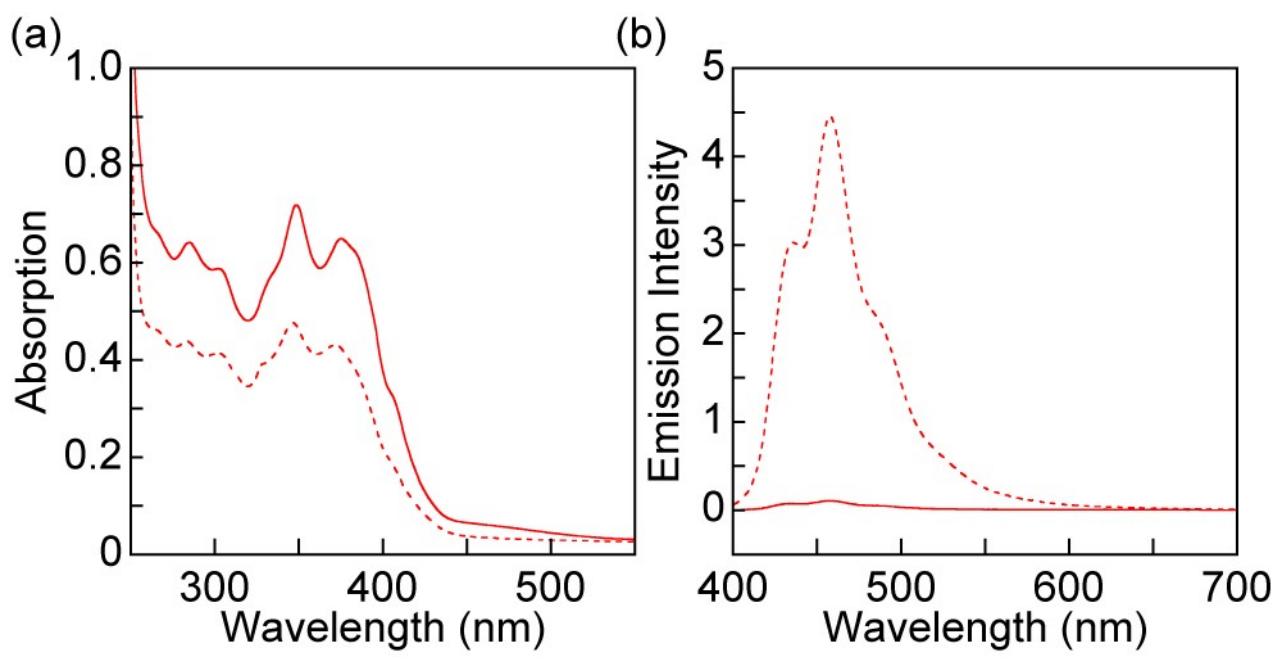

Figure S21. (a) Absorption and (b) photoluminescence spectra of methanol-swollen ***rac*-1-DN-G** (solid) and ***rac*-3-DN-G** (dotted). λ_{ex} = 330 nm.

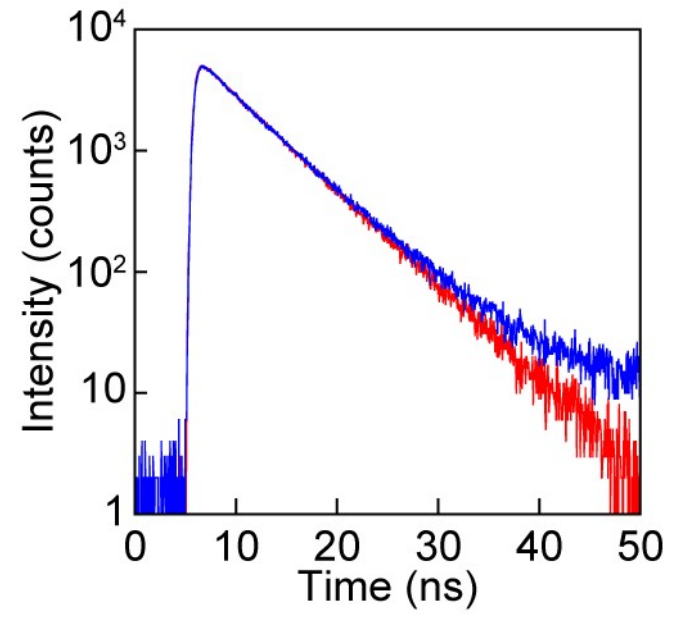

Figure S22. Emission decay profiles of ***rac*-3** in chloroform (red) and chloroform-swollen ***rac*-1-DN-G** (blue). The emission decays were recorded with excitation light of 330 nm and monitored at 460 nm.

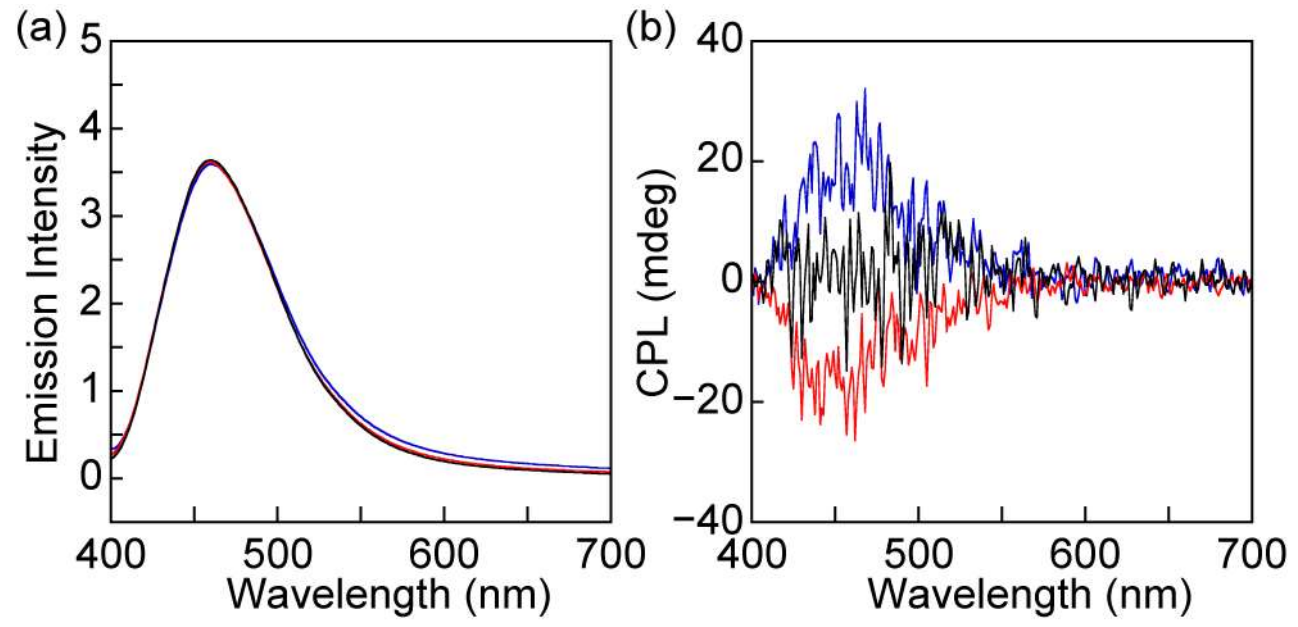

Figure S23. (a) Photoluminescence and (b) CPL spectra of chloroform-swollen **(*P*)-1-DN-G** (red), **(*M*)-1-DN-G** (blue), and ***rac*-1-DN-G** (black). λ_{ex} = 330 nm.

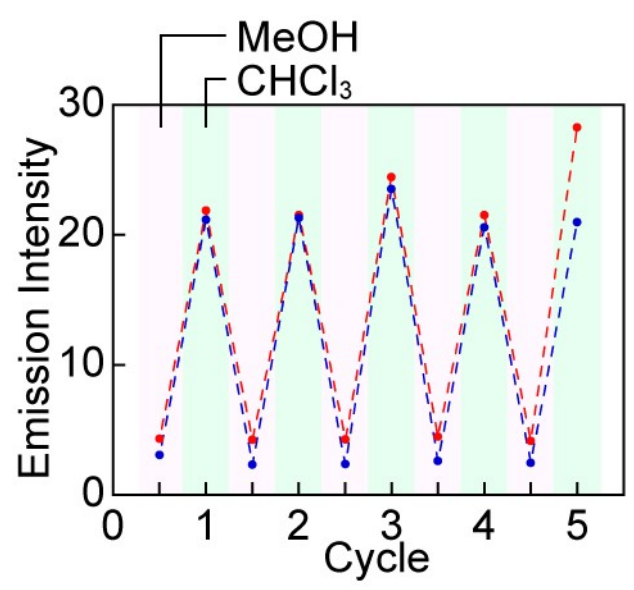

Figure S24. Plots of the photoluminescence intensity at 460 nm of **(*P*)-1-DN-G** (red dots) and **(*M*)-1-DN-G** (blue dots) upon sequential solvent exchanges between methanol and chloroform over five-cycles. λ_{ex} = 330 nm.

Mechanical Properties of DN Dried Elastomer Films and Gels

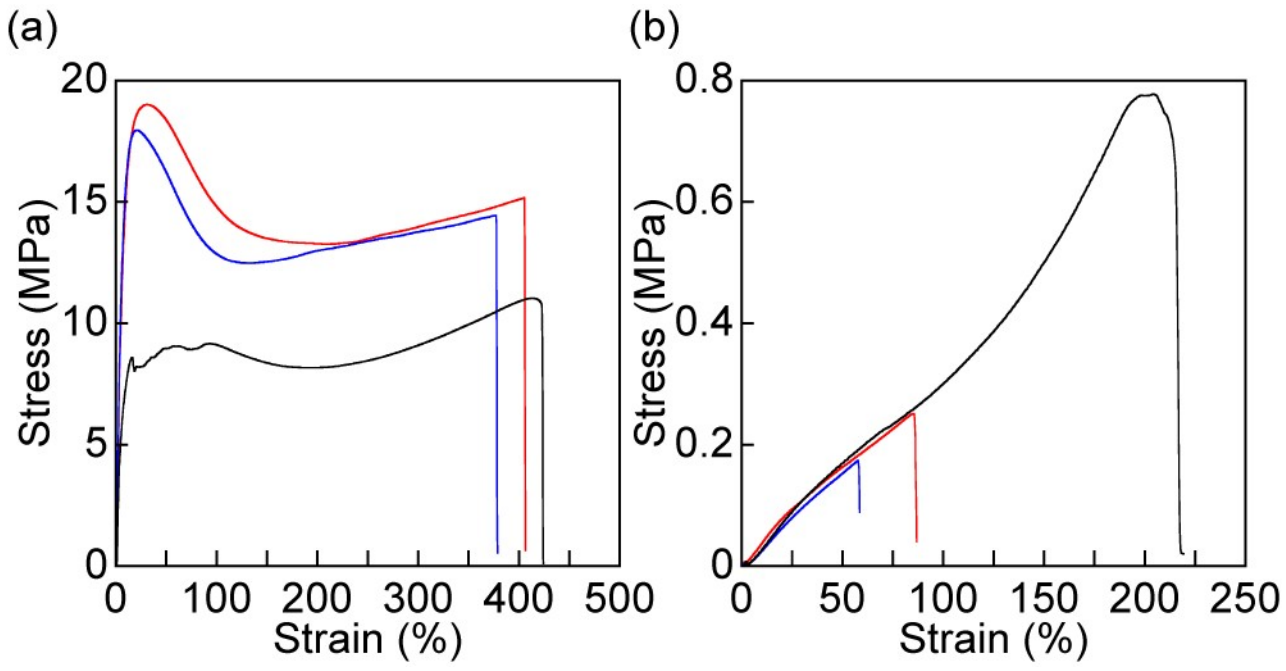

Figure S25. (a) Stress-strain curves of the ***(P)*-1-DN-DF** (red), ***(M)*-1-DN-DF** (blue), and ***rac*-1-DN-DF** (black) films. (b) Stress-strain curves of the methanol-swollen ***(P)*-1-DN-G** (red), ***(M)*-1-DN-G** (blue), and ***rac*-1-DN-G** (black) films.

Table S3. Overview of the mechanical properties of **1-DN-DF** and **1-DN-G** films as determined from stress-strain curves recorded during uniaxial tensile deformation (see Figure S25).

Sample code	Elongation at break (%)	Stress at break (MPa)	Young's modulus (MPa)[b]
**(*P*)-1-DN-G**[a]	85.1	0.25	4.41×10^{-3}
***rac*-1-DN-G**[a]	204.5	0.78	4.05×10^{-3}
**(*M*)-1-DN-G**[a]	57.6	0.17	3.72×10^{-3}
**(*P*)-1-DN-E**	405.3	15.16	3.42
***rac*-1-DN-E**	413.3	11.04	1.77
**(*M*)-1-DN-E**	377.3	14.44	3.72

[a] All **1-DN-G** were swollen with methanol. [b] The Young's moduli were derived from the slopes of the stress-strain curves in the strain regime between 1.5–2.5% for **1-DN-DF**, 5–15% for **1-DN-G**.

Changes in Photoluminescence Spectra of a DN Dried Film Physically Doped with *rac*-1 upon Stretching

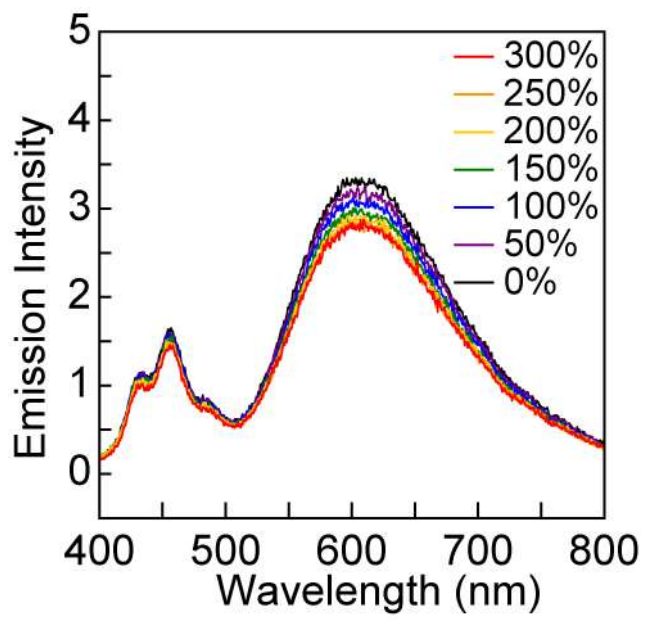

Figure S26. Photoluminescence spectra of a DN dried film physically doped with the rotaxane ***rac*-1** upon uniaxial deformation to the indicated strains. λ_{ex} = 365 nm.

CPL Measurements of Stretched (*P*)-1-DN-DF

As shown in Figure S27, the **(*P*)-1-DN-DF** film uniaxially stretched to 260% strain was fixed on a glass substrate to maintain the deformed state. CPL measurements were performed on the sample while rotating it in the plane perpendicular to the excitation light. The spectra were recorded at rotation angle increments of 10°, and the luminescence dissymmetry factor (g_{lum}) was calculated for each angle.

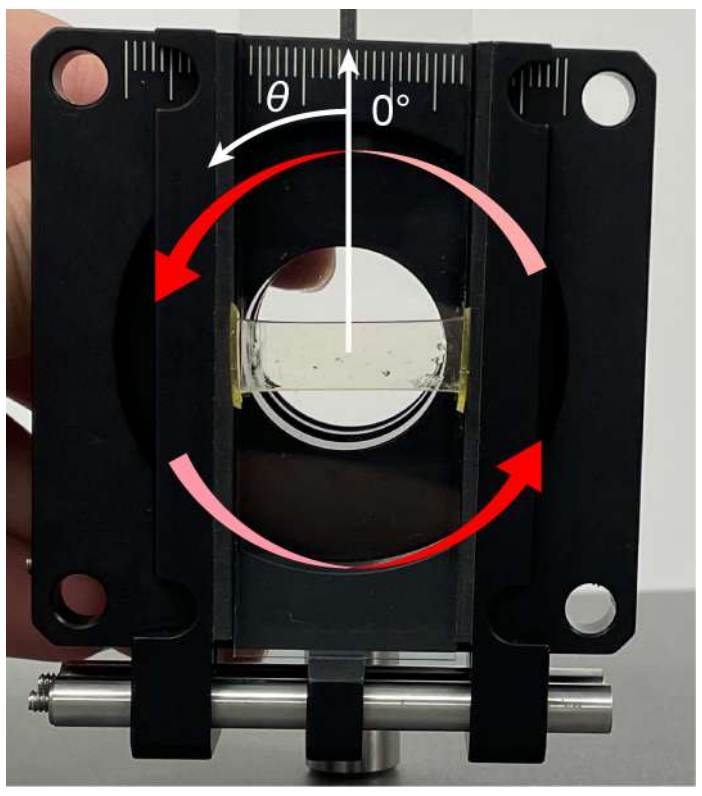

Figure S27. Experimental setup for CPL measurements of the uniaxially stretched **(*P*)-1-DN-DF** film. The film was stretched to 260% strain. The sample rotation angle θ is defined as the angle between the stretching direction (elongation axis) and the vertical axis of the instrument.

NMR Spectra of Organic Compounds

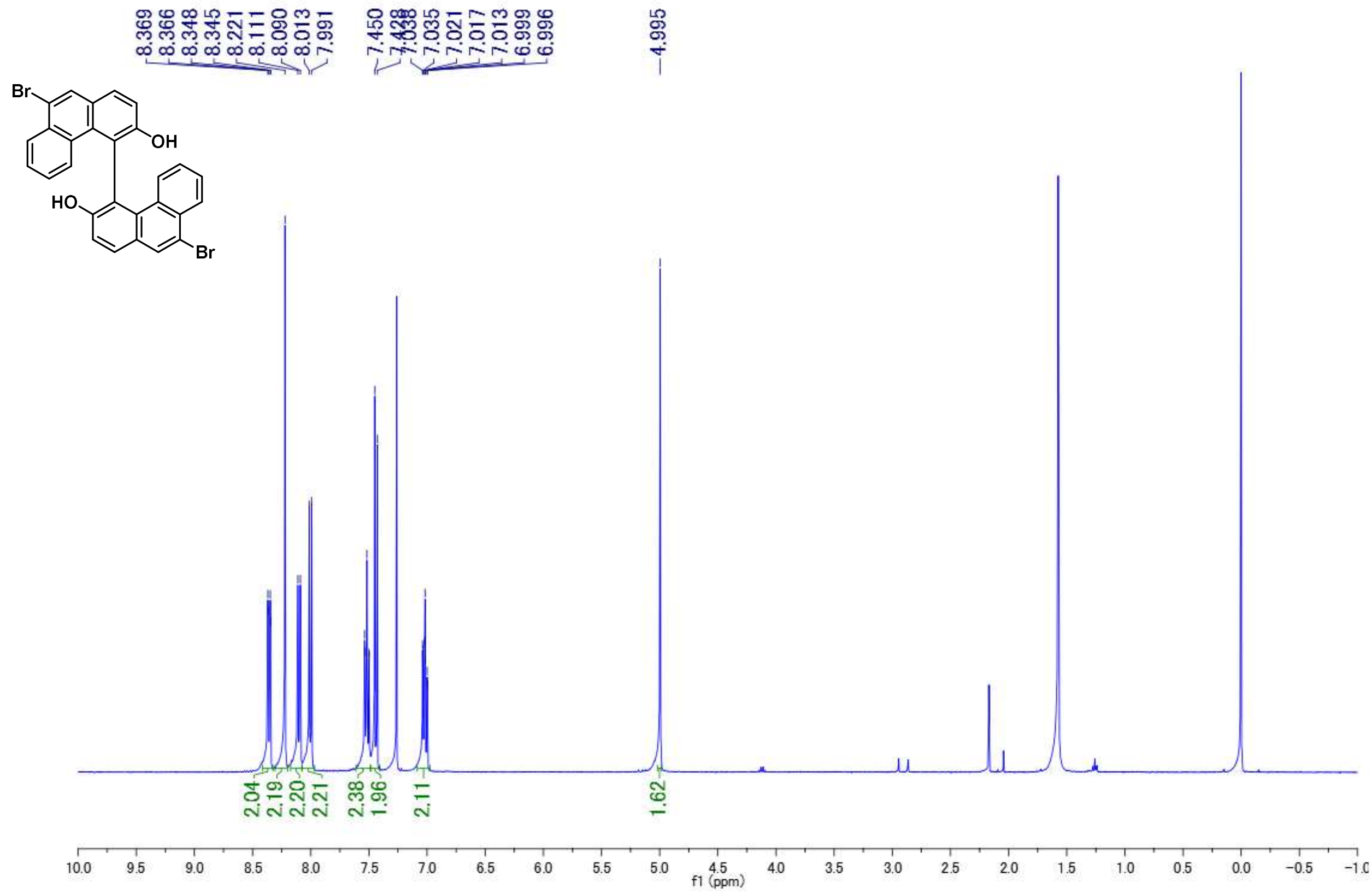

^{1}H NMR (400 MHz, $CDCl_3$) spectrum of compound **5**

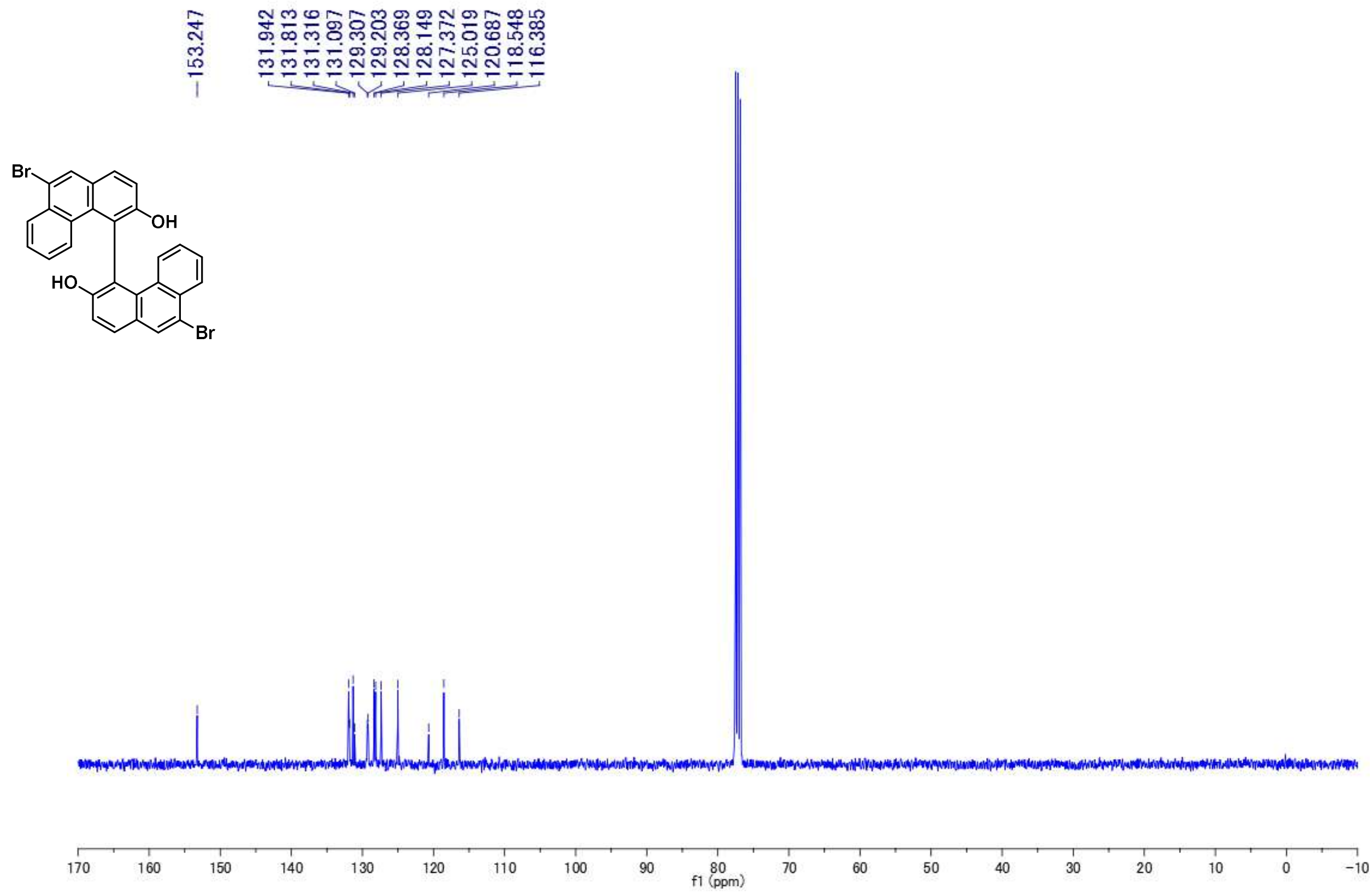

^{13}C NMR (100 MHz, $CDCl_3$) spectrum of compound **5**

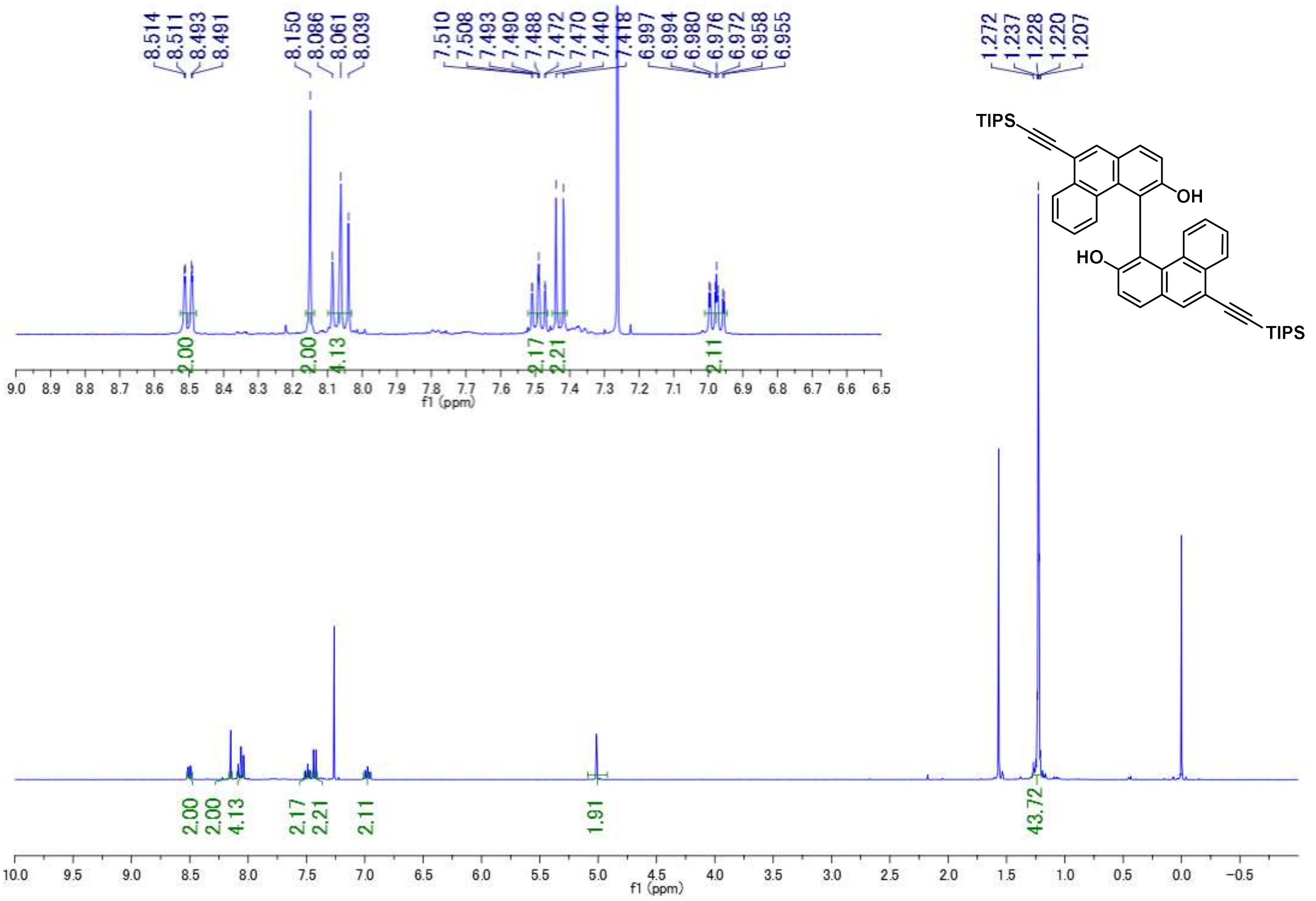

^{1}H NMR (400 MHz, $CDCl_3$) spectrum of compound **6**

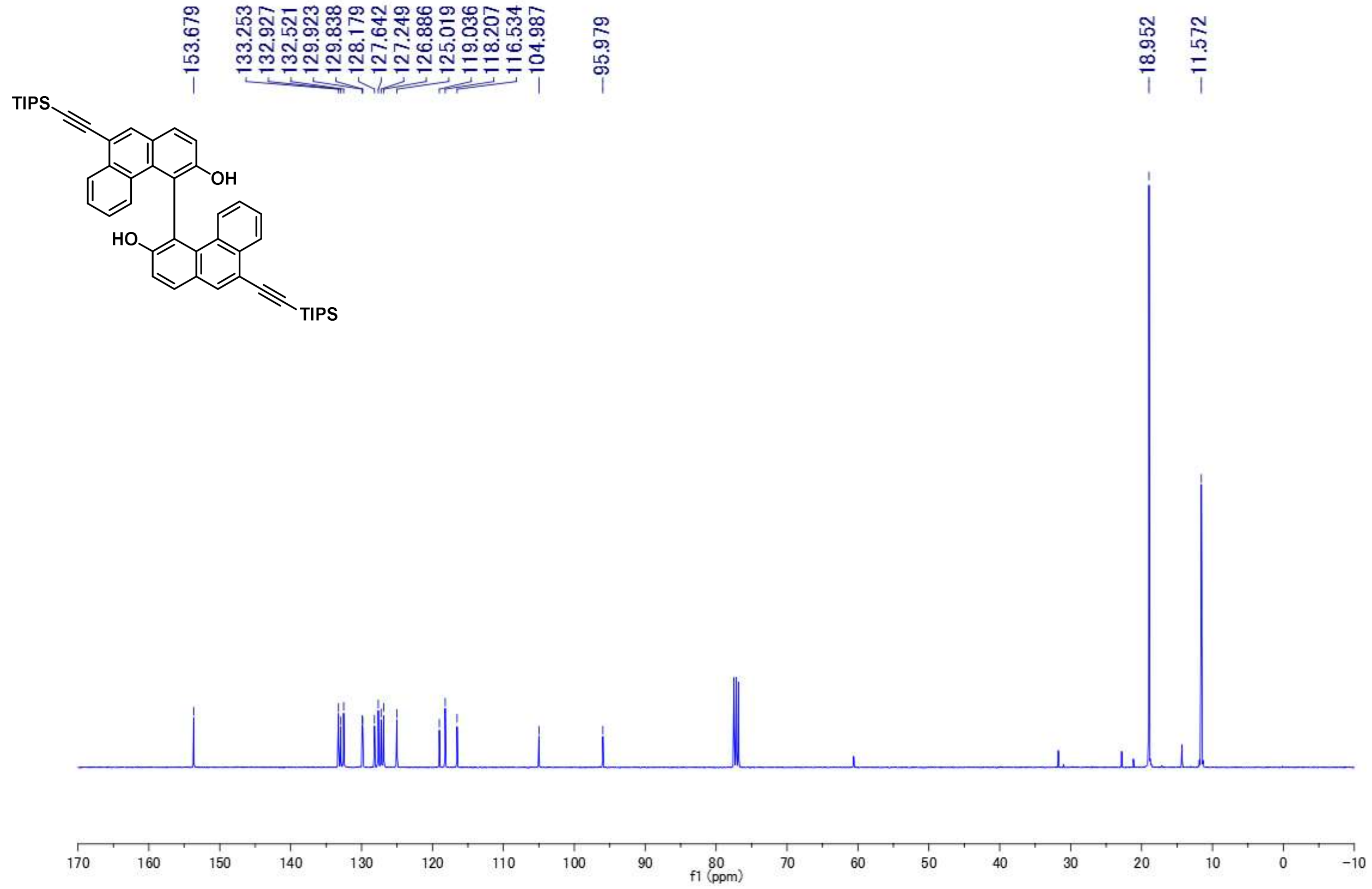

^{13}C NMR (100 MHz, $CDCl_3$) spectrum of compound **6**

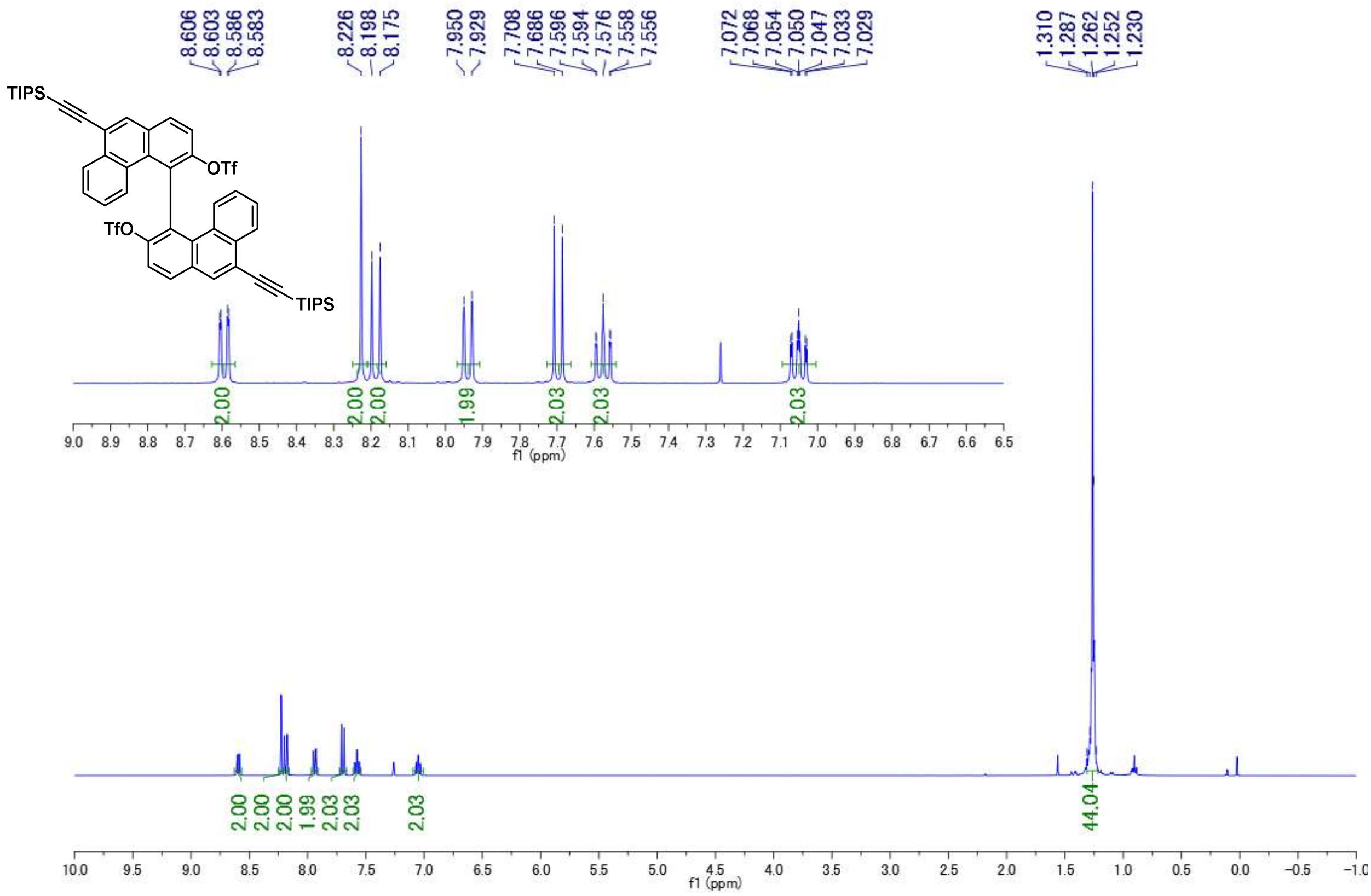

^{1}H NMR (400 MHz, $CDCl_3$) spectrum of compound **7**

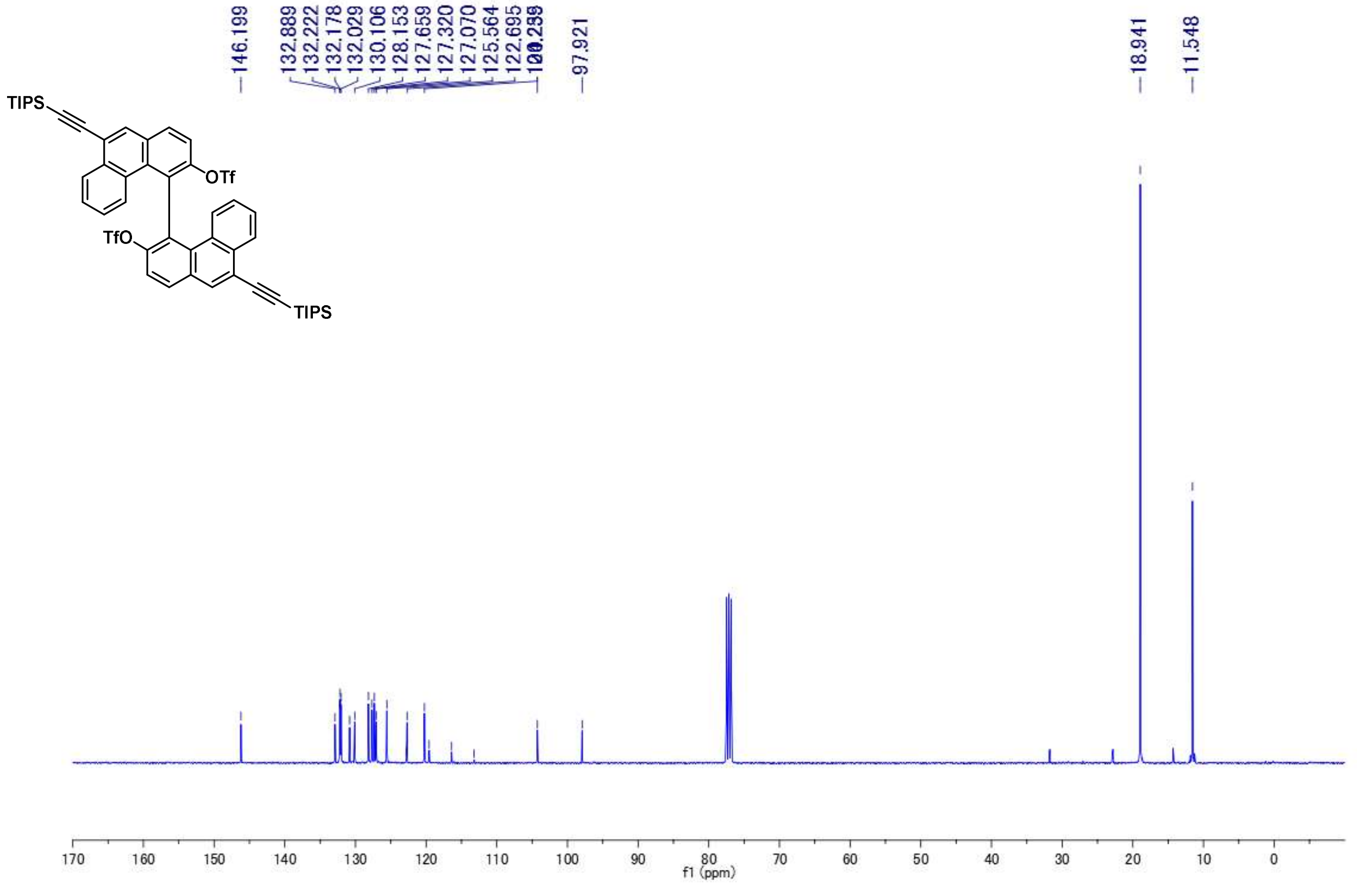

^{13}C NMR (100 MHz, $CDCl_3$) spectrum of compound **7**

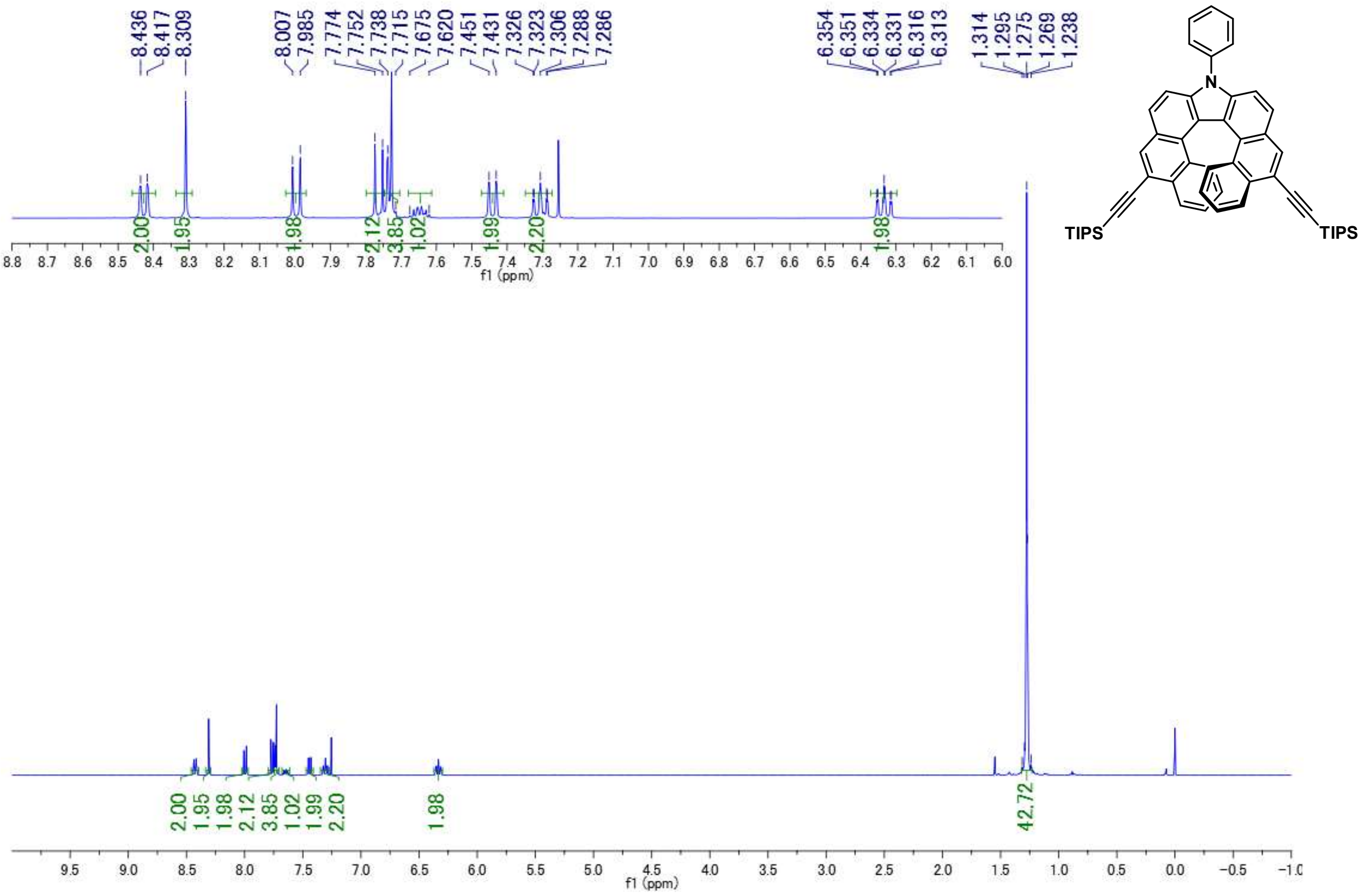

^{1}H NMR (400 MHz, $CDCl_3$) spectrum of compound **8**

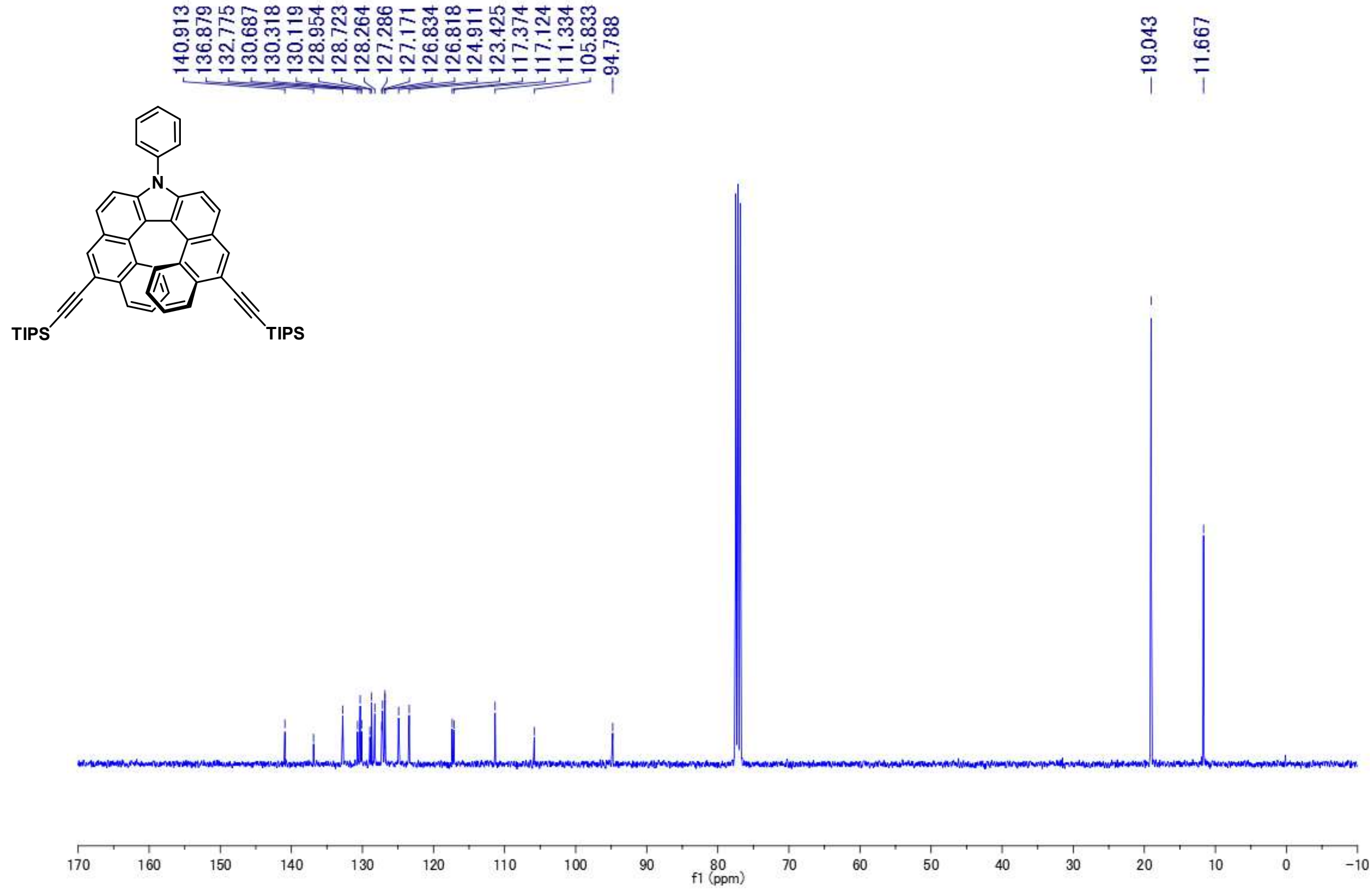

^{13}C NMR (100 MHz, $CDCl_3$) spectrum of compound **8**

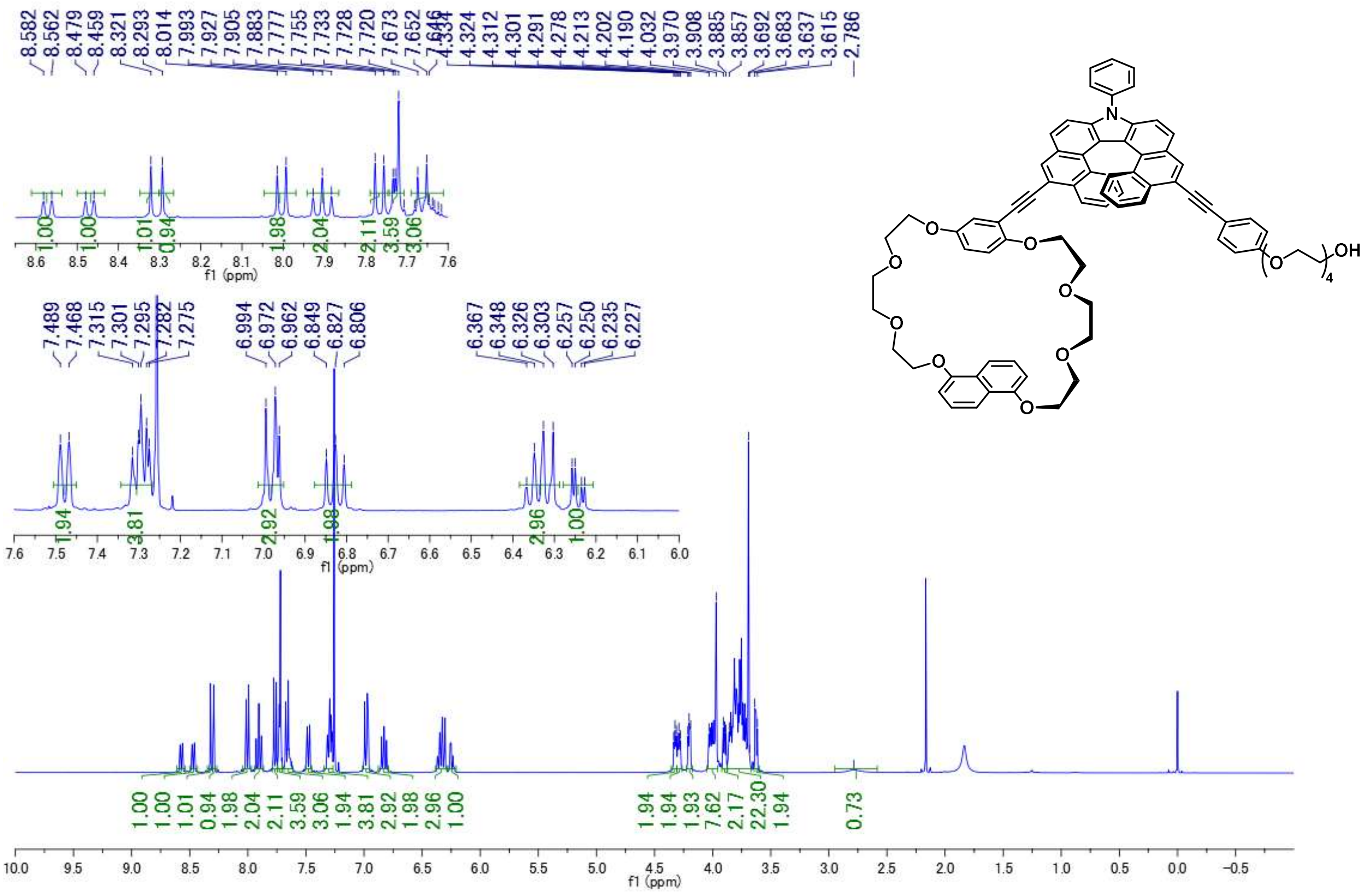

^{1}H NMR (400 MHz, $CDCl_3$) spectrum of compound ***rac*-4**

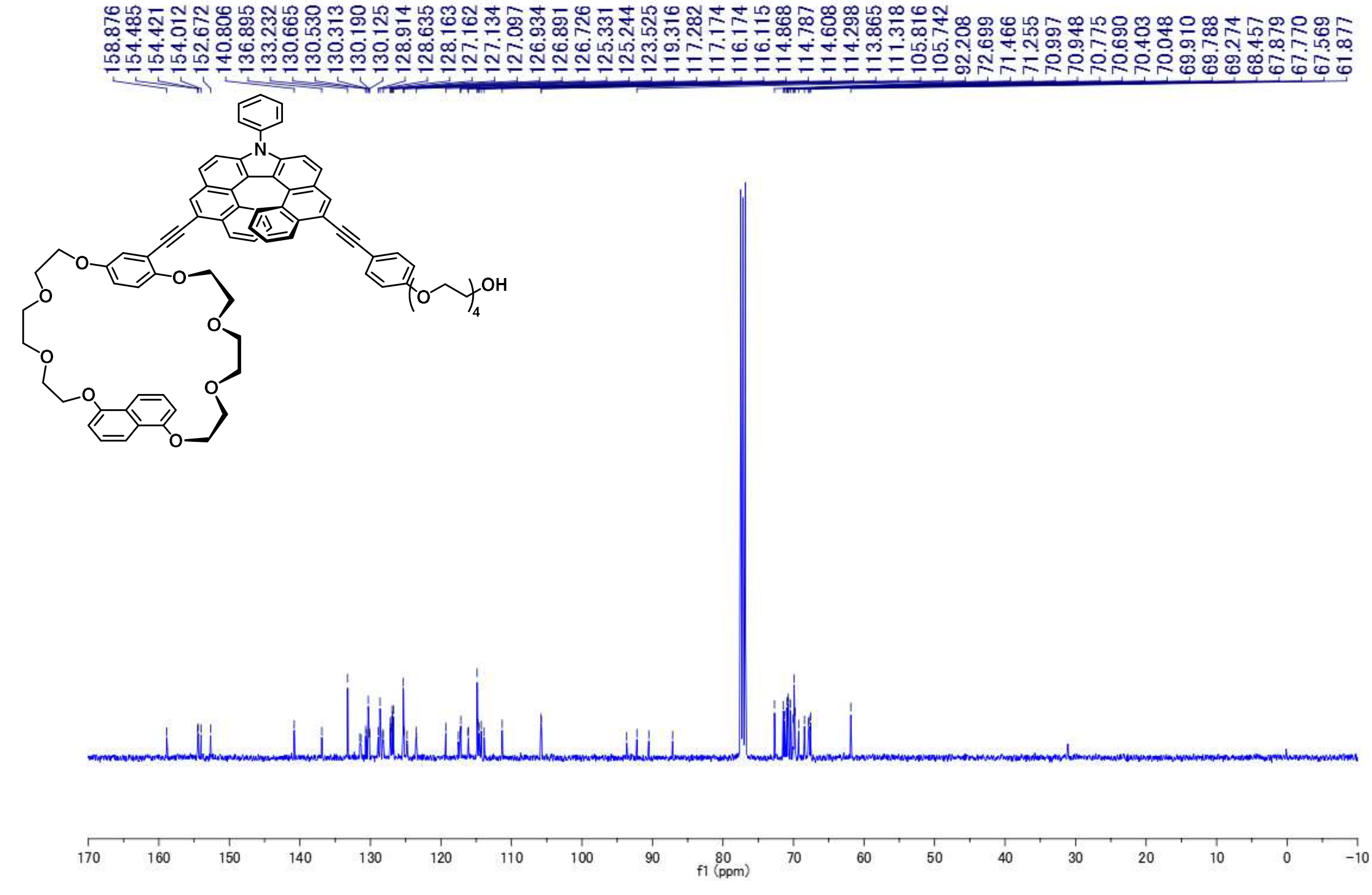

^{13}C NMR (100 MHz, $CDCl_3$) spectrum of compound ***rac*-4**

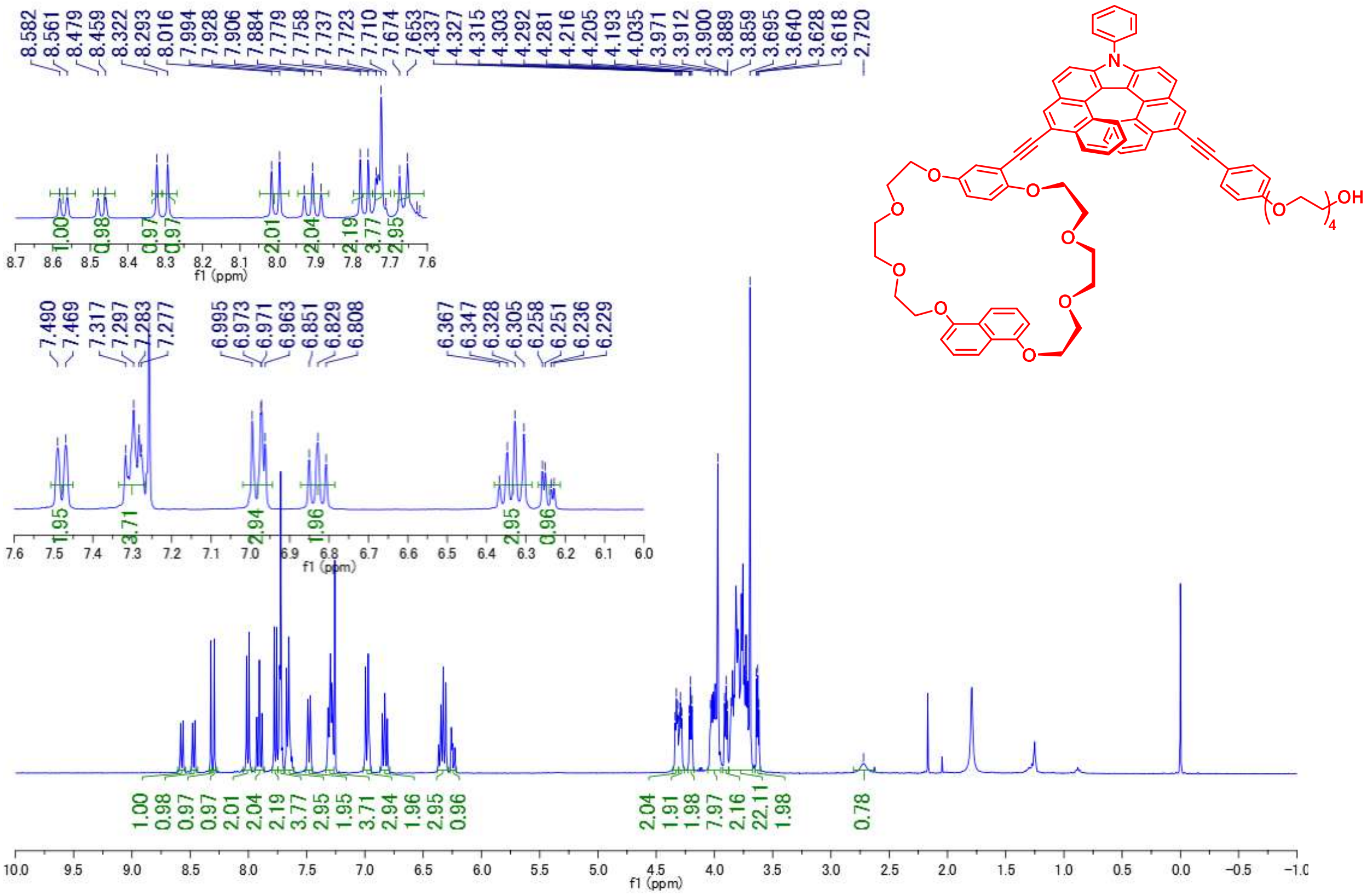

^{1}H NMR (400 MHz, $CDCl_3$) spectrum of compound **(*P*)-4**

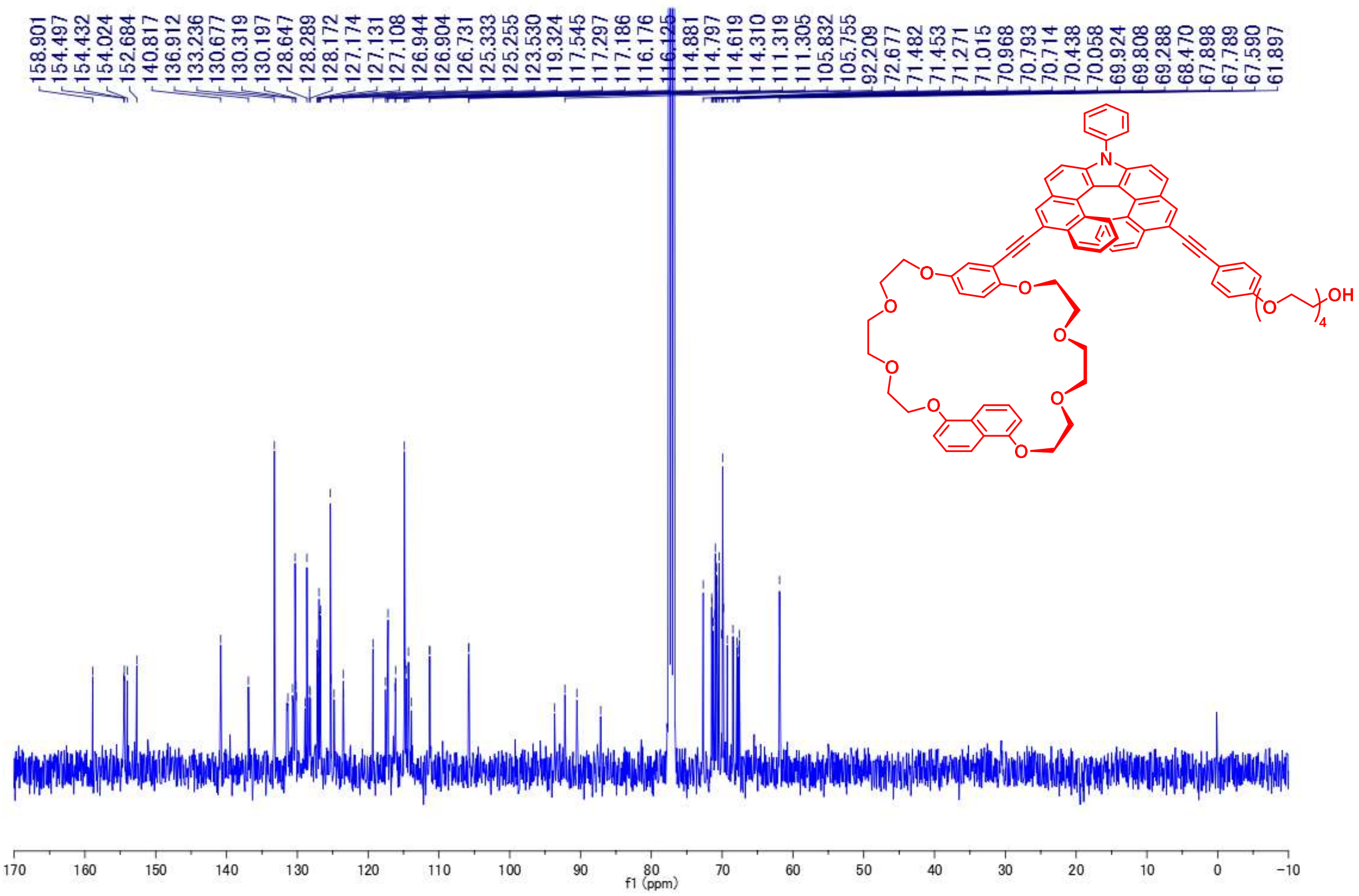

^{13}C NMR (100 MHz, $CDCl_3$) spectrum of compound **(*P*)-4**

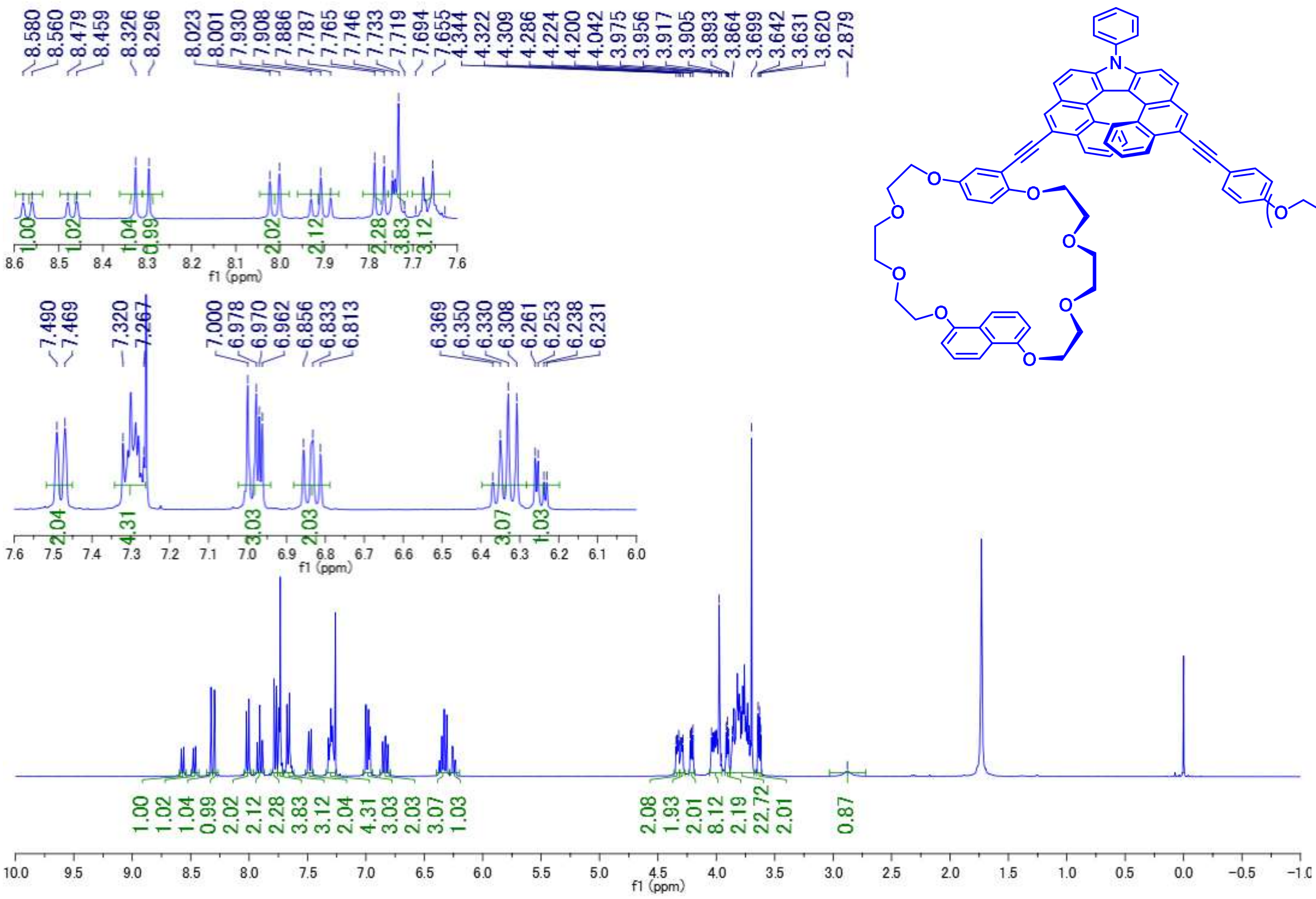

^{1}H NMR (400 MHz, $CDCl_3$) spectrum of compound **(*M*)-4**

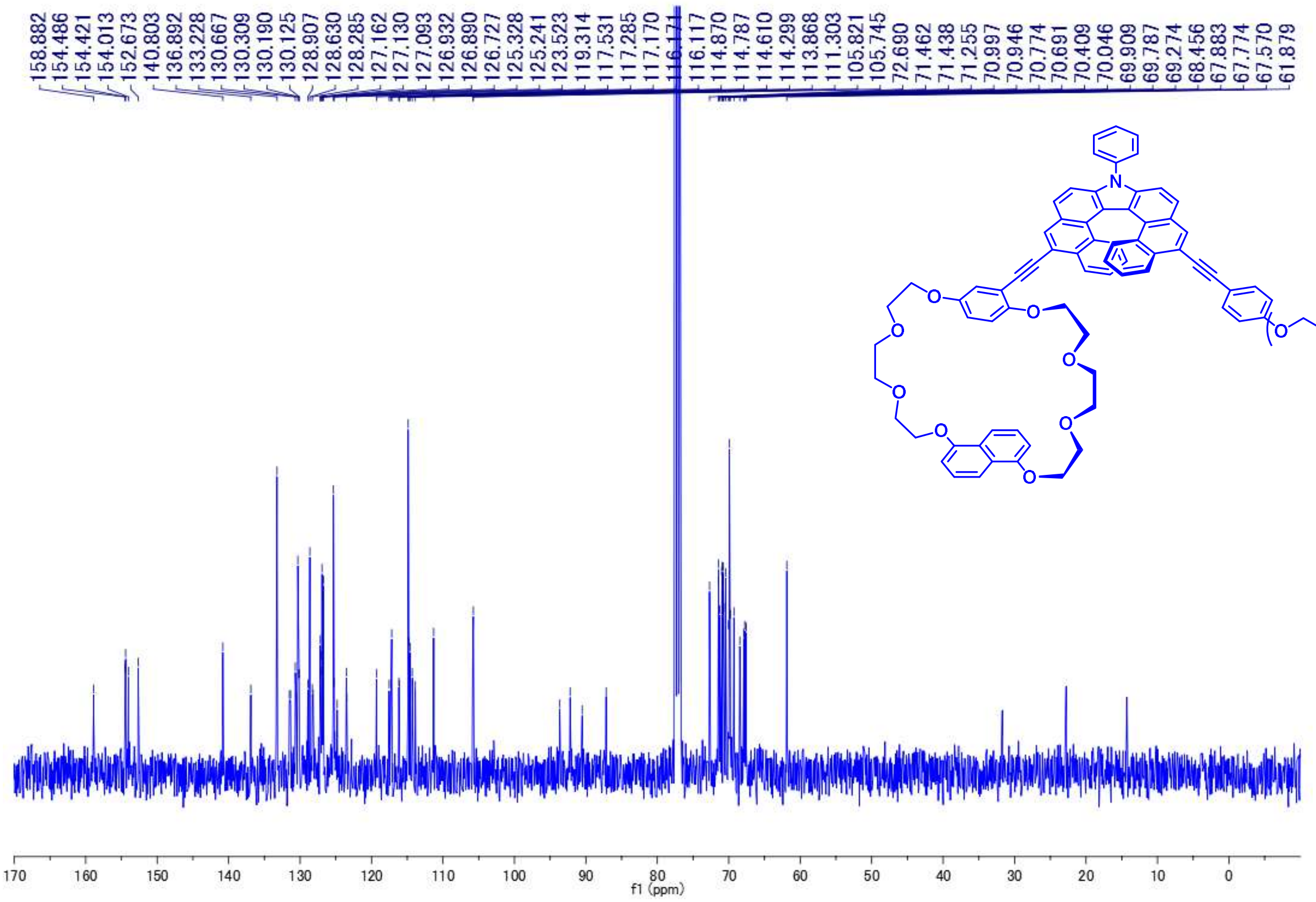

^{13}C NMR (100 MHz, $CDCl_3$) spectrum of compound **(*M*)-4**

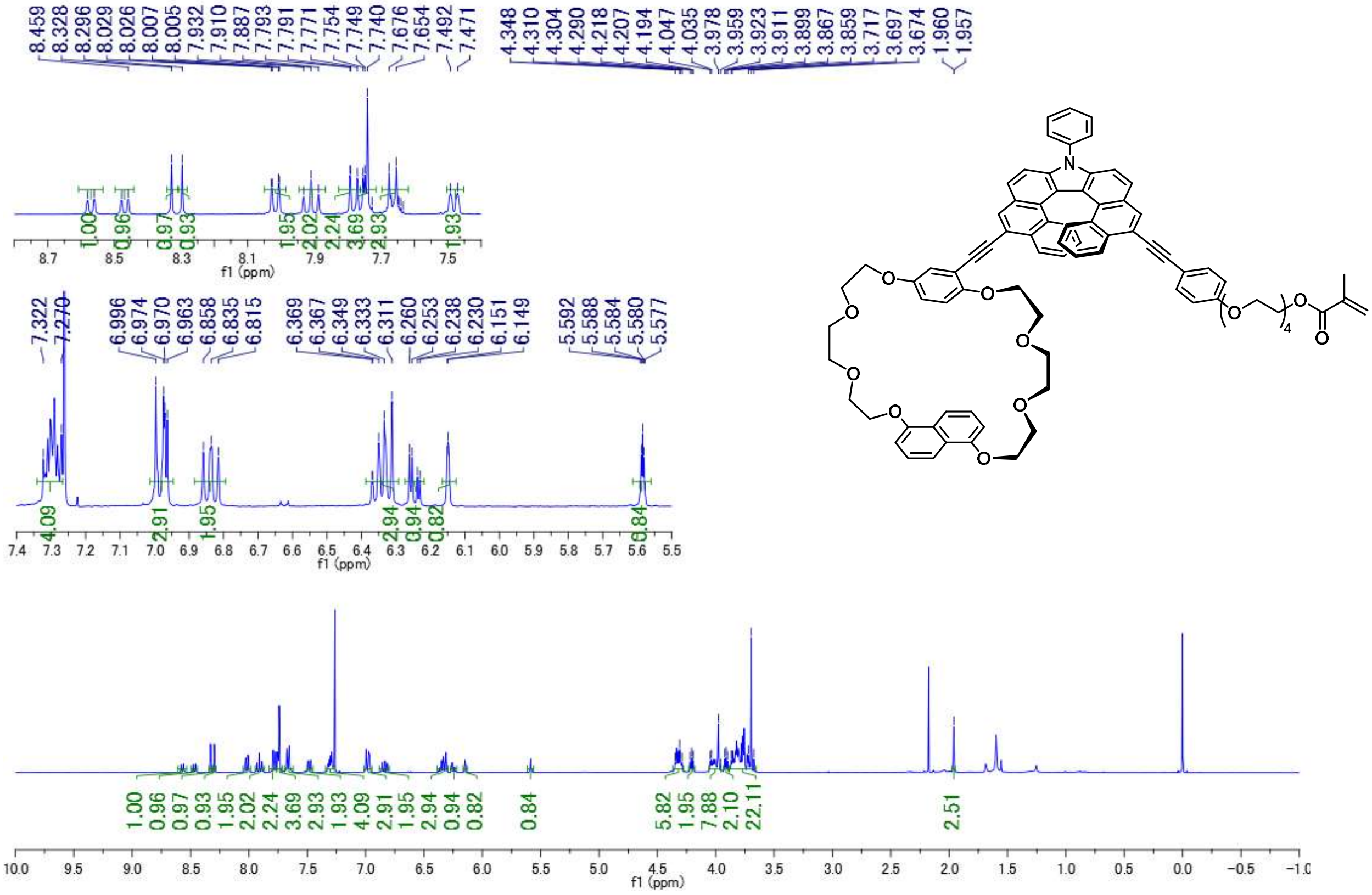

^{1}H NMR (400 MHz, $CDCl_3$) spectrum of compound ***rac*-3**

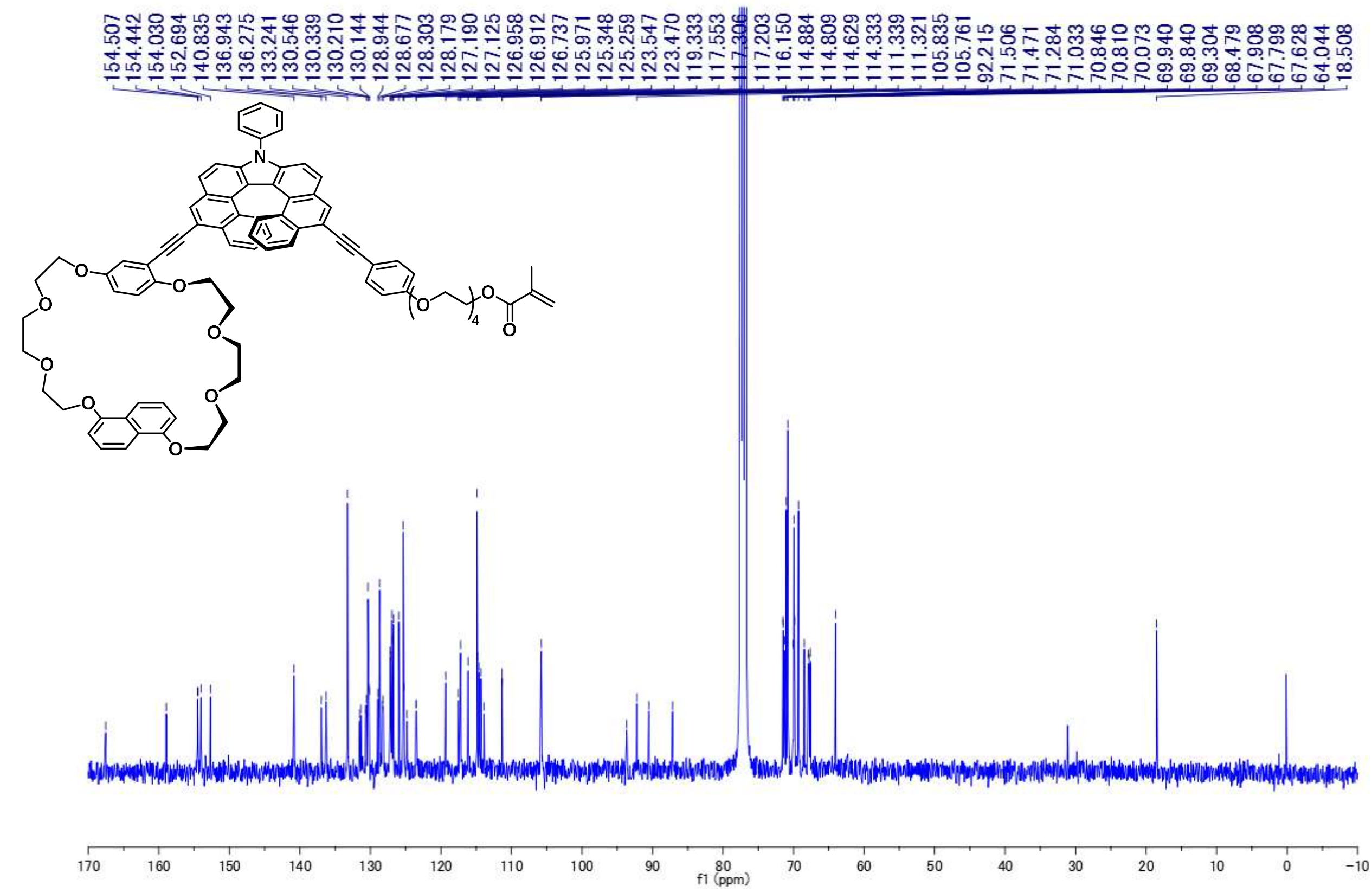

^{13}C NMR (100 MHz, $CDCl_3$) spectrum of compound ***rac*-3**

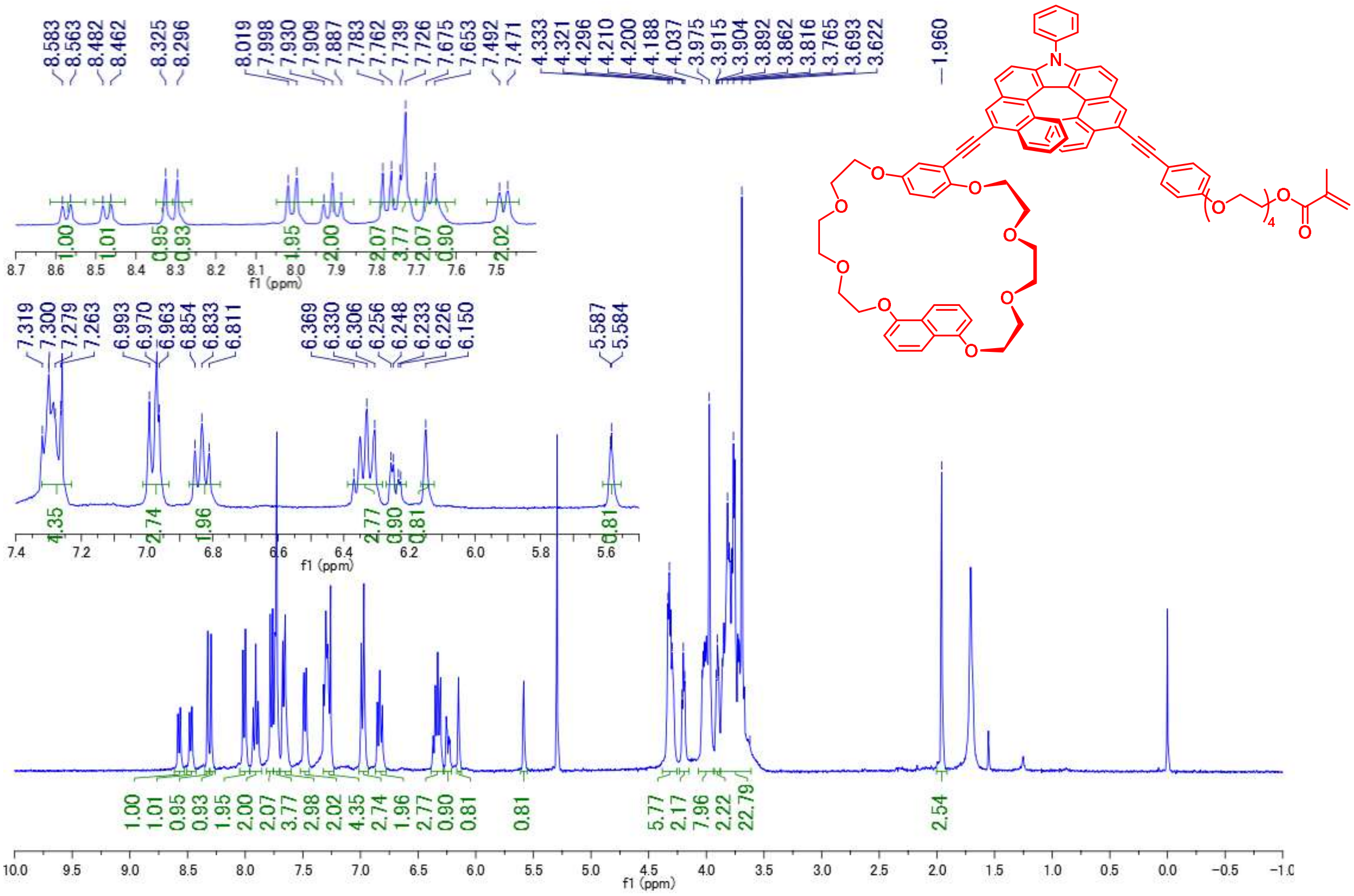

^{1}H NMR (400 MHz, $CDCl_3$) spectrum of compound **(*P*)-3**

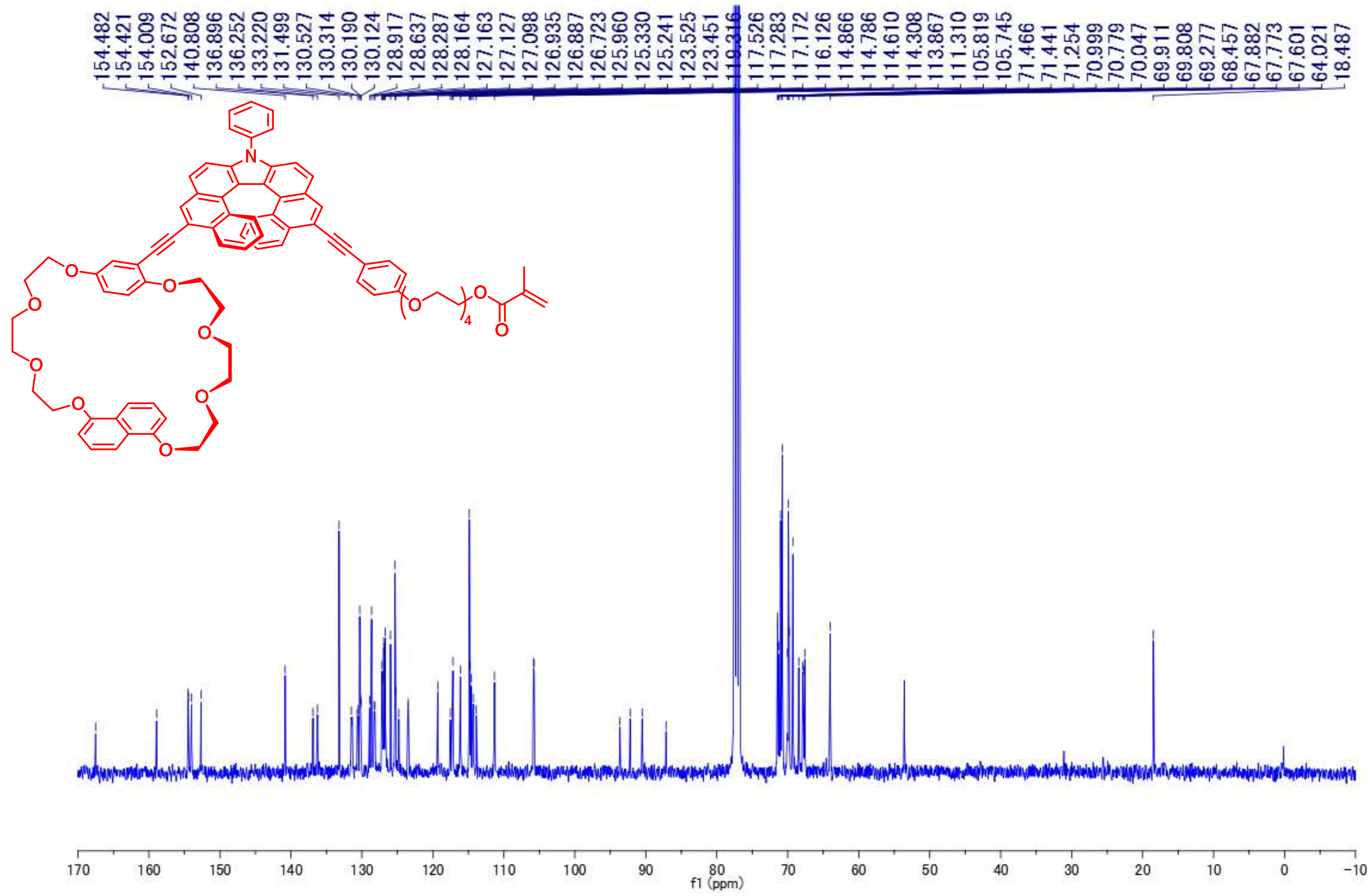

^{13}C NMR (100 MHz, $CDCl_3$) spectrum of compound **(*P*)-3**

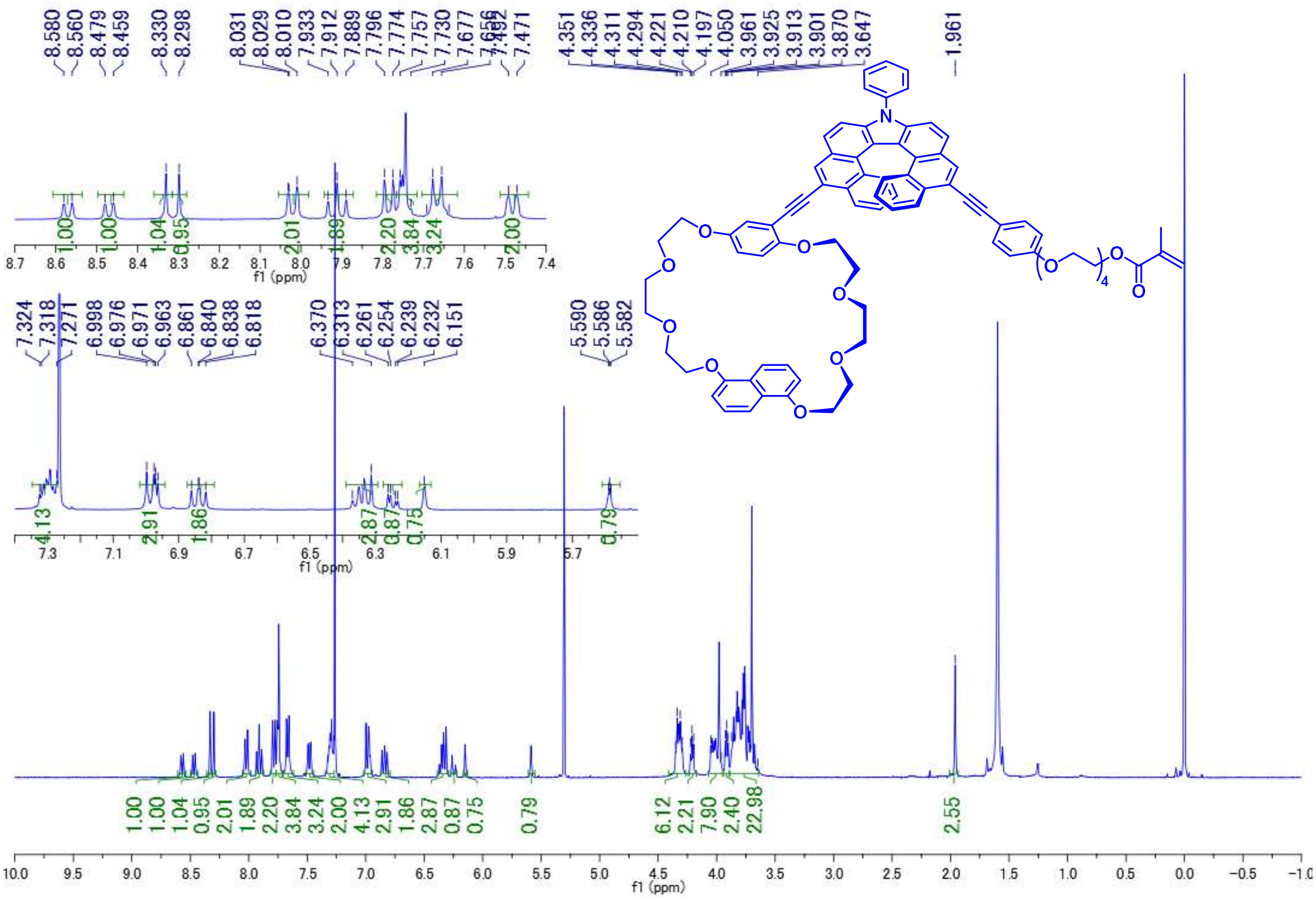

^{1}H NMR (400 MHz, $CDCl_3$) spectrum of compound **(*M*)-3**

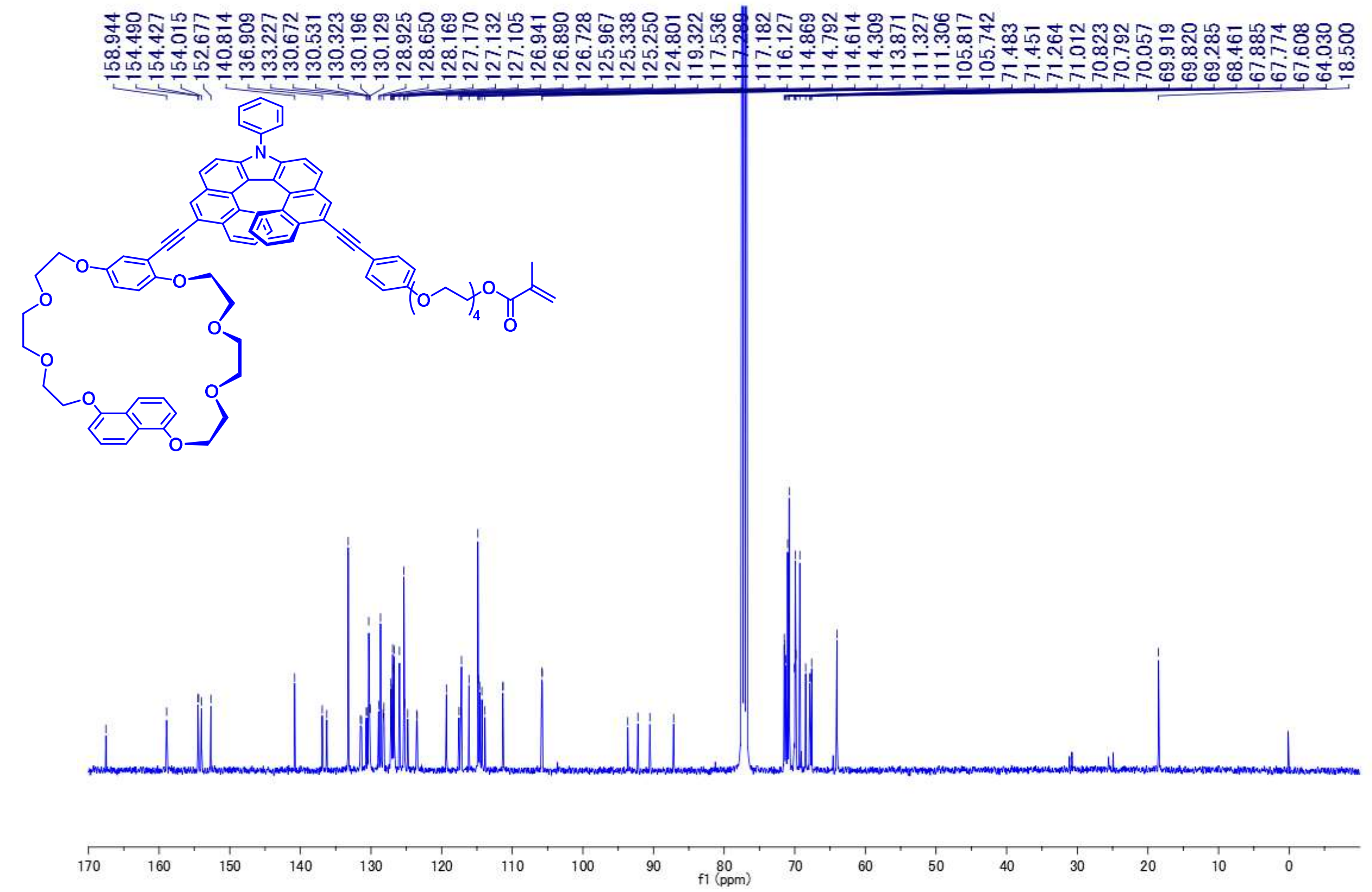

^{13}C NMR (100 MHz, $CDCl_3$) spectrum of compound **(*M*)-3**

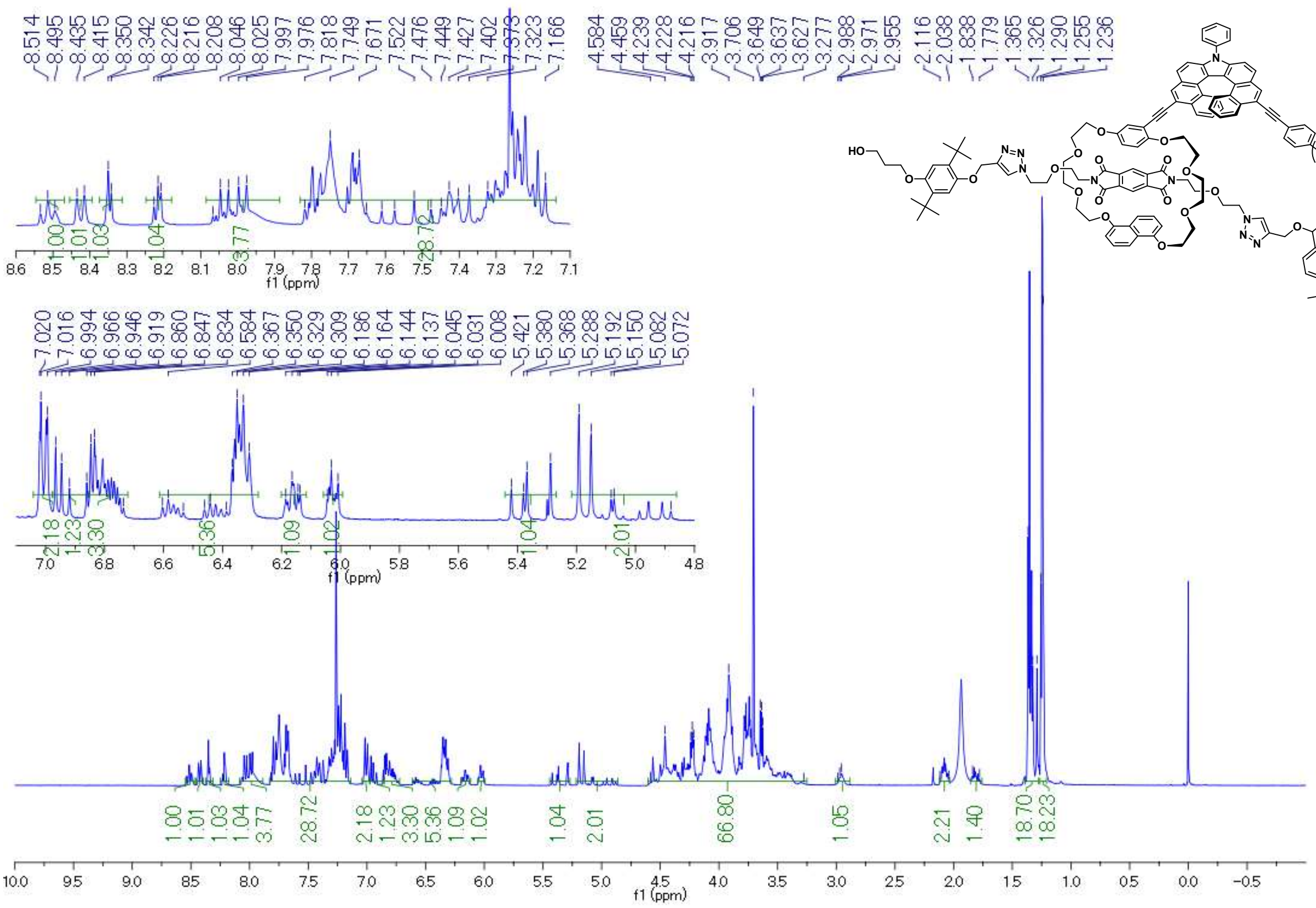

^{1}H NMR (400 MHz, $CDCl_3$) spectrum of compound ***rac*-2**

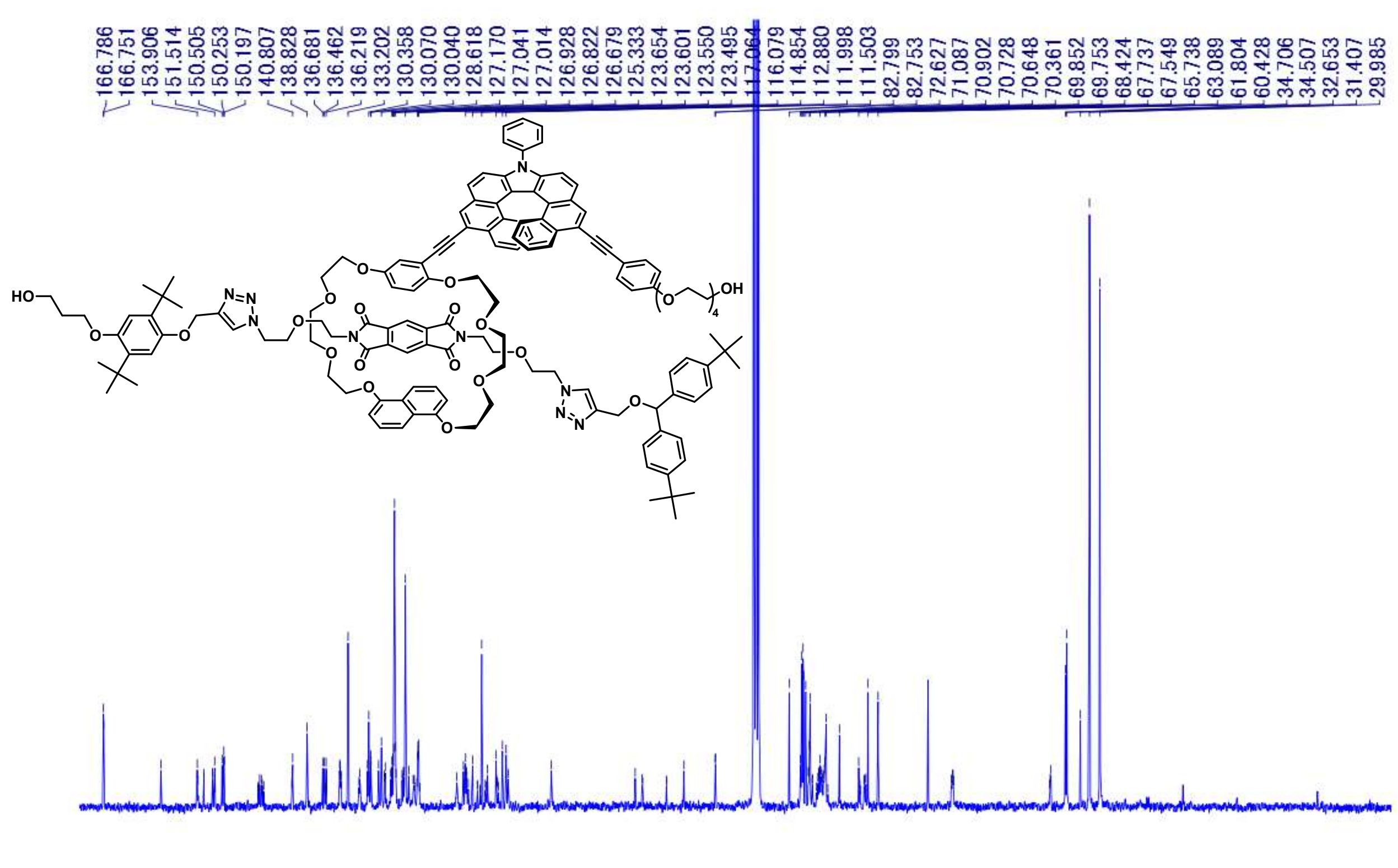

^{13}C NMR (100 MHz, $CDCl_3$) spectrum of compound ***rac*-2**

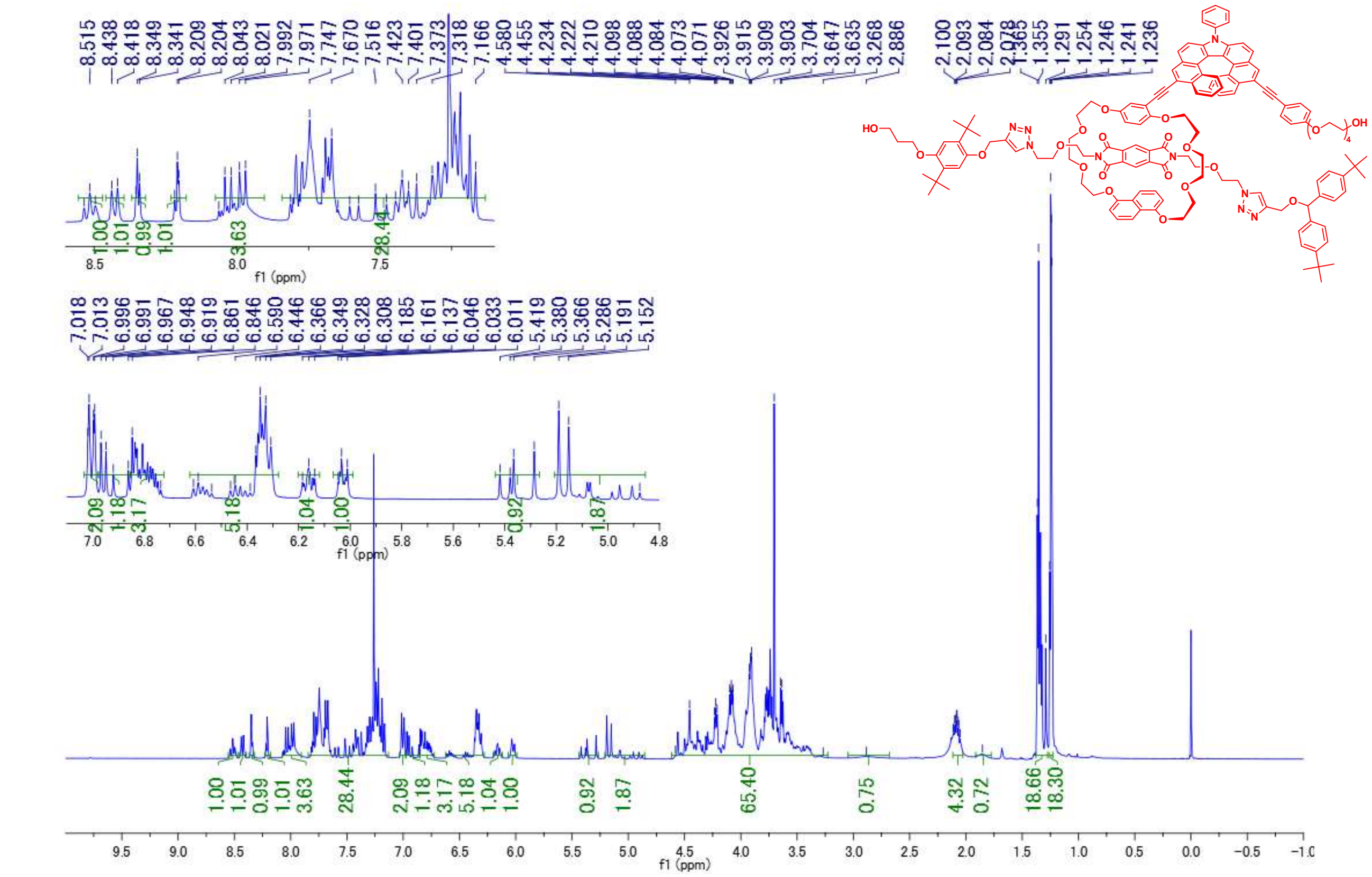

^{1}H NMR (400 MHz, $CDCl_3$) spectrum of compound **(*P*)-2**

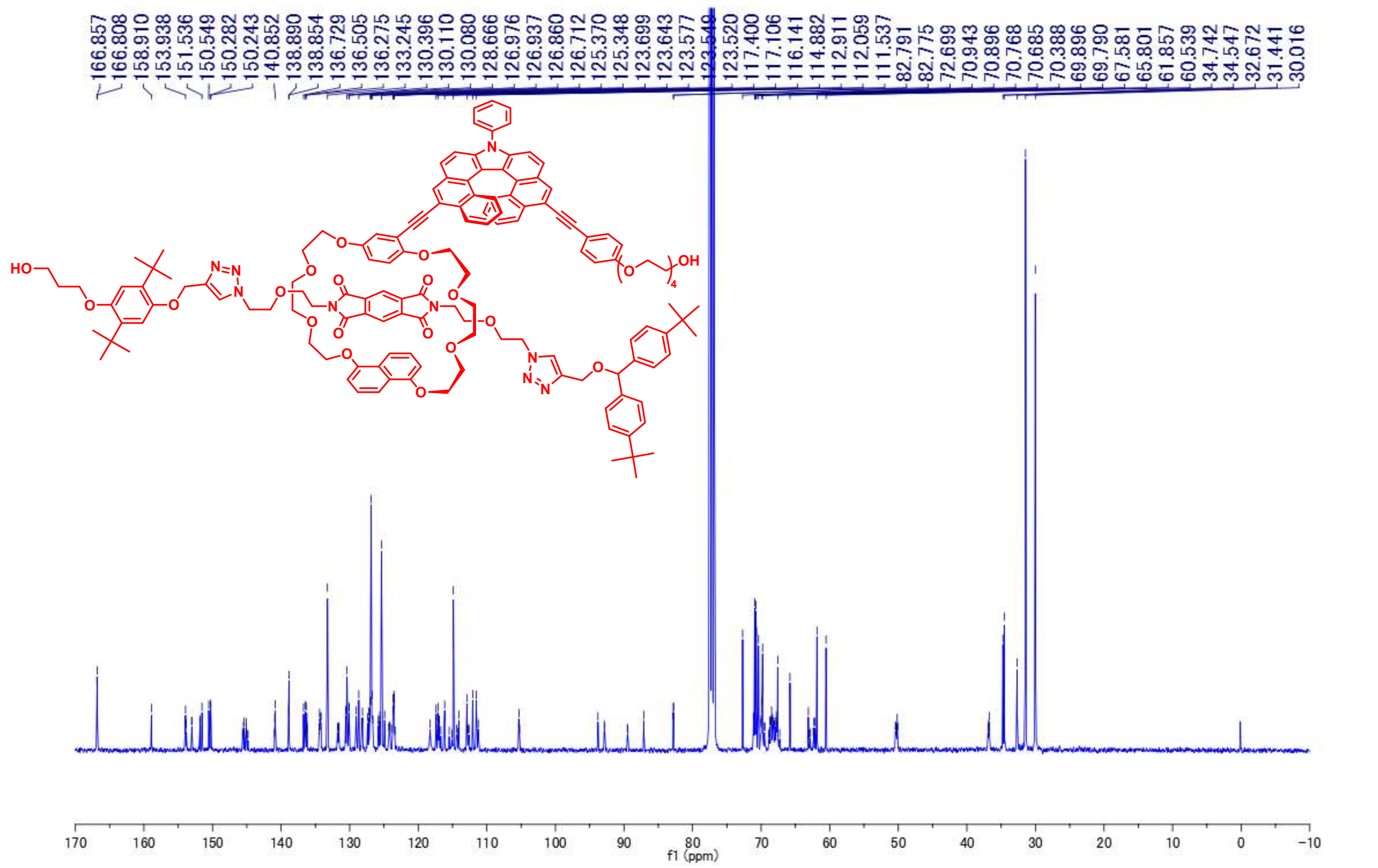

^{13}C NMR (100 MHz, $CDCl_3$) spectrum of compound **(*P*)-2**

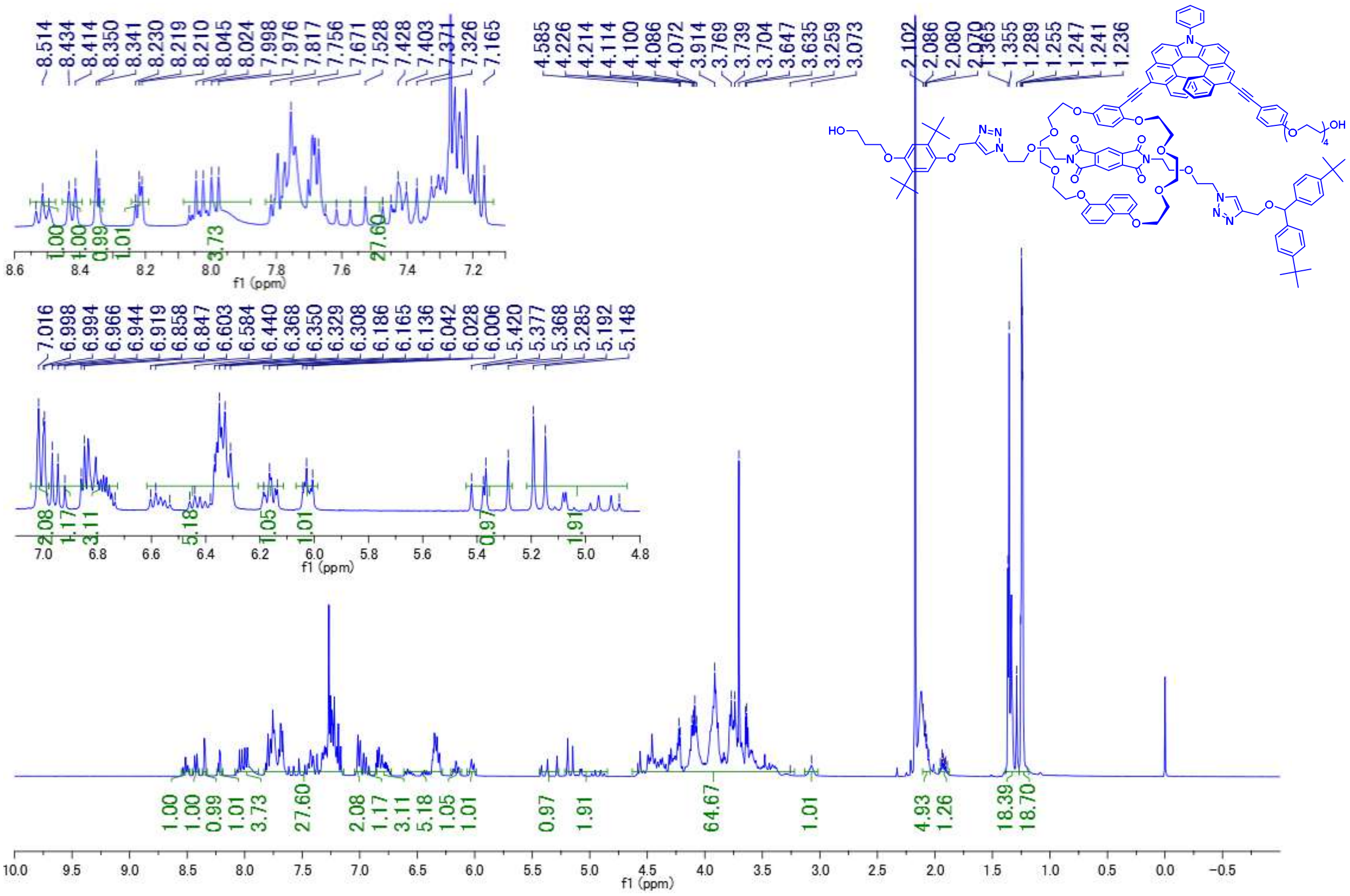

^{1}H NMR (400 MHz, $CDCl_3$) spectrum of compound **(*M*)-2**

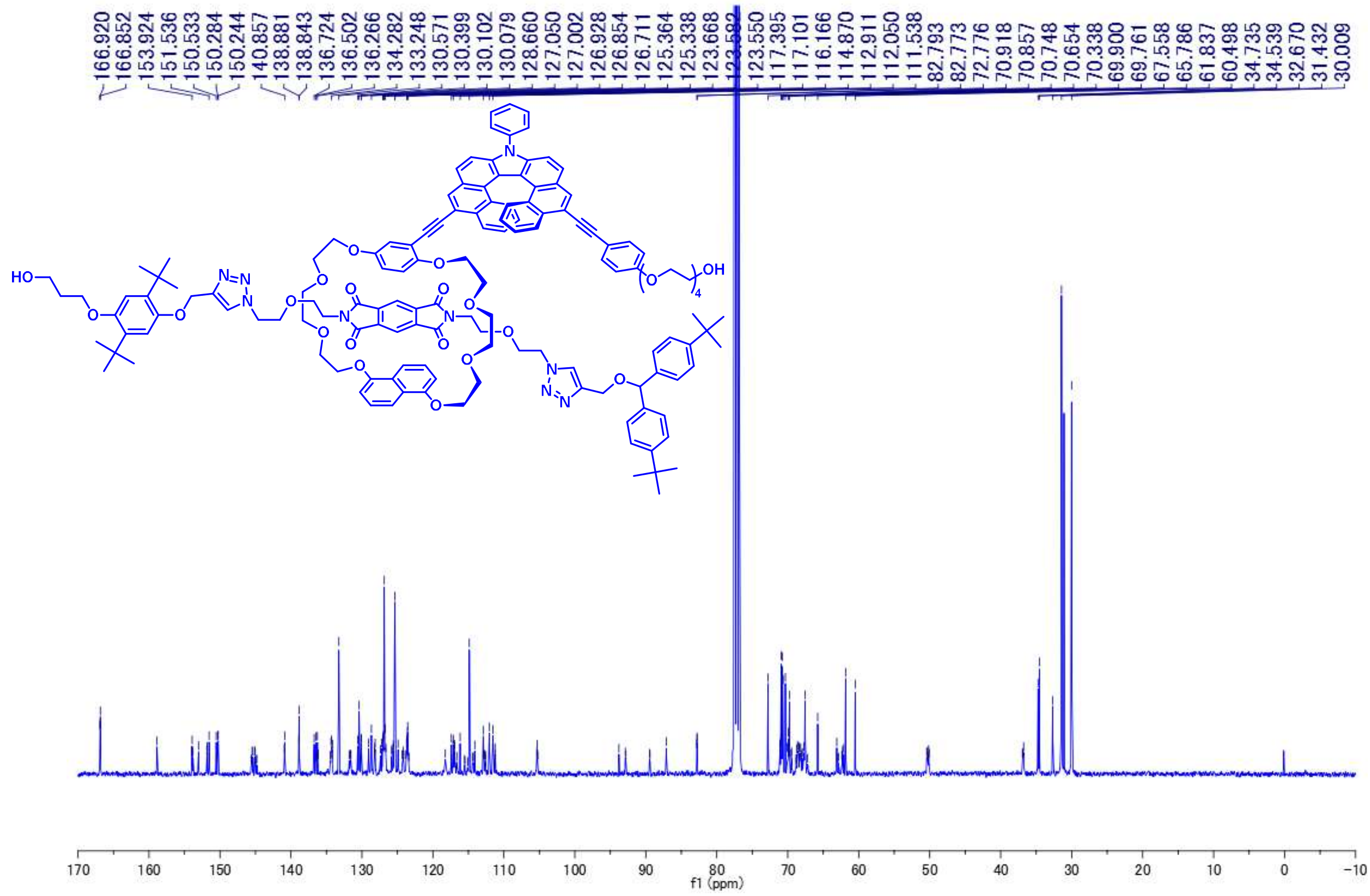

^{13}C NMR (100 MHz, $CDCl_3$) spectrum of compound **(*M*)-2**

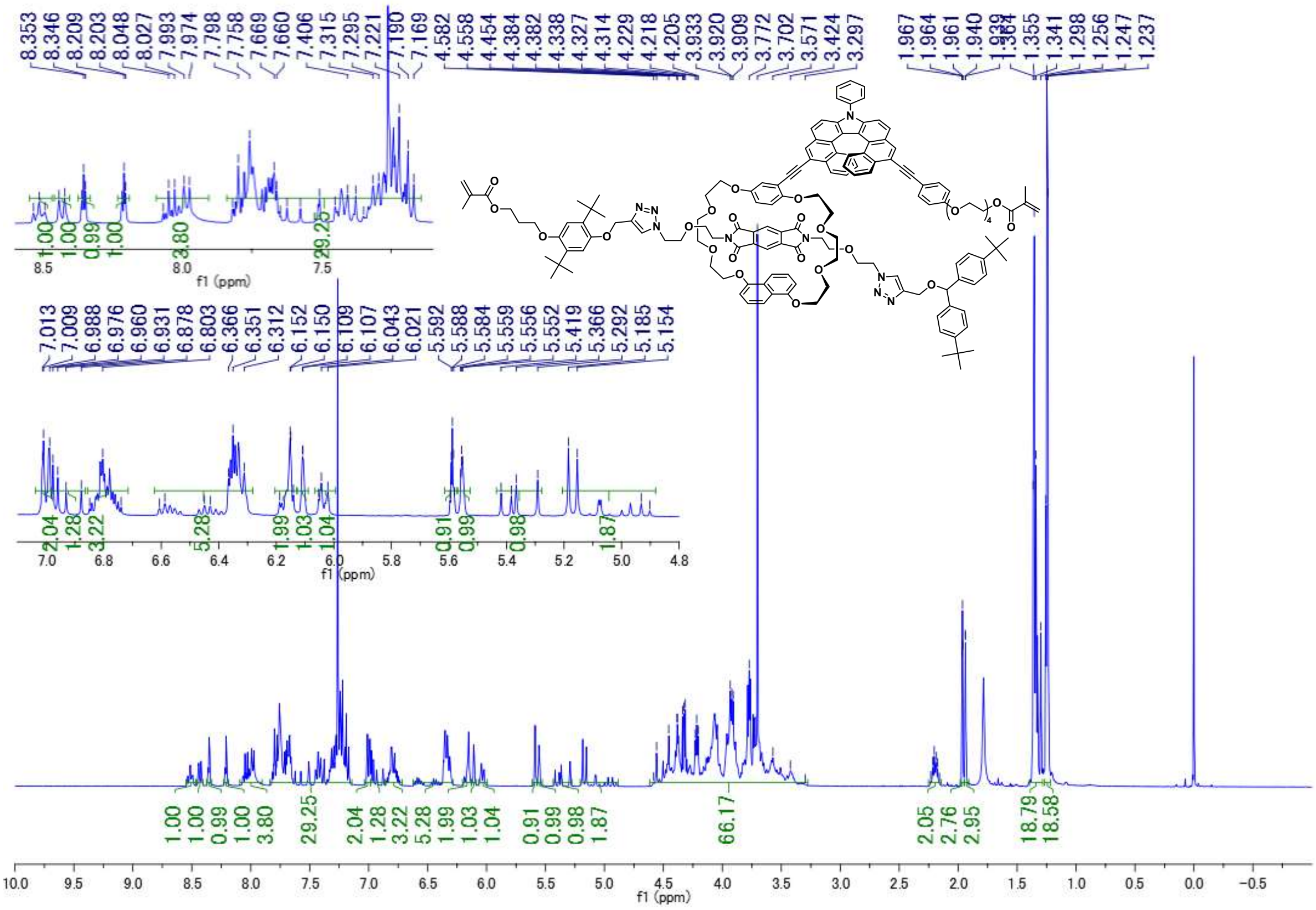

^{1}H NMR (400 MHz, $CDCl_3$) spectrum of compound ***rac*-1**

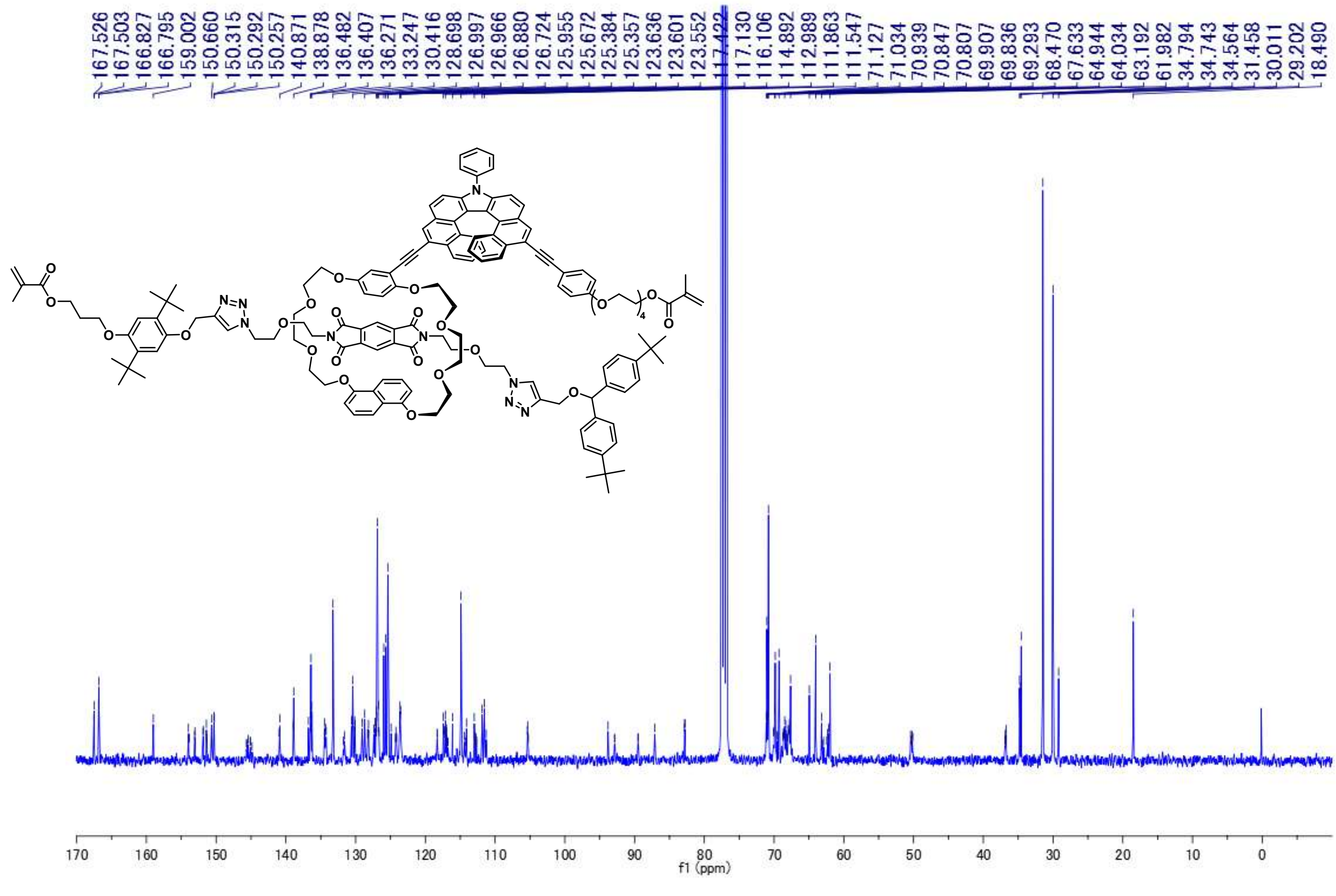

^{13}C NMR (100 MHz, $CDCl_3$) spectrum of compound ***rac*-1**

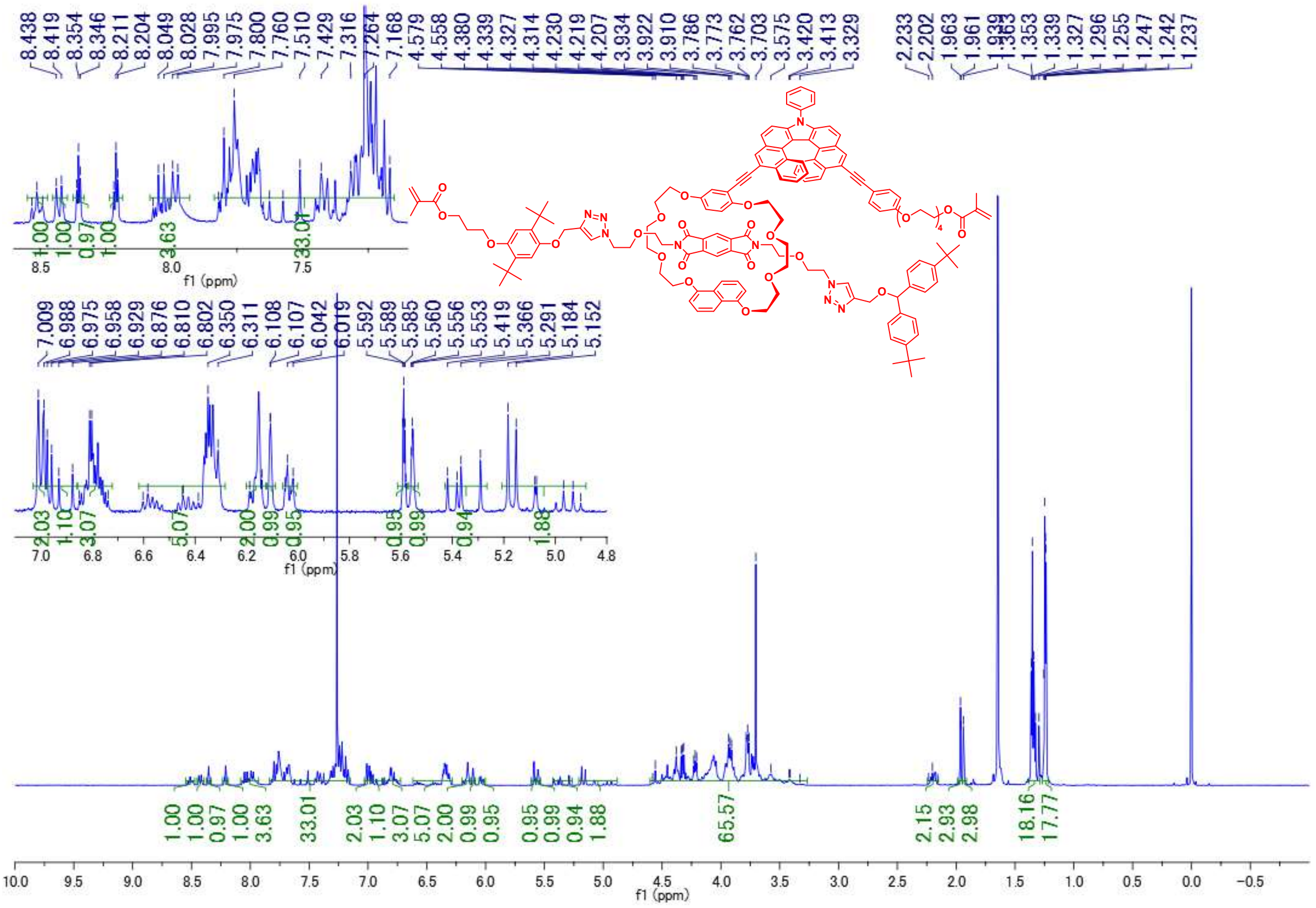

^{1}H NMR (400 MHz, $CDCl_3$) spectrum of compound **(*P*)-1**

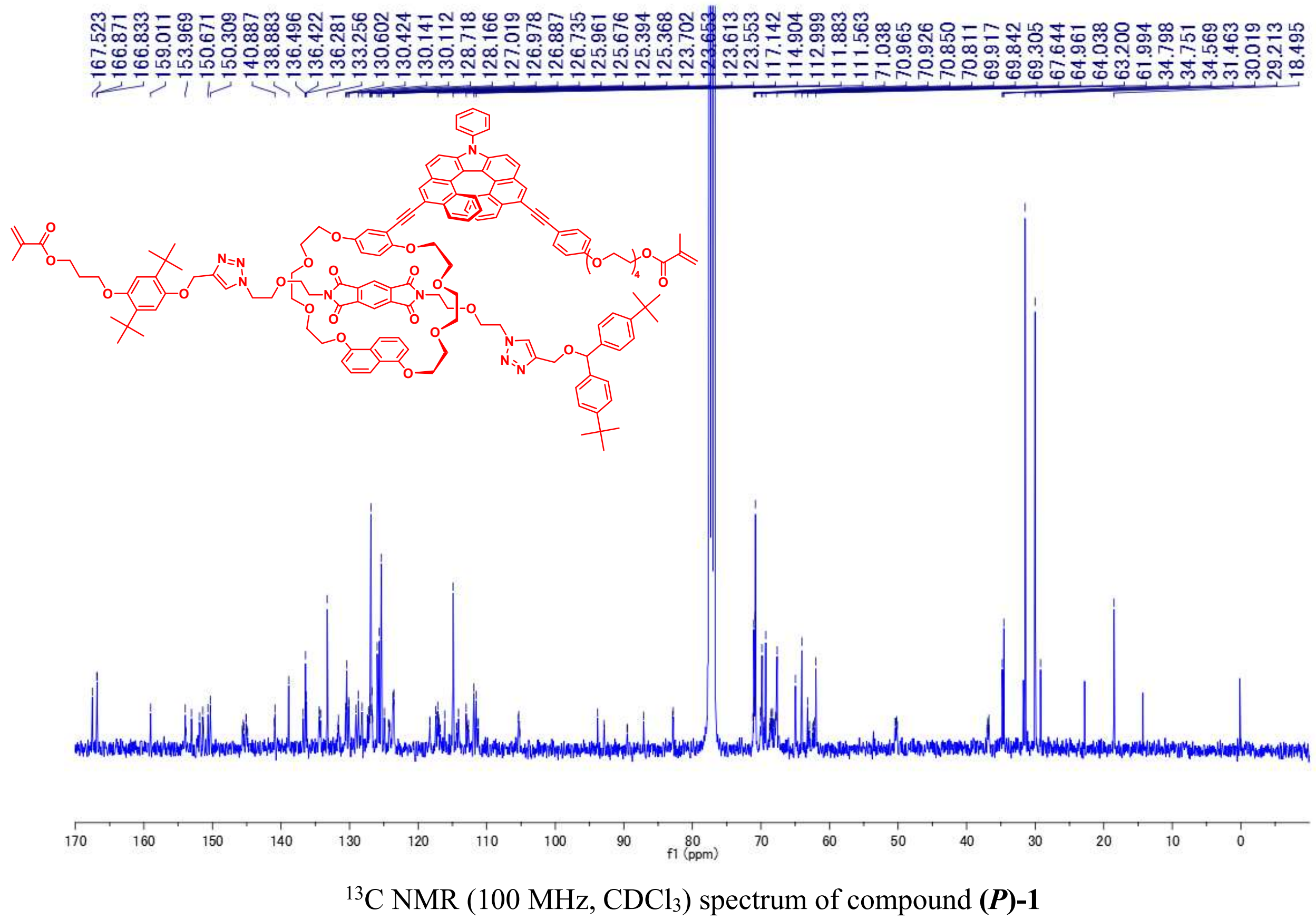

^{13}C NMR (100 MHz, $CDCl_3$) spectrum of compound **(*P*)-1**

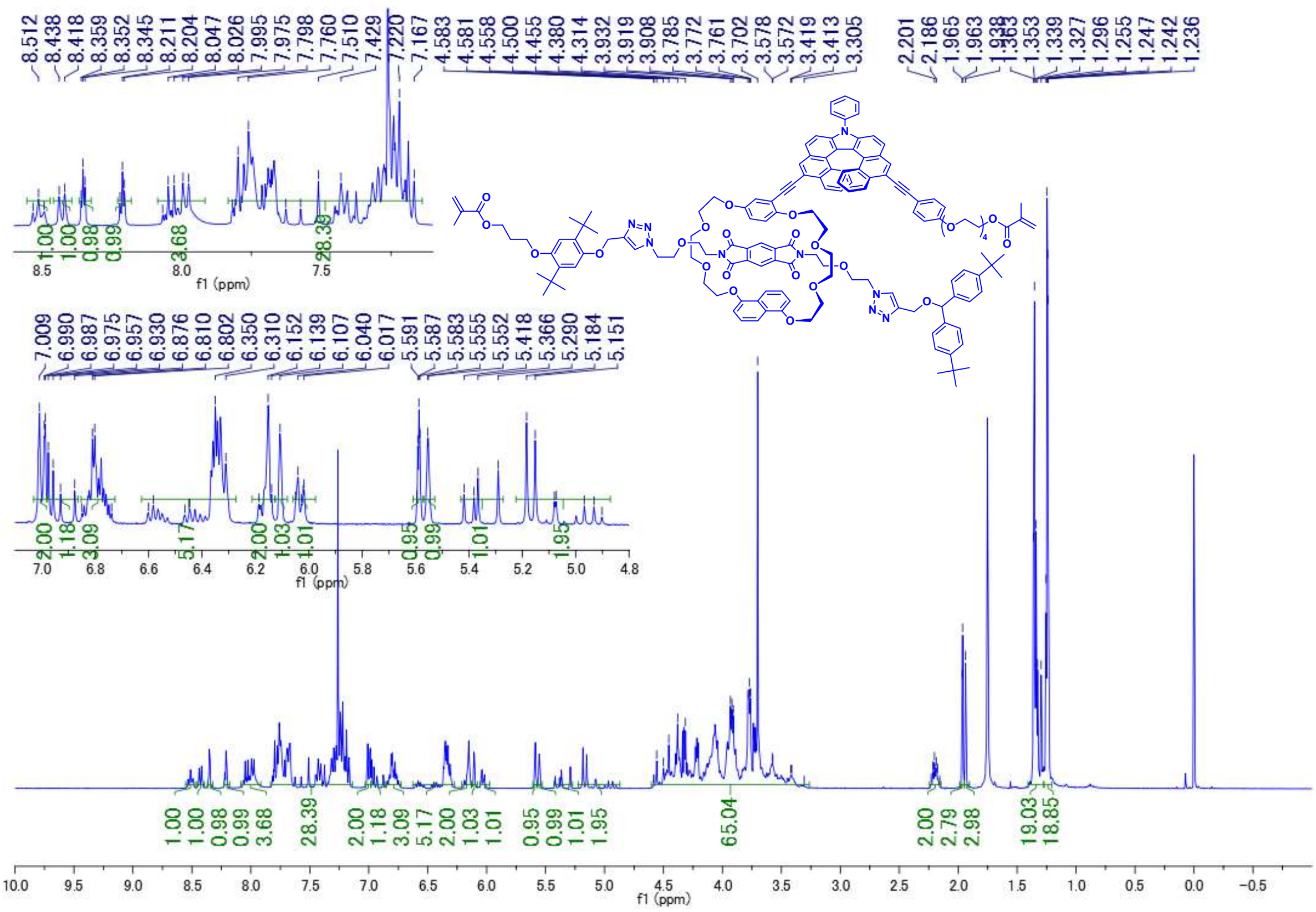

^{1}H NMR (400 MHz, $CDCl_3$) spectrum of compound ***(M)*-1**

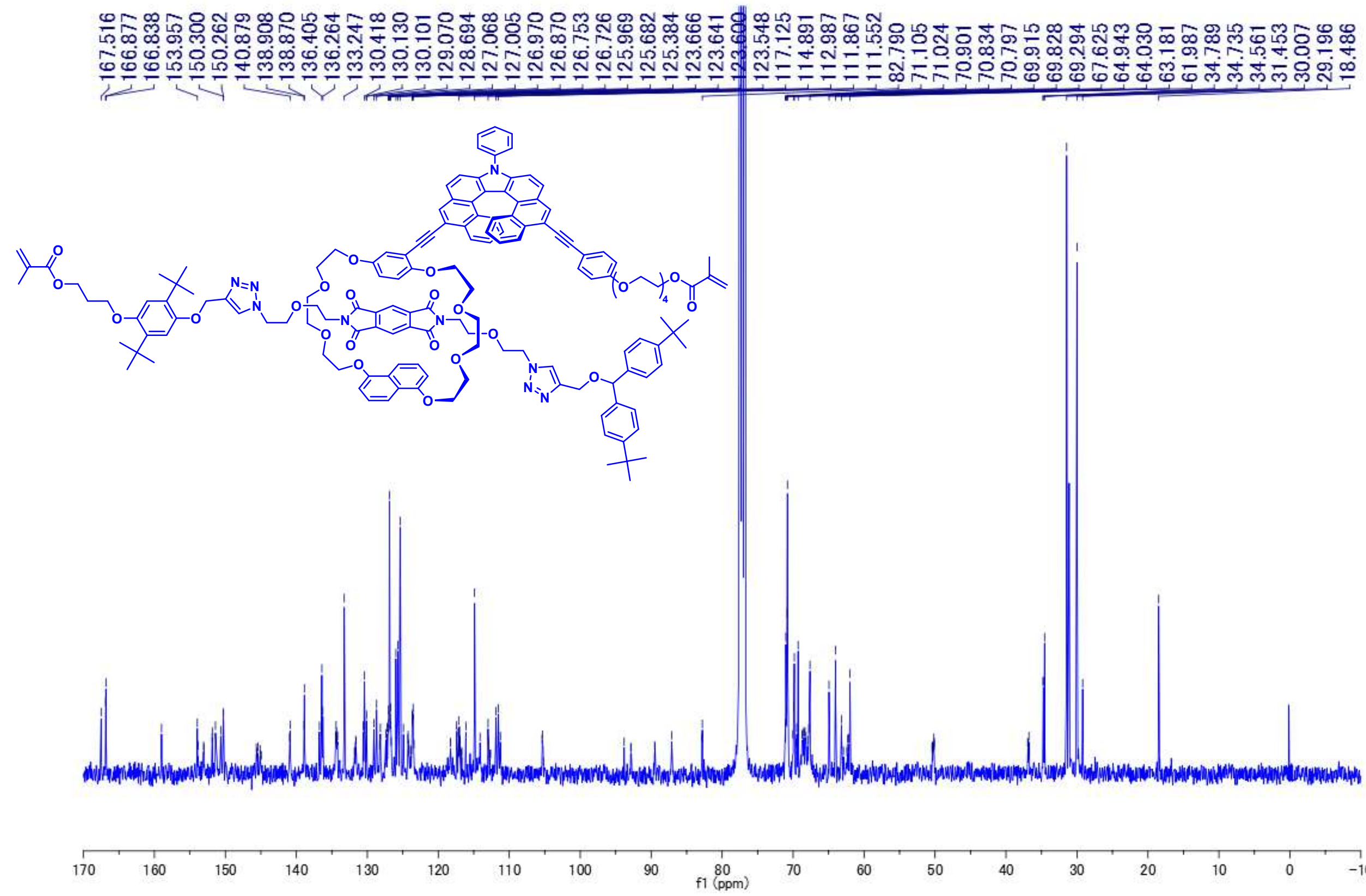

^{13}C NMR (100 MHz, $CDCl_3$) spectrum of compound ***(M)*-1**

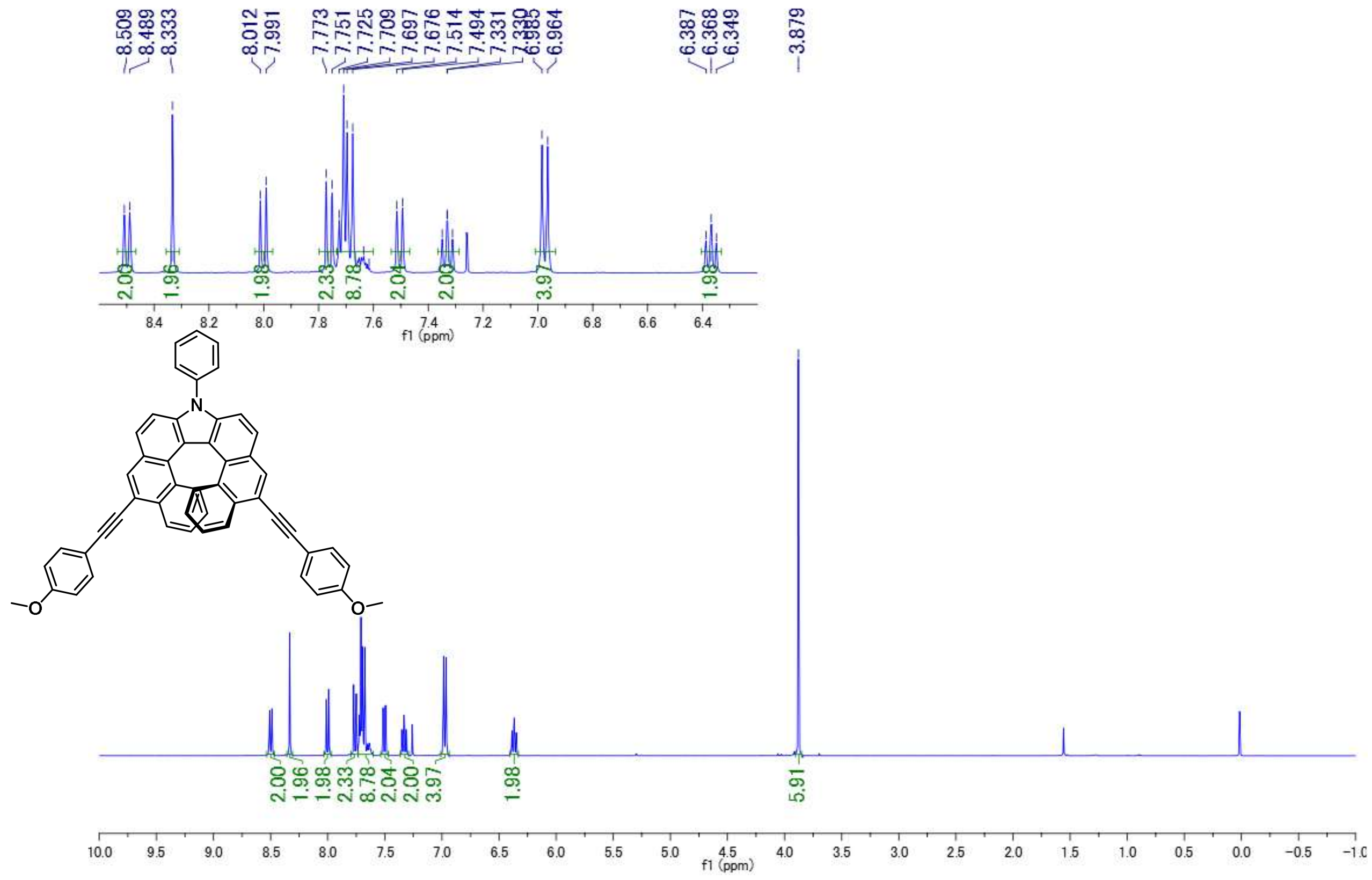

^{1}H NMR (400 MHz, $CDCl_3$) spectrum of compound ***rac*-Ref-Hel**

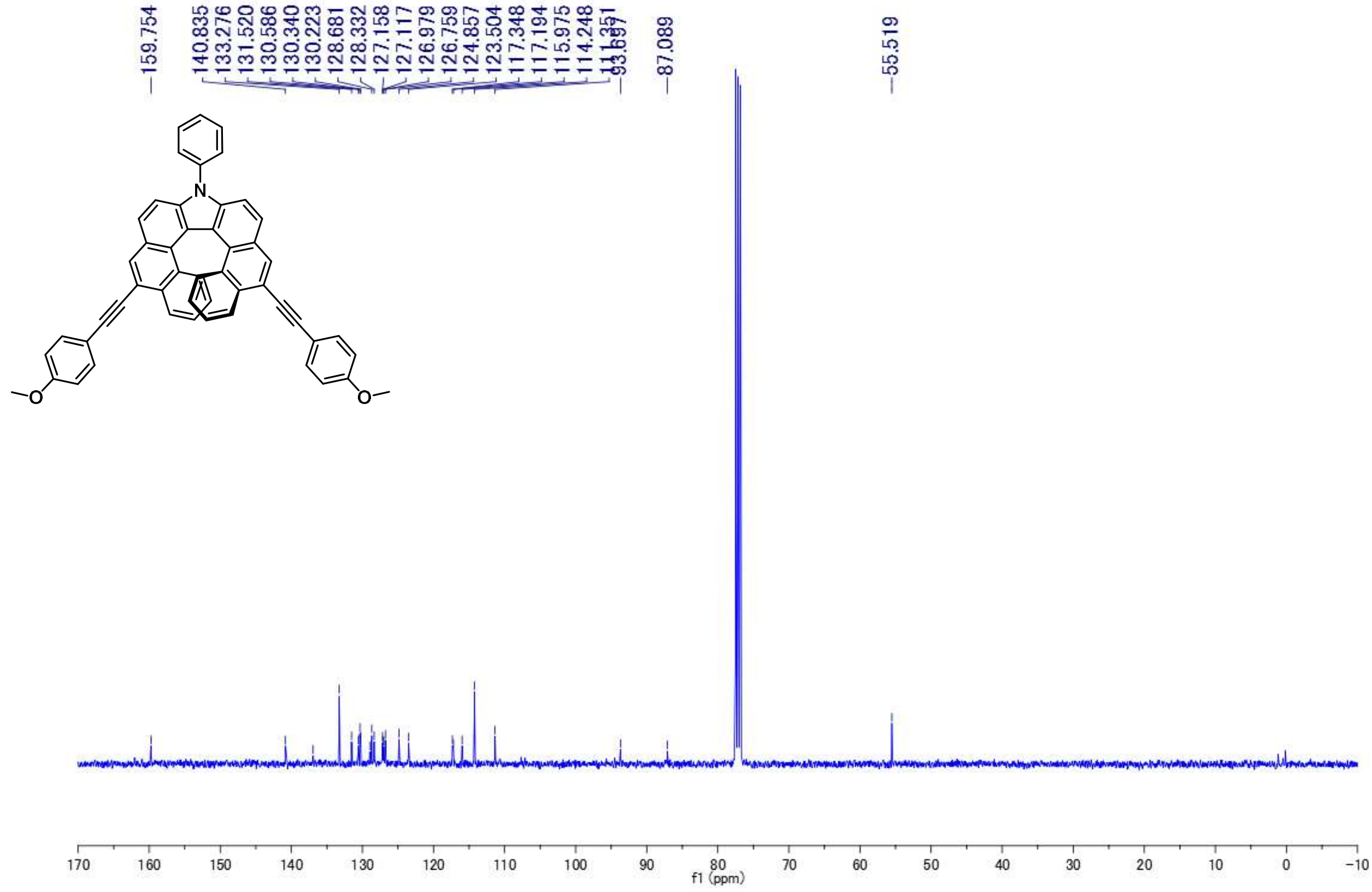

^{13}C NMR (100 MHz, $CDCl_3$) spectrum of compound ***rac*-Ref-Hel**

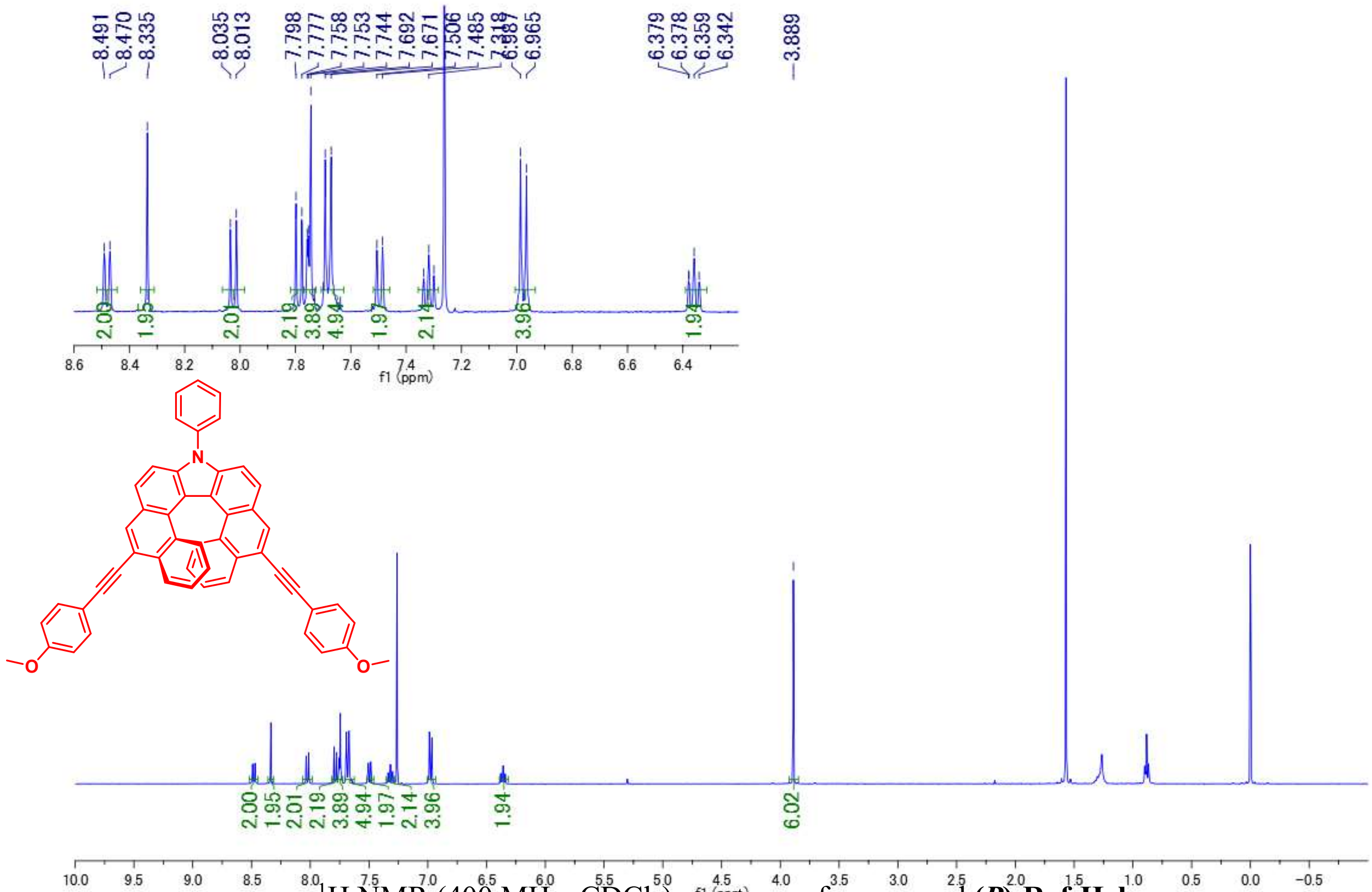

^{1}H NMR (400 MHz, $CDCl_3$) spectrum of compound **(*P*)-Ref-Hel**

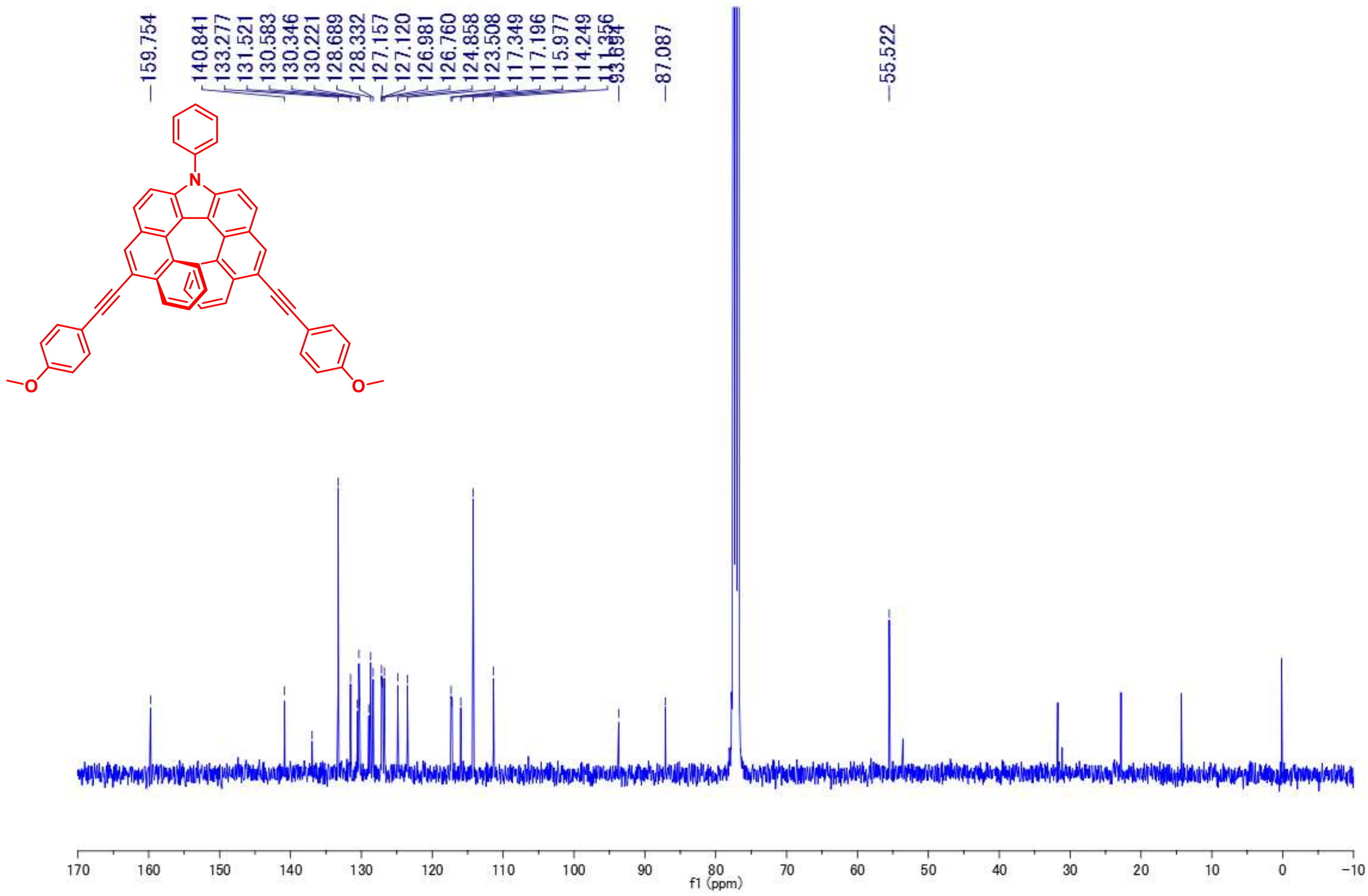

^{13}C NMR (100 MHz, $CDCl_3$) spectrum of compound **(*P*)-Ref-Hel**

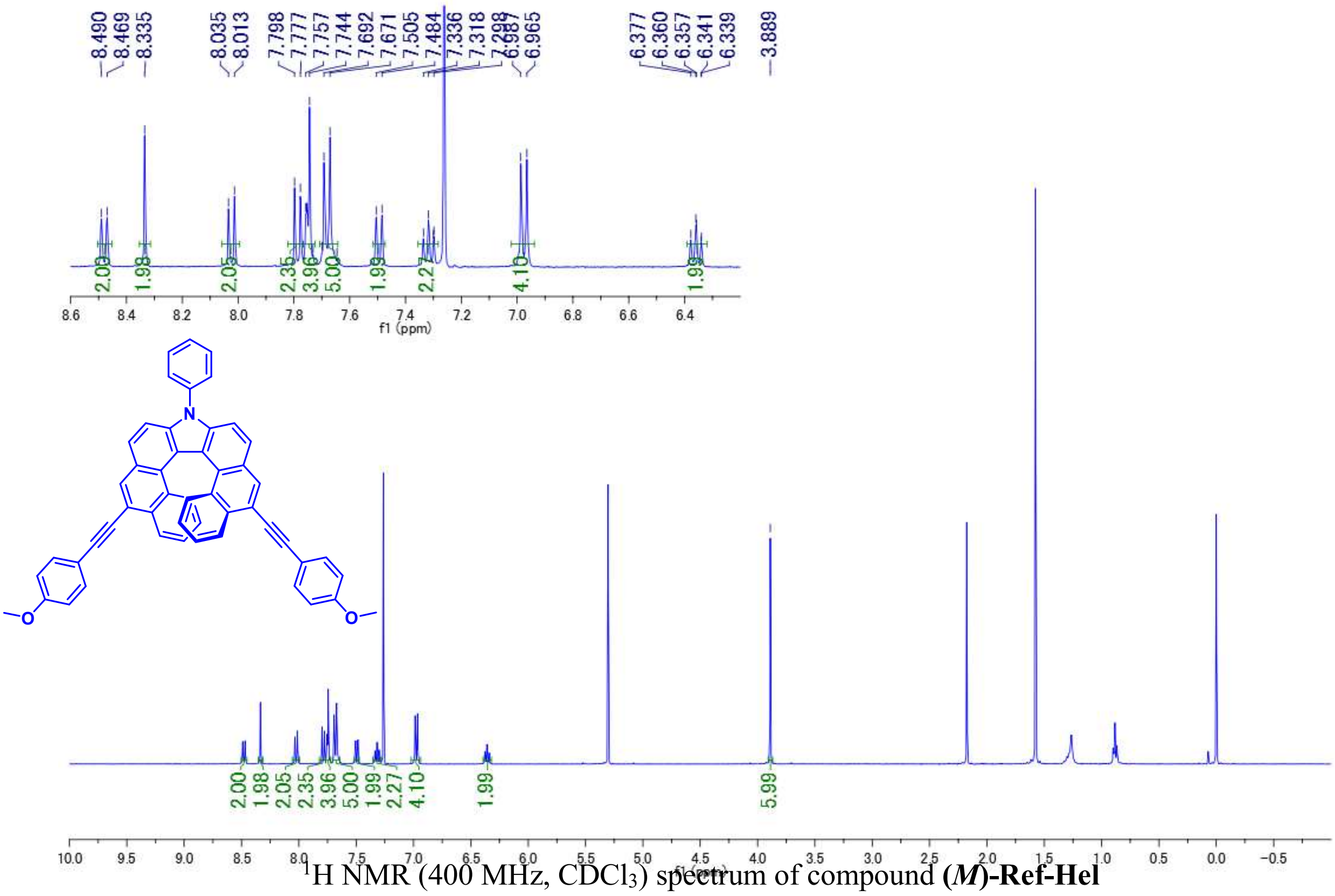

^{1}H NMR (400 MHz, $CDCl_3$) spectrum of compound ***(M)*-Ref-Hel**

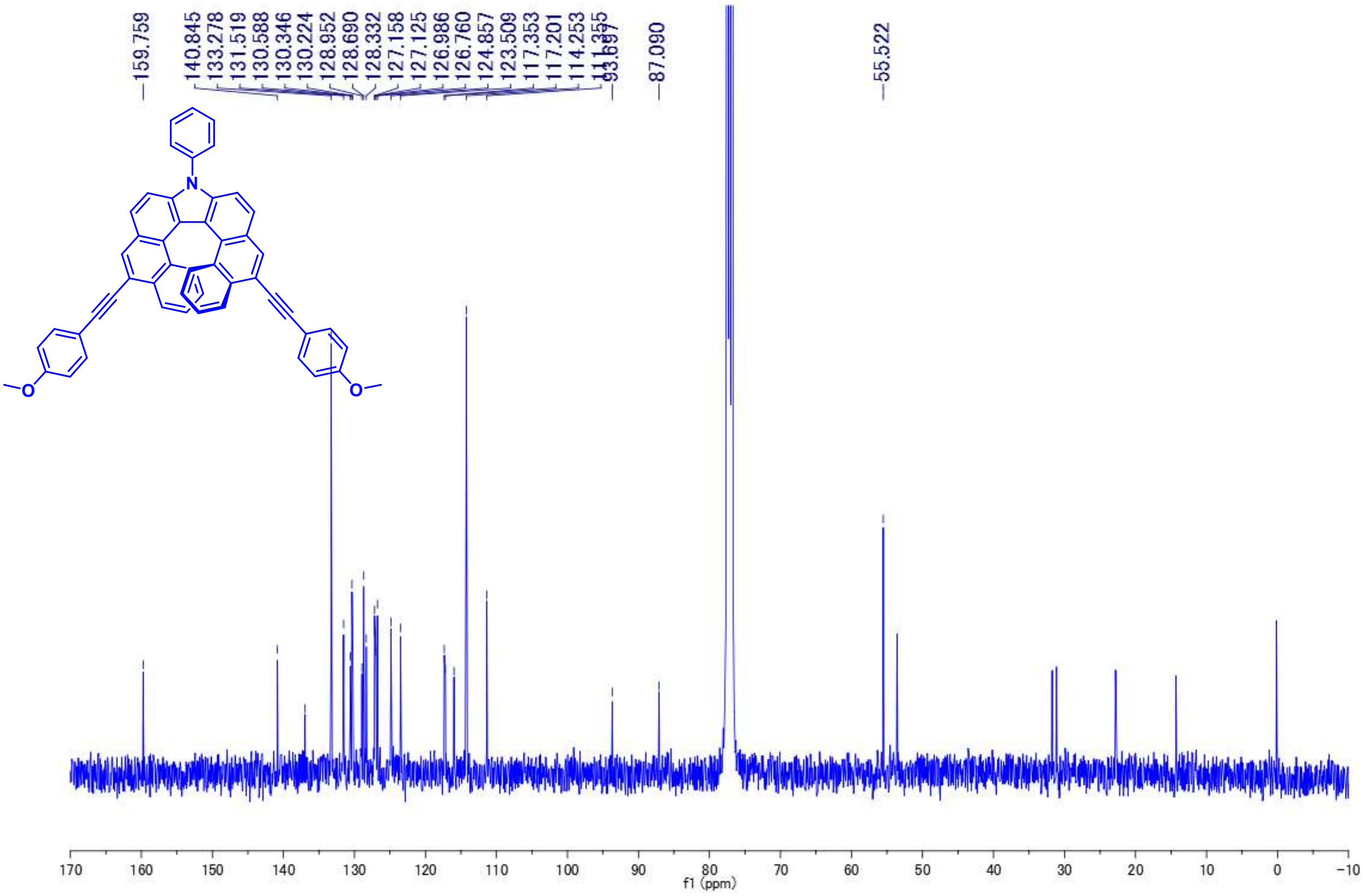

^{13}C NMR (100 MHz, $CDCl_3$) spectrum of compound ***(M)*-Ref-Hel**

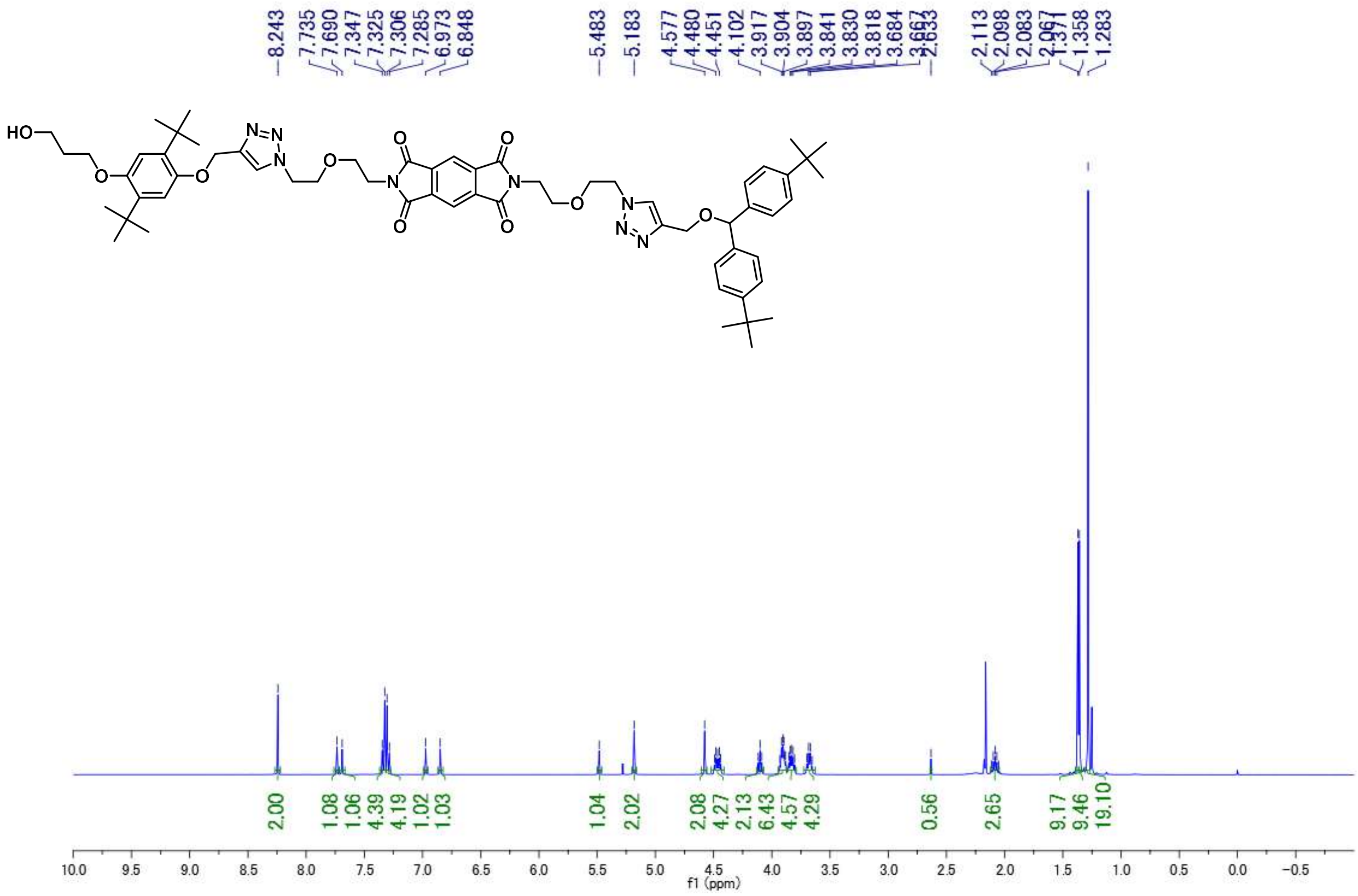

^{1}H NMR (400 MHz, $CDCl_3$) spectrum of compound **13**

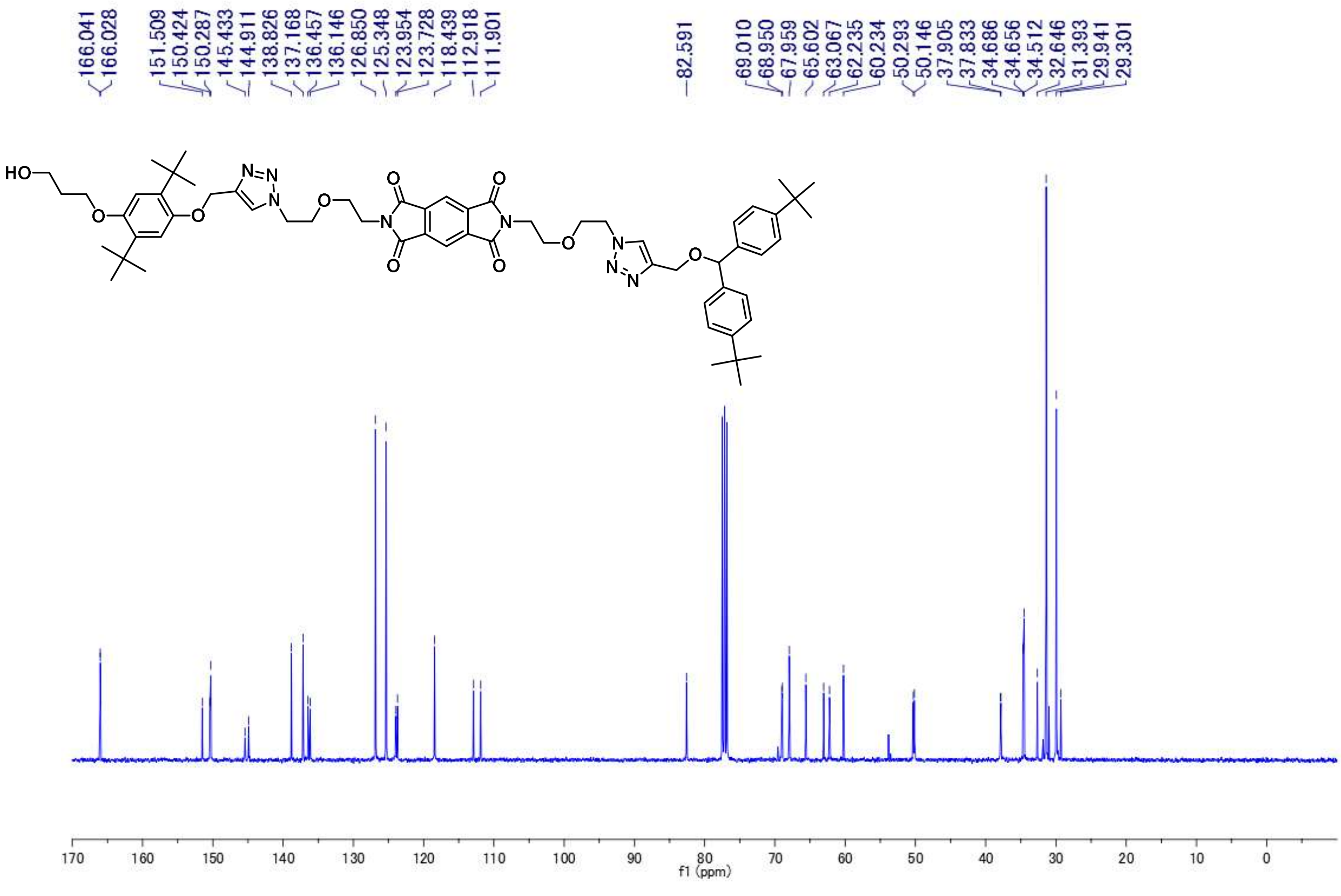

^{13}C NMR (100 MHz, $CDCl_3$) spectrum of compound **13**

Mass Spectra of Rotaxanes

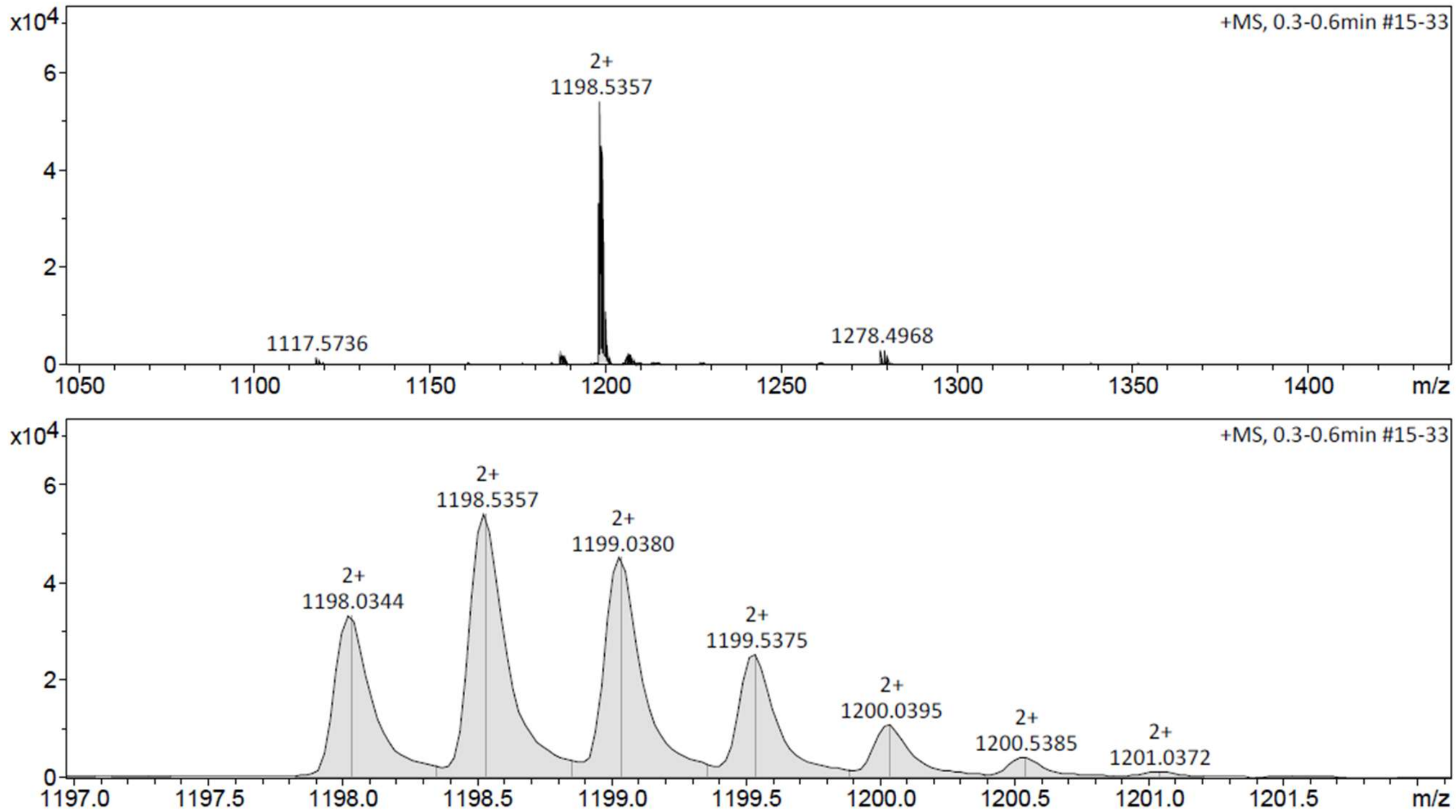

High resolution ESI-TOF-MS spectrum of ***rac*-2**

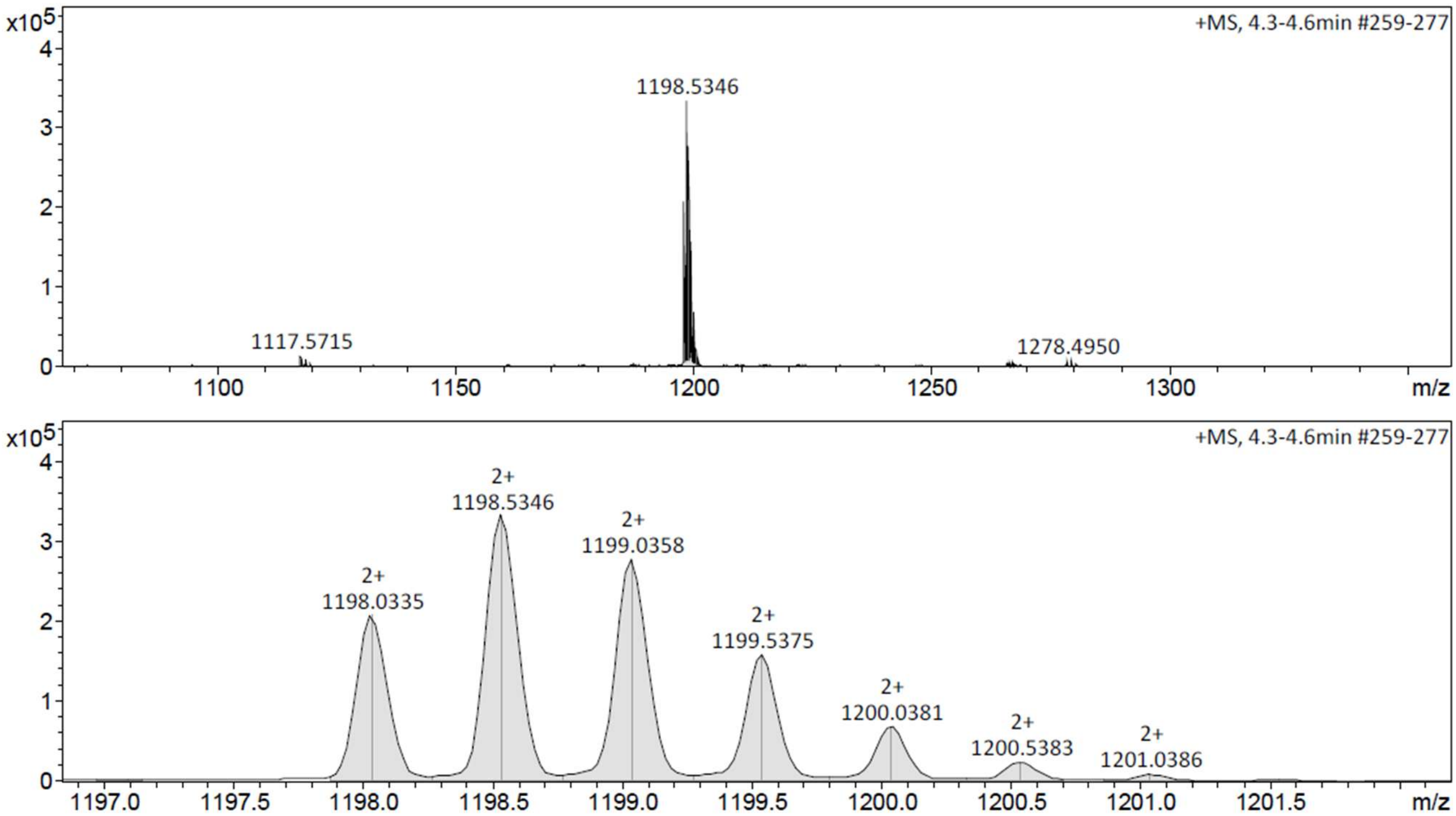

High resolution ESI-TOF-MS spectrum of **(*P*)-2**

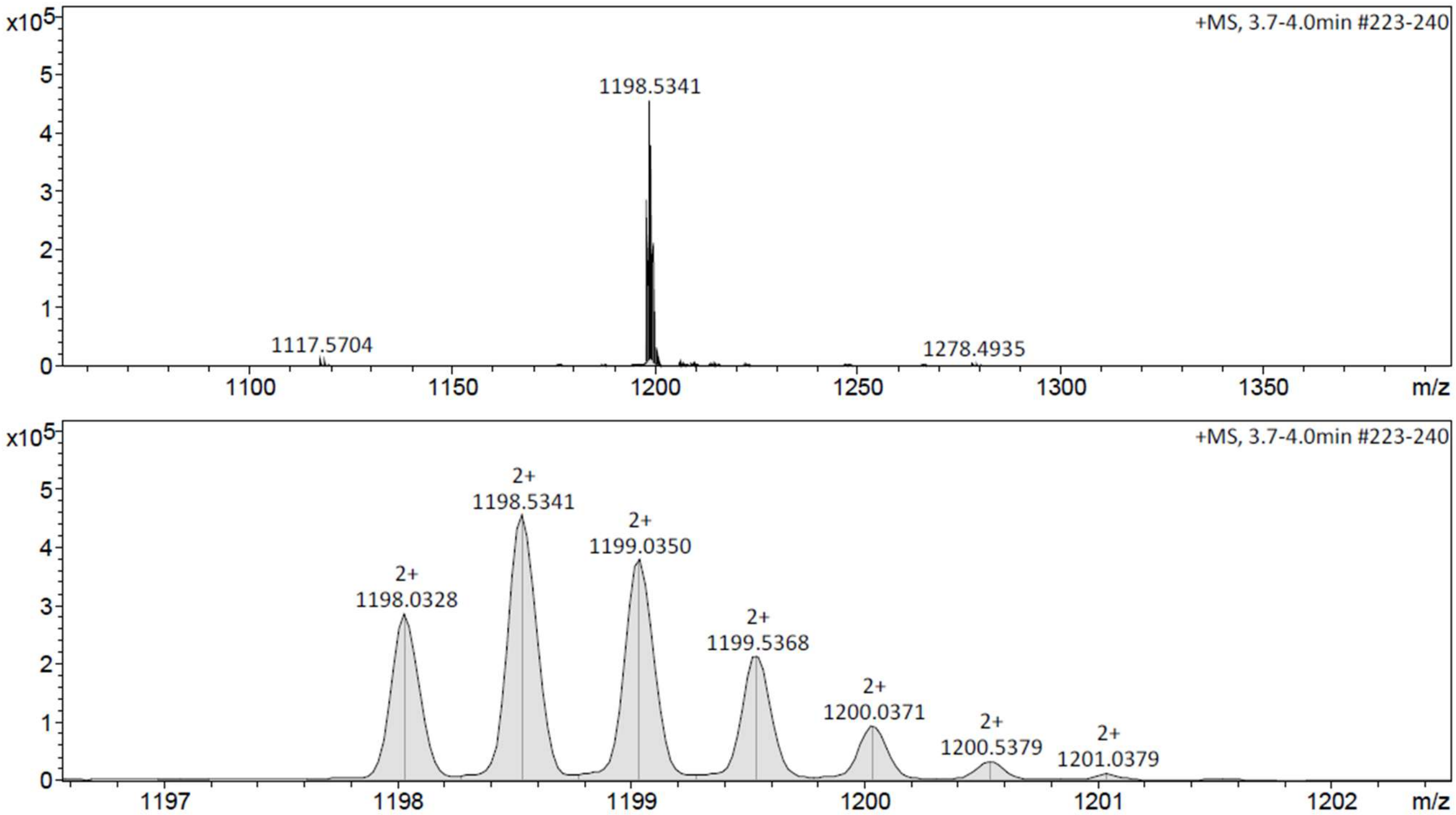

High resolution ESI-TOF-MS spectrum of **(*M*)-2**

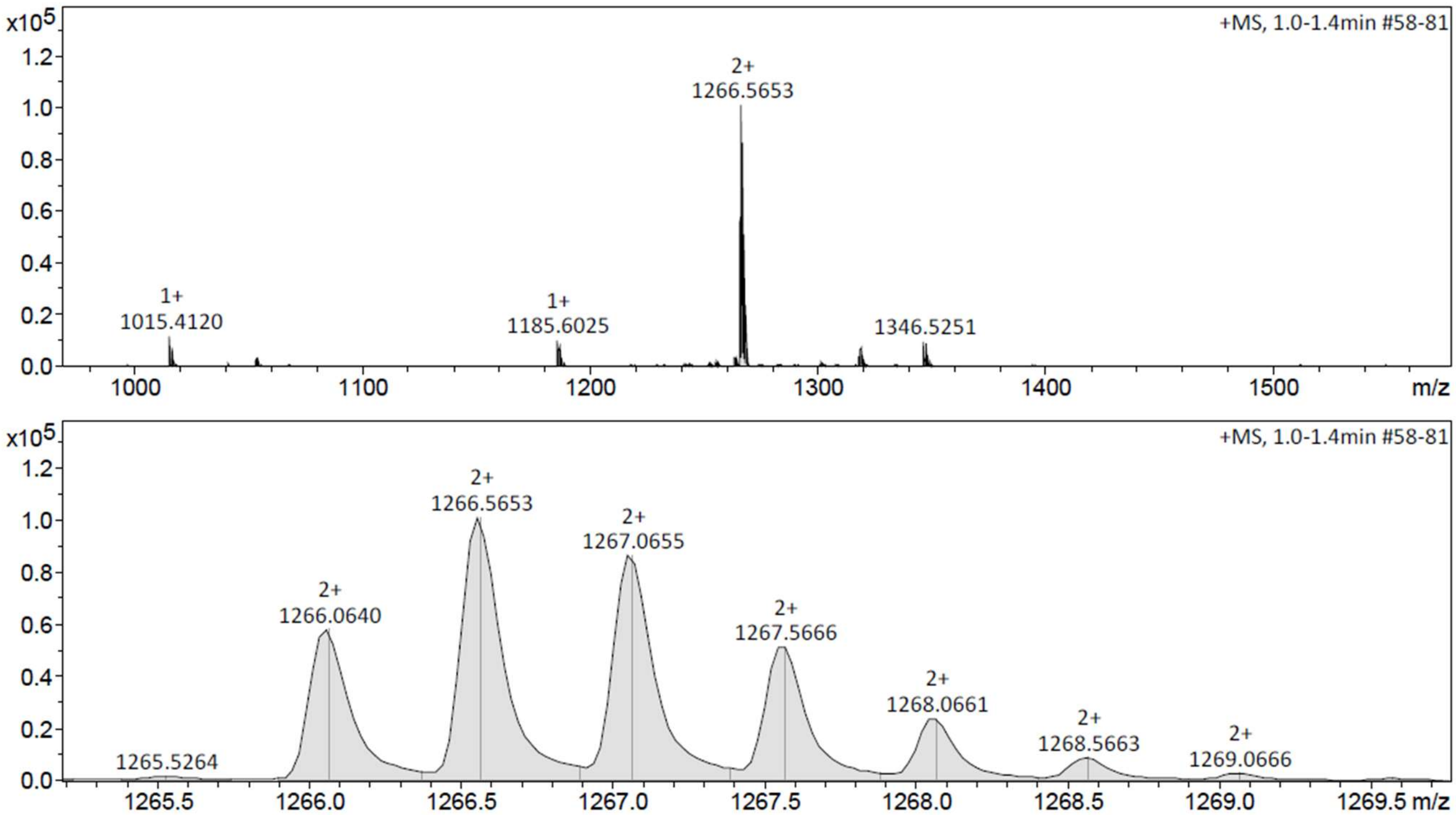

High resolution ESI-TOF-MSspectrum of ***rac*-1**

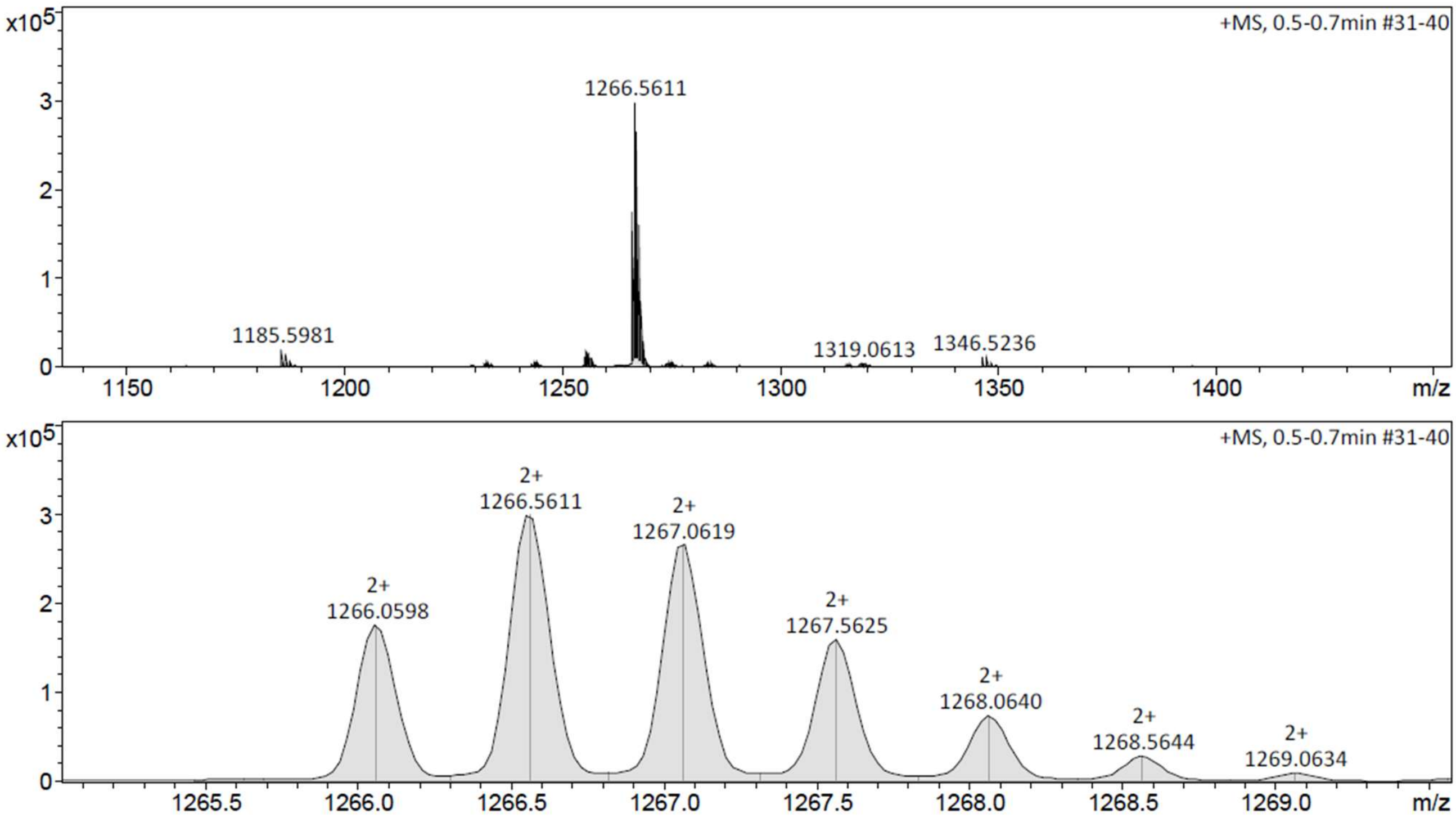

High resolution ESI-TOF-MS spectrum of **(*P*)-1**

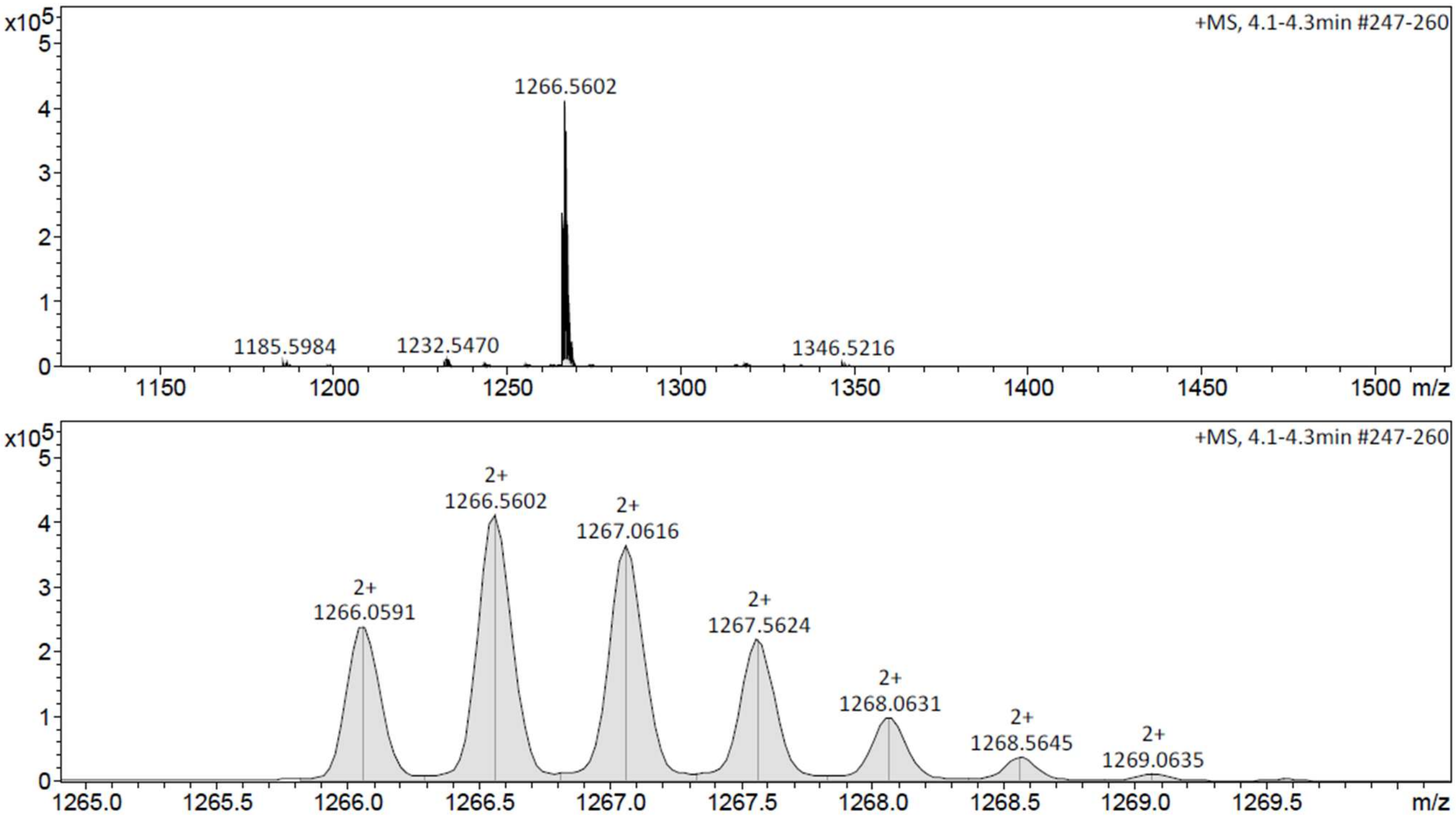

High resolution ESI-TOF-MSspectrum of **(*M*)-1**

DFT-optimized Coordinates

1,5-Dimethoxynaphthalene (Figure S1, left)

C	-2.08231600	-1.42685100	0.00005900
C	-1.86692000	-0.07295000	0.00012200
C	-0.54076800	0.45412700	0.00006200
C	0.54076800	-0.45412700	-0.00006200
C	0.30000900	-1.84936500	-0.00012500
C	-0.98274600	-2.31221200	-0.00006500
H	-1.13958100	2.52838400	0.00021900
H	-3.08375100	-1.83095200	0.00010300
C	-0.30000900	1.84936500	0.00012400
C	1.86692000	0.07295000	-0.00012200
H	1.13958100	-2.52838400	-0.00021900
H	-1.17371000	-3.37755100	-0.00011300
C	2.08231600	1.42685100	-0.00005900
C	0.98274600	2.31221200	0.00006400
H	3.08375100	1.83095200	-0.00010400
H	1.17371000	3.37755100	0.00011200
O	-2.85162900	0.85839600	0.00024200
O	2.85162900	-0.85839600	-0.00024200
C	-4.18426000	0.40278700	0.00030500
H	-4.81012700	1.29109600	0.00039700
H	-4.39695200	-0.19331600	-0.89123700
H	-4.39683200	-0.19341600	0.89180800
C	4.18426000	-0.40278700	-0.00030300
H	4.81012700	-1.29109600	-0.00039300
H	4.39683400	0.19341600	-0.89180600
H	4.39695000	0.19331600	0.89123900

π-extended ***(M)*-A7Hel** (Figure S1, middle)

O	10.39128100	-5.10557300	-0.23360200
C	9.30314600	-4.30870200	-0.11417600
C	9.49533300	-2.96131800	-0.42520600
C	8.44791300	-2.07219000	-0.33406400
C	7.17579700	-2.50036500	0.07083000
C	6.09116000	-1.57822300	0.16559600
C	5.17823000	-0.79489200	0.24450900
C	4.09628600	0.12990900	0.33354600
C	2.79032900	-0.31223900	0.76312100
C	1.72719900	0.61784200	0.82483100
C	0.53568300	0.21938100	1.46216900
C	0.35163700	-1.06966700	1.89281500
C	1.36637600	-2.01946300	1.71283300
C	2.57204600	-1.63827800	1.17980500
C	4.29677500	1.45204100	0.07911800

C	3.22859700	2.39433100	0.08025300
C	1.90421300	1.95668600	0.30565400
C	0.85072700	2.89086400	0.05862300
C	1.20515000	4.25146400	-0.01735700
C	2.52775400	4.70485000	-0.13441200
C	3.51404000	3.76300200	-0.15040300
N	0.06671300	5.02658900	-0.04669000
C	0.02881500	6.44222200	-0.06421900
C	0.69824900	7.16498500	0.91685500
C	0.66624700	8.55100500	0.89098100
C	-0.04522000	9.21582100	-0.09866200
C	-0.71956300	8.48964500	-1.07099000
C	-0.67761800	7.10366900	-1.06245000
C	-1.02843100	4.19111200	-0.05521800
C	-0.60150700	2.85000500	-0.09690600
C	-1.60285700	1.85435800	-0.31851700
C	-1.35252700	0.51414400	-0.80336000
C	-0.14226500	0.16433100	-1.43383000
C	0.10900900	-1.12440000	-1.83028900
C	-0.85236200	-2.12262700	-1.62011400
C	-2.07685800	-1.79577100	-1.09302900
C	-2.36229300	-0.47077600	-0.71309100
C	-3.69000300	-0.08802500	-0.29174100
C	-4.70620000	-1.08052800	-0.18310600
C	-5.52737400	-1.95854100	-0.10481500
C	-6.48028800	-3.01304300	-0.02192800
C	-7.79054000	-2.75924700	0.39437700
C	-8.71512100	-3.79032500	0.47208000
C	-8.32691300	-5.08157800	0.13172100
C	-7.03212400	-5.34448300	-0.28167200
C	-6.09704200	-4.31749200	-0.36300100
O	-10.00775500	-3.63401300	0.86592700
C	-3.96433200	1.22661800	-0.07169100
C	-2.94899100	2.22549700	-0.10105500
C	-3.30758200	3.58243400	0.09400300
C	-2.37357400	4.57551200	0.05179800
C	6.99786800	-3.84730300	0.37823300
C	8.04683100	-4.75127100	0.28917300
H	10.48026600	-2.64026200	-0.73649900
H	8.60006700	-1.02881100	-0.57590500
H	-0.24432900	0.94983400	1.61827800
H	-0.57934200	-1.35144900	2.36713700
H	1.21197100	-3.04292600	2.02914800
H	3.38516200	-2.34716800	1.09273000
H	5.29234600	1.80435900	-0.16043400

H	2.74258500	5.75769900	-0.25541700
H	4.54533900	4.05543700	-0.30336700
H	1.23229800	6.63359400	1.69421300
H	1.18901600	9.11214900	1.65429500
H	-0.07396800	10.29722000	-0.11212900
H	-1.27078000	9.00314800	-1.84770800
H	-1.18203700	6.52554300	-1.82611900
H	0.59708500	0.93109900	-1.61247500
H	1.05229300	-1.36814700	-2.30157800
H	-0.64199200	-3.14423300	-1.90973200
H	-2.85151600	-2.54503600	-0.98270100
H	-8.05140700	-1.74309200	0.64957800
H	-9.05842900	-5.87575700	0.19701600
H	-6.76070700	-6.35763800	-0.53953800
H	-4.97764600	1.52981000	0.16068600
H	-4.35308600	3.82281300	0.24121900
H	-2.64478300	5.61806900	0.14567800
H	6.02139800	-4.19172200	0.69257300
H	7.87245900	-5.78830700	0.53528600
C	-4.39594400	-5.78167600	-1.11521400
H	-4.48878400	-6.47430100	-0.27413700
H	-4.97260600	-6.16200800	-1.96286800
H	-3.35018900	-5.69838300	-1.39936300
C	-10.42739000	-2.33669100	1.21787800
H	-11.47358300	-2.42032200	1.49972300
H	-9.85324000	-1.95017800	2.06478900
H	-10.33297700	-1.64664500	0.37455200
O	-4.81008400	-4.48408600	-0.75692500
C	10.24257800	-6.47406900	0.07117000
H	9.51059100	-6.94830000	-0.58803000
H	11.21727000	-6.92777600	-0.08556000
H	9.93833200	-6.61631200	1.11155400

N,*N*'-Dimethylpyromellitic diimide (Figure S1, right)

N	3.36170900	0.00178400	0.00021900
C	2.58763600	1.15923300	0.00013600
C	1.16387800	0.69502100	-0.00005300
C	0.00000000	1.44356300	-0.00020800
C	-1.16387700	0.69502100	-0.00037600
C	-1.16396300	-0.69433900	-0.00034800
C	-2.58873900	-1.15372200	-0.00053600
N	-3.36170900	0.00178400	-0.00014300
C	-2.58763700	1.15923300	-0.00063400
O	-3.00311800	2.28373600	-0.00004300
O	-3.01546200	-2.27451000	0.00006500

C	0.00000000	-1.44279100	-0.00015800
C	1.16396400	-0.69433900	-0.00003200
C	2.58873900	-1.15372200	0.00014200
O	3.01546300	-2.27450900	0.00021200
O	3.00311800	2.28373600	0.00013300
H	0.00000000	2.52574800	-0.00022600
H	0.00000000	-2.52497200	-0.00013500
C	-4.80842000	-0.01418000	0.00059000
H	-5.17642800	-0.53114100	-0.88439600
H	-5.17575300	-0.52835500	0.88749000
H	-5.14845700	1.01840700	-0.00085000
C	4.80841900	-0.01418000	0.00037800
H	5.17601400	-0.52944200	0.88653300
H	5.17616700	-0.53005500	-0.88535500
H	5.14845500	1.01840800	0.00006300

References

[S1] M. J. Frisch, G. Trucks, H. B. Schlegel, et al., *Gaussian 16*, Revision C.01 Gaussian, Inc., 2016.

[S2] H. Yokoyama, *MoleditPy — A Python-based molecular editing software*, Version 1.18.1, Zenodo, 2026, DOI: https://doi.org/10.5281/zenodo.17268532.

[S3] M. S. H. Salem, R. Sharma, S. Suzuki, Y. Imai, M. Arisawa, S. Takizawa, "Impact of Helical Elongation of Symmetric Oxa[*n*]Helicenes on Their Structural, Photophysical, and Chiroptical Characteristics," *Chirality* **36**, (2024): e23673.

[S4] K. Nonaka, H. Sakai, R. Mori, et al., "Quenching Mechanism in Rotaxane Mechanophores: Insights from Acene-Based Luminophores," *Chemical Science* **16**, (2025): 21534.

[S5] J. H. Hunter, L. Prendergast, L. F. Valente, A. Madin, G. Pairaudeau, M. J. Waring, "High Fidelity Suzuki–Miyaura Coupling for the Synthesis of DNA Encoded Libraries Enabled by Micelle Forming Surfactants," *Bioconjugate Chemistry* **31**, (2020): 149.

[S6] T. Muramatsu, Y. Okado, H. Traeger, et al., "Rotaxane-Based Dual Function Mechanophores Exhibiting Reversible and Irreversible Responses," *Journal of the American Chemical Society* **143**, (2021): 9884.

[S7] K. Hiratsuka, T. Muramatsu, T. Seki, C. Weder, G. Watanabe, Y. Sagara, "Tuning the Mechanoresponsive Luminescence of Rotaxane Mechanophores by Varying the Stopper Size," *Journal of Materials Chemistry C* **11**, (2023): 3949.
